# FTO-dependent m^6^A modification of *Plpp3* in circSCMH1-regulated vascular repair and functional recovery following stroke

**DOI:** 10.1038/s41467-023-36008-y

**Published:** 2023-01-30

**Authors:** Bin Li, Wen Xi, Ying Bai, Xue Liu, Yuan Zhang, Lu Li, Liang Bian, Chenchen Liu, Ying Tang, Ling Shen, Li Yang, Xiaochun Gu, Jian Xie, Zhongqiu Zhou, Yu Wang, Xiaoyu Yu, Jianhong Wang, Jie Chao, Bing Han, Honghong Yao

**Affiliations:** 1 Department of Pharmacology, Jiangsu Provincial Key Laboratory of Critical Care Medicine, School of Medicine, Southeast University, Nanjing, Jiangsu China; 2 Jiangsu Key Laboratory of Molecular and Functional Imaging, Department of Radiology, Zhongda Hospital, Medical School of Southeast University, Nanjing, Jiangsu China; 3 National Research Facility for Phenotypic and Genetic Analysis of Model Animals, Kunming Institute of Zoology, Chinese Academy of Sciences, Kunming, Yunnan China; 4 Department of Physiology, Jiangsu Provincial Key Laboratory of Critical Care Medicine, School of Medicine, Southeast University, Nanjing, Jiangsu China; 5 Co-innovation Center of Neuroregeneration, Nantong University, Nantong, Jiangsu China; 6 Institute of Life Sciences, Key Laboratory of Developmental Genes and Human Disease, Southeast University, Nanjing, Jiangsu China

**Keywords:** Stroke, Molecular biology, Molecular medicine, Stroke

## Abstract

Vascular repair is considered a key restorative measure to improve long-term outcomes after ischemic stroke. *N*^6^-methyladenosine (m^6^A), the most prevalent internal modification in eukaryotic mRNAs, functionally mediates vascular repair. However, whether circular RNA SCMH1 (circSCMH1) promotes vascular repair by m^6^A methylation after stroke remains to be elucidated. Here, we identify the role of circSCMH1 in promoting vascular repair in peri-infarct cortex of male mice and male monkeys after photothrombotic (PT) stroke, and attenuating the ischemia-induced m^6^A methylation in peri-infarct cortex of male mice after PT stroke. Mechanically, circSCMH1 increased the translocation of ubiquitination-modified fat mass and obesity-associated protein (FTO) into nucleus of endothelial cells (ECs), leading to m^6^A demethylation of phospholipid phosphatase 3 (*Plpp3*) mRNA and subsequently the increase of *Plpp3* expression in ECs. Our data demonstrate that circSCMH1 enhances vascular repair via FTO-regulated m^6^A methylation after stroke, providing insights into the mechanism of circSCMH1 in promoting stroke recovery.

## Abstract (web summary)

The mechanisms behind how vascular repair is regulated after ischemic stroke are yet to be elucidated. Here, the authors describe that a circular RNA interacts with FTO to promote vascular repair following stroke in mice and primates via mediating m6 A modification.

## Introduction

Characterized as neurological deficits caused by cerebrovascular occlusion, ischemic stroke is a leading cause of adult disability and severely compromises the quality of life of patients^1,2^. To date, there is a lack of therapeutic agents to promote stroke recovery and improve long-term outcomes^3^. Growing evidence demonstrates that vascular repair after stroke could improve long-term neurological function and was strongly associated with high survival rates in animal models and stroke patients^4–6^. Vascular repair in the ischemic border zone is thus widely considered a key restorative measure to improve long-term outcomes after ischemic stroke. Investigating the therapeutic strategy of promoting vascular repair after ischemic stroke is an urgent research area that could benefit a large number of patients.

Circular RNAs (circRNAs) are a type of endogenous non-coding RNA molecule. They have a circular structure created by back-splicing and covalently closed continuous loops^7–9^. Our previous and other studies have demonstrated that circRNAs are abundant in the brain, and multiple circRNAs have been functionally implicated in ischemic stroke, such as circHECTD1, circDLGAP4, circTLK1, circFoxO3, and circUCK2^10–14^. Mounting evidence indicated that extracellular vesicles (EVs) cross the blood–brain barrier (BBB) and deliver functional cargoes to recipient cells to modulate gene expression^15,16^. EV-circSCMH1 has been shown to improve motor functional recovery after stroke via enhancement of neuroplasticity, inhibition of glial reactivity, and peripheral immune cell infiltration^17^. However, whether circSCMH1 enhances vascular repair after ischemic stroke and the downstream mechanisms remain unclear.

*N*^6^-methyladenosine (m^6^A) is the most prevalent post-transcriptional modification in messenger RNA (mRNA), affecting mRNA splicing, transport, stability, and translation^18,19^. The reversible m^6^A modification is catalyzed by a methyltransferase complex comprised of methyltransferase-like 3 (METTL3), methyltransferase-like 14 (METTL14), and Wilms tumor 1-associated protein (WTAP), and it can be removed by m^6^A demethylases fat mass and obesity-associated protein (FTO), and alkB homolog 5 (ALKBH5)^20^. M^6^A functions are performed by reader proteins, including the YT521-B homology (YTH) domain family, heterogeneous nuclear ribonucleoprotein (HNRNP) family, and insulin-like growth factor 2 mRNA-binding proteins (IGF2BPs), which selectively recognize and directly or indirectly bind to the m^6^A motif to affect mRNA functions^21^. M^6^A is more abundant in the nervous system than in other organs, and its overall abundance increases from the embryonic brain to the adult brain, suggesting an important role in both brain development and neurological diseases such as stroke^22,23^.

Previous study indicated that transient focal ischemia of the brain significantly increased the global m^6^A levels in the mouse cerebral cortex^23^. Growing evidence also demonstrated that in the early stage of acute ischemic stroke, the stress granule formation was increased by METTL3-mediated m^6^A methylation^24^. Additionally, overexpression of FTO attenuated neuronal death induced by oxygen-glucose deprivation/reoxygenation^25^. Regarding the vascular repair, METTL3 enhanced retinal angiogenesis after hypoxia through the m^6^A modification of targets low-density lipoprotein receptor-related protein 6 (*LRP6*) and disheveled 1 (*DVL-1*)^26^, whereas decreased m^6^A modification of desmoplakin (*DSP*) mRNA by WTAP deficiency inhibited angiogenesis of ECs in the brain arteriovenous malformations^27^. However, the functional link between m^6^A methylation and vascular repair after ischemic stroke remains unknown.

This study aims to investigate the functional significance of m^6^A methylation in circSCMH1-mediated vascular repair during stroke recovery. We confirm the role of circSCMH1 in improving vascular repair and decreasing the level of m^6^A methylation after stoke. Mechanically, circSCMH1 binds with FTO and facilitates the nuclear translocation of FTO in ECs, leading to the decrease of m^6^A methylation of phospholipid phosphatase 3 (*Plpp3*) mRNA and subsequently enhancing the *Plpp3*-encoded lipid phosphate phosphatase 3 (LPP3) level. Our findings indicated that circSCMH1 enhanced vascular repair via FTO-regulated m^6^A methylation, providing a previously overlooked mechanism of circSCMH1 in promoting stroke recovery.

## Results

### CircSCHM1 improved vascular repair during ischemic stroke recovery

**Fig. 1:**
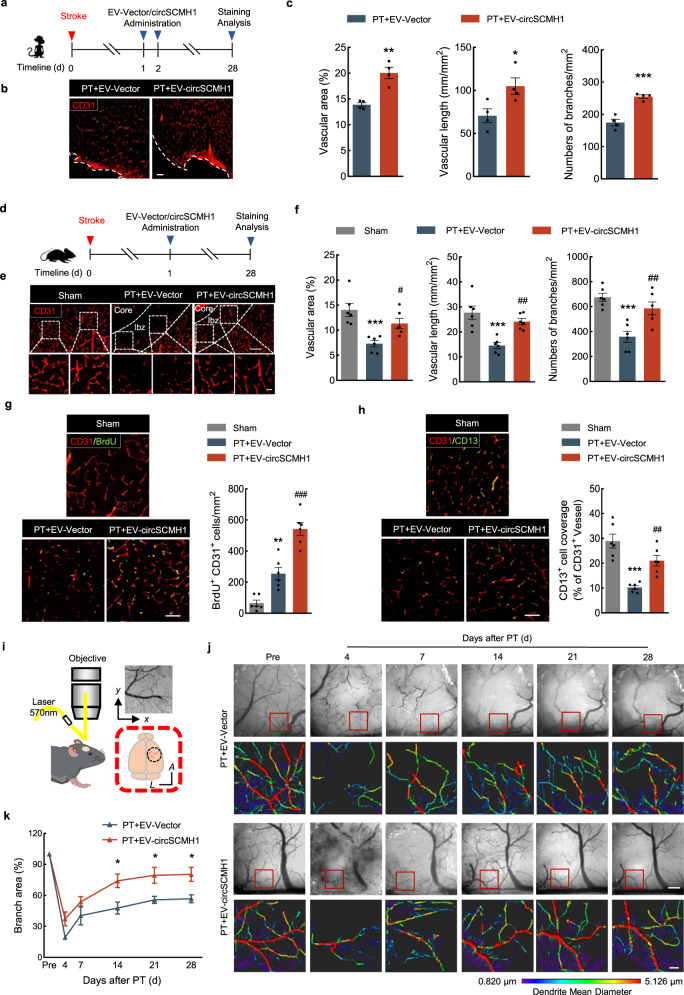
CircSCMH1 promoted vascular repair during stroke recovery. **a** Schematic of EV-circSCMH1 administration and staining analysis in monkeys. **b**, **c** Representative images with CD31 staining showing blood vessels in the peri-infarct cortex at day 28 after PT in monkeys, followed by the analysis of vascular area fraction, total vascular length, and the number of branches. Scale bars, 100 μm. *n* = 4 monkeys/group. ^*^P = 0.0337, ^**^P = 0.0018, ^***^P = 0.0004 versus PT + EV-Vector. **d** Schematic of EV-circSCMH1 administration and staining analysis in mice. **e**, **f** Representative images with CD31 staining showing blood vessels in the peri-infarct cortex at day 28 after PT in mice, followed by the analysis of vascular area fraction, total vascular length, and the number of branches. Scale bars, 100 μm (overview), 20 μm (insets). *n* = 6 mice/group. ^***^P = 0.0008 (vascular area), ^***^P = 0.0004 (vascular length, numbers of branches) versus sham; ^#^*P* = 0.0253 (vascular area), ^##^*P* = 0.0041 (vascular length), ^##^*P* = 0.0043 (numbers of branches) versus PT + EV-Vector. **g** Representative images and quantification of newly generated BrdU^+^/CD31^+^ endothelial cells in the peri-infarct cortex at day 28 after PT. Scale bars, 20 μm. *n* = 6 mice/group. ^**^P = 0.0018 versus sham; ^###^*P* < 0.0001 versus PT + EV-Vector. **h** Representative images and quantification of CD13^+^ pericyte coverage on CD31^+^ microvessels in the peri-infarct cortex at day 28 after PT. Scale bars, 20 μm. *n* = 6 mice/group. ^***^P < 0.0001 versus sham; ^##^*P* = 0.0048 versus PT + EV-Vector. **i** Schematic of long-term optical imaging platform. **j**, **k** Representative images obtained by LEDs for HBT after PT, followed by reconstruction and analysis of branch area fraction using Imaris x64 9.0.0. Scale bars, 100 μm (overview), 20 μm (insets). *n* = 4 mice/group. A indicates anterior; L indicates lateral. ^*^P = 0.0110 (14d), ^*^P = 0.0212 (21d, 28d) versus PT + EV-Vector. The data in **c** were expressed as mean ± SEM; Student’s *t*-test (two-sided). The data in **f**–**h** were expressed as mean ± SEM; one-way ANOVA followed by Holm–Sidak post hoc multiple comparison test. The data in **k** were expressed as mean ± SEM; two-way repeated-measures ANOVA followed by Holm–Sidak post hoc multiple comparison test. Components of this figure were created using Servier Medical Art templates, which are licensed under a Creative Commons Attribution 3.0 Unported License; https://smart.servier.com. Source data are provided as a Source Data file. BrdU 5-bromo-2’-deoxyuridine, CD13 Aminopeptidase N, d day, ibz ischemic border zone, Pre pre-injury.

CircSCMH1 has been shown to significantly enhance neuronal plasticity and functional recovery after stroke^17^. To investigate the potential involvement of circSCMH1 in vascular repair during ischemic stroke, circSCMH1 was delivered to the brain of photothrombotic (PT) nonhuman primates by EVs as described in Fig. 1a. At day 28 after PT model induction, EV-circSCMH1-treated monkeys displayed significantly increased vascular area, total vascular length, and branch numbers compared to the EV-Vector group (Fig. 1b, c). We conducted similar studies in a murine PT stroke model and again detected significant differences at day 28 after PT model induction in vascular area, total vascular length, and branch numbers between the EV-Vector group and EV-circSCMH1 group (Fig. 1d–f). To confirm the expression of circSCMH1 in mouse brain endothelial cells (ECs), the immunofluorescence was performed in sections of mouse brain using markers for vascular EC (CD31^+^), suggesting that the red-colored DiI^+^ EV was within CD31^+^ vascular ECs in the brain (Supplementary Fig. 1a). Next, brain vascular ECs were sorted by flow cytometry from the peri-infarct cortex of PT mice with EV-circSCMH1 injection. At day 7 after PT model induction, the level of circSCMH1 in isolated ECs was significantly decreased compared with the sham group, which was alleviated by EV-circSCMH1 treatment (Supplementary Fig. 1b). In conclusion, these findings indicate that the injection of EV-circSCMH1 increases the circSCMH1 level of ECs and promotes vascular repair in the peri-infarct area of PT model mice.

To investigate the effect of circSCMH1 on newly formed microvessels, endothelial proliferation was examined by 5-bromo-2′-deoxyuridine (BrdU) immunostaining. In PT mice that received EV-circSCMH1, the numbers of BrdU^+^/CD31^+^ EC were increased compared with PT mice that received with EV-Vector (Fig. 1g), suggesting circSCMH1 contributes to vessel regeneration after PT stroke. Additionally, by testing the co-localization of endothelial (CD31) and pericyte (CD13) markers, the pericyte coverage in the peri-infarct region was higher in EV-circSCMH1-treated animals, suggesting a more mature vessel network when circSCMH1 is present (Fig. 1h). To monitor vascular repair in the peri-infarct area in vivo, we used a custom-built optical imaging platform to examine the total hemoglobin (HBT) at λ = 570 nm in the cortex of live mice. In PT mice treated with EV-circSCMH1, the vascular branch area in the 2D reconstructed HBT data was increased compared with mice received with EV-Vector at day 14, 21, and 28 after PT model induction (Fig. 1i–k). These findings reveal that EV-circSCMH1 promotes the recovery of injured vessels from the peri-infarct area after PT model induction.

Next, we performed multiphoton microscopy imaging in vivo to observe cerebrovascular perfusion after PT model induction (tail intravenous injection of FITC-dextran of 2,000,000 Da). This analysis displayed that the density of perfused cortical microvessels was significantly increased in PT mice after EV-circSCMH1 as compared to EV-Vector at days 7 and 21 after PT model induction (Supplementary Fig. 2a, b). All those results indicate that EV-delivered circSCMH1 promotes vessel regeneration and perfusion, thus facilitating vascular repair in the peri-infarct area after PT stroke.

Vascular repair has been shown to promote the re-establishment of BBB integrity following stroke^28^, we, therefore, examined the potential effect(s) of circSCMH1 on BBB integrity following PT stroke. EV-circSCMH1 significantly reduced perivascular Evans blue dye and IgG extravasation compared with the EV-Vector group after PT model induction (Supplementary Fig. 3a–c). Decreased expression of tight junction proteins (ZO-1, Occludin, and Claudin-5) were significantly attenuated by EV-circSCMH1 in PT mice compared with EV-Vector administration (Supplementary Fig. 3d). Collectively, these findings indicate that BBB disruption in the peri-infarct cortex is repaired by circSCMH1 after stroke.

### M^6^A modification was increased in AIS patients and PT mice

**Fig. 2:**
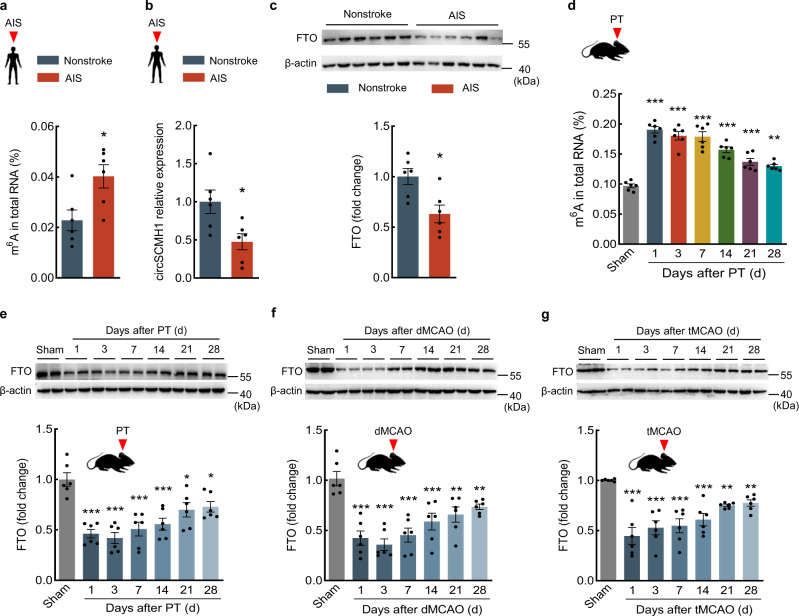
Increased m^6^A modification and decreased FTO in AIS patients and PT mice. **a**, **b** Total RNA was extracted from the somatosensory cortex of AIS or nonstroke patients. The m^6^A level was determined as the percentage of all adenosine residues in RNA (**a**). The level of circSCMH1 was measured by qPCR (**b**). There were six individuals/group. ^*^P = 0.0183 (**a**), ^*^P = 0.0184 (**b**) versus nonstroke. **c** Western blotting analysis of FTO in the somatosensory cortex of AIS and nonstroke patients. There were six individuals/group. ^*^P = 0.0105 versus nonstroke. **d** Total RNA was extracted from the peri-infarct cortex of PT mice, m^6^A levels were determined as the percentage of all adenosine residues in RNA. *n* = 6 mice/group. ^**^P = 0.0018 (28d), ^***^P = 0.0002 (21d), ^***^P < 0.0001 (1d, 3d, 7d, 14d) versus sham. **e**–**g** Western blotting analysis of FTO in the peri-infarct cortex of mice after PT (**e**), dMCAO (**f**), and tMCAO (**g**) stroke. Two representative immunoblots were presented from 6 mice/group. ^*^P = 0.0186 (21d), ^*^P = 0.0408 (28d), ^***^P < 0.0001 (1d, 3d, 7d), ^***^P = 0.0001 (14d) versus sham in **e**. ^**^P = 0.0012 (21d), ^**^P = 0.0057 (28d), ^***^P < 0.0001 (1d, 3d, 7d), ^***^P = 0.0002 (14d) versus sham in **f**. ^**^P = 0.0064 (21d), ^**^P = 0.0070 (28d), ^***^P < 0.0001 (1d, 3d, 7d, 14d) versus sham in **g**. The data in **a**–**c** were expressed as mean ± SEM; Student’s *t*-test (two-sided). The data in **d**–**g** were expressed as mean ± SEM; one-way ANOVA followed by Holm–Sidak post hoc multiple comparison test. Source data are provided as a Source Data file. AIS acute ischemic stroke, d day, dMCAO distal middle cerebral artery occlusion, FTO fat mass and obesity-associated protein, m^6^A *N*^6^-methyladenosine, PT photothrombotic, tMCAO transient middle cerebral artery occlusion.

Given the known involvement of m^6^A methylation in vascular repair^26,27^, we then collected the tissues of the somatosensory cortex from AIS patients and nonstroke controls, and the detailed information is included in Supplementary Table 1. The results showed that the level of m^6^A methylation was significantly increased, and the level of circSCMH1 was significantly decreased in the somatosensory cortex of AIS patients compared with nonstroke controls (Fig. 2a, b). In addition, we further detected the expression of m^6^A-related methylases and demethylases, including METTL3, METTL14, WTAP, FTO, and ALKBH5. The level of FTO was significantly decreased in the somatosensory cortex of AIS patients compared with nonstroke controls (Fig. 2c), whereas there were no significant differences in the levels of METTL3, METTL14, WTAP, and ALKBH5 (Supplementary Fig. 4a, b).

In the mouse PT stroke model, the colorimetric analysis by m^6^A quantification kit revealed a significant increase in the m^6^A level of total RNAs after PT model induction (Fig. 2d). Western blotting showed that there was a significantly decreased level of FTO after PT model induction (Fig. 2e). Additionally, another two-stroke mouse models, distal middle cerebral artery occlusion (dMCAO) and transient middle cerebral artery occlusion (tMCAO), were also used to examine the level of FTO. The level of FTO was significantly decreased in the peri-infarct cortex of the dMCAO and tMCAO stroke mice (Fig. 2f, g). Together, these findings from human AIS patient and multiple murine stroke models support that stroke increases the m^6^A level in the peri-infarct cortex, which is plausible given the concomitant decrease in the FTO level in these samples.

### CircSCMH1 decreased m^6^A modification and bound with FTO

**Fig. 3:**
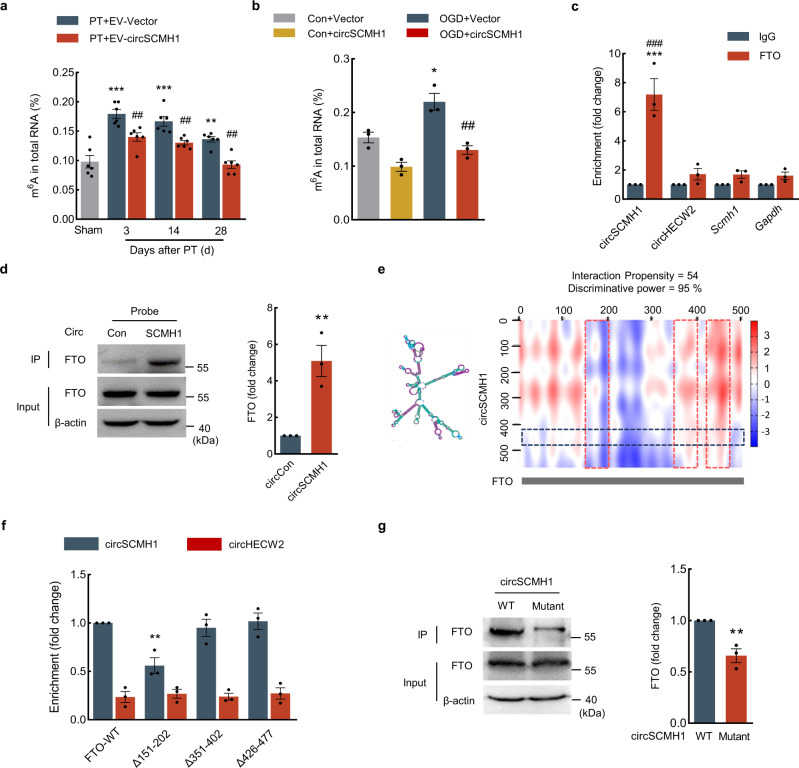
CircSCMH1 decreased m^6^A modification and bound with FTO. **a** Total RNA was extracted from the peri-infarct cortex of PT mice, m^6^A levels were determined as the percentage of all adenosine residues in RNA. *n* = 6 mice/group. ^**^P = 0.0072 (28d), ^***^P < 0.0001 (3d, 14d) versus the sham; ^##^*P* = 0.0056 (3d), ^##^*P* = 0.0097 (14d), ^##^*P* = 0.0024 (28d) versus the PT + EV-Vector. **b** Total RNA was extracted from the primary mouse brain microvascular ECs treated with circSCMH1 plasmid at 12 h after OGD, and m^6^A levels were determined as the percentage of all adenosine residues in RNA. Data were presented by three independent experiments. ^*^P = 0.0231 versus Con+Vector; ^##^*P* = 0.0051 versus OGD + Vector. **c** Interaction between circSCMH1 and FTO was detected by RNA-binding immunoprecipitation in the primary brain microvascular ECs. Data were presented by three independent experiments. ^***^P < 0.0001 versus FTO pull-down of circHECW2; ^###^*P* < 0.0001 versus FTO pull-down of *Gapdh* mRNA. **d** Interaction between circSCMH1 and FTO was measured by RNA pull-down assay in the primary mouse brain microvascular ECs. Data were presented by three independent experiments. ^**^P = 0.0085 versus the circCon probe. **e** Prediction of circSCMH1-FTO interaction by catRAPID algorithm. **f** The interaction between circSCMH1 and FTO was validated by RNA immunoprecipitation in bEnd.3 cells with WT FTO and mutant FTO. Data were presented by three independent experiments. ^**^P = 0.0022 versus circSCMH1 in FTO-WT. **g** Western blot analysis of FTO expression in lysates of bEnd.3 cells with circSCMH1 or mutated circSCMH1 (Δ426–477) overexpression following biotinylated circSCMH1 probe pull-down assay. Data were presented by three independent experiments. ^**^P = 0.0069 versus WT. The data in **a**, **c**, **f** were expressed as mean ± SEM; one-way ANOVA followed by Holm–Sidak post hoc multiple comparison test. The data in **b** were expressed as mean ± SEM; two-way ANOVA followed by Bonferroni’s post hoc multiple comparison tests. The data in **d** and **g** were expressed as mean ± SEM; using the Student *t*-test (two-sided). Source data are provided as a Source Data file. Con control, d day, IgG immunoglobulin G, IP immunoprecipitation, WT wild type. Δ151–202: lacking region 151 to 202 amino acids; Δ351–402: lacking region 351 to 402 amino acids; Δ426–477: lacking region 426 to 477 amino acids or nucleic acids.

Given our findings that (i) circSCMH1 enhanced the vascular repair after stroke and (ii) there was a negative correlation (Pearson correlation coefficient *r* = −0.6695, *P* = 0.0173) between circSCMH1 and m^6^A levels in AIS and nonstroke individuals (Supplementary Fig. 5a), we explored potential impacts of circSCMH1 on m^6^A methylation after stroke. The results showed that EV delivery of circSCMH1 to the brain of mice significantly attenuated the increased m^6^A level of total RNAs in peri-infarct areas after PT model induction (Fig. 3a), whereas LV-si-circSCMH1 increased the m^6^A level of total RNAs after PT model induction (Supplementary Fig. 5b). Additionally, in primary brain microvascular ECs and the mouse brain EC line-bEnd.3 cells, oxygen-glucose deprivation (OGD) exposure resulted in a significant increase of the m^6^A level in ECs, which was attenuated by overexpression of circSCMH1 (Fig. 3band Supplementary Fig. 5c).

There was also a positive correlation between circSCMH1 and FTO (Pearson correlation coefficient *r* = 0.5677, *P* = 0.0542) (Supplementary Fig. 5d). Thus, we first examined the effect of circSCMH1 on the expression of FTO. However, western blotting showed no significant difference in FTO level between EV-circSCMH1 and EV-Vector groups after PT stroke (Supplementary Fig. 5e, f). Moreover, *Fto* mRNA from the ECs sorted by flow cytometry was decreased in PT mice, but there was no significant difference between the EV-circSCMH1 and EV-Vector groups (Supplementary Fig. 5g). Western blotting revealed that OGD exposure led to a significantly decreased level of FTO in primary mouse brain microvascular ECs and bEnd.3 cells, and circSCMH1 overexpression did not affect the OGD-induced FTO decrease (Supplementary Fig. 5h, i). Together, the above results suggest that circSCMH1 attenuates the increase of m^6^A level, but do not affect the levels of FTO after stroke.

Previous study showed that FTO exhibited differential substrate preferences in the nucleus versus cytoplasm, the m^6^A was more likely the substrate of FTO in the primary polyadenylated RNA in the cell nucleus^29^. Thus, to investigate whether circSCMH1 effects FTO translocation into nuclear, we first examined the interaction between circSCMH1 and FTO. Compared to the negative control circHECW2 and *Gapdh*, circSCMH1, not *Scmh1*, showed a stronger affinity with FTO in ECs by RNA-binding immunoprecipitation assay (Fig. 3c). The biotinylated probe of circSCMH1 pull-down assay showed that the circSCMH1 probe pulled down more FTO than the circCon probe (Fig. 3d). The catRAPID algorithm was further used to predict the binding region of circSCMH1 in FTO. Three regions of FTO were predicted to have high interaction capacity with circSCMH1: residues 151–202, 351–402, and 426–477. We generated FTO variants lacking residues 151–202, 351–402, or 426–477, and RNA-binding assays showed that there was a decreased level of interaction between circSCMH1 and FTO in the Δ151–202 group compared with the FTO-WT group, suggesting the circSCMH1 and FTO binding interaction requires the presence of residues 151–202 of FTO (Fig. 3e, f). Moreover, we constructed a circSCMH1 mutation plasmid (Δ426–477) without the binding sequence of circSCMH1 (426–477) that interacted with FTO. In comparison with the circSCMH1 mutant variant, the unmodified circSCMH1 exhibited stronger binding with FTO in bEnd.3 cells (Fig. 3g). Thus, these findings indicate that there is an interaction between circSCMH1 and FTO in ECs.

### CircSCMH1 increased the translocation of FTO into the nucleus via ubiquitination

**Fig. 4:**
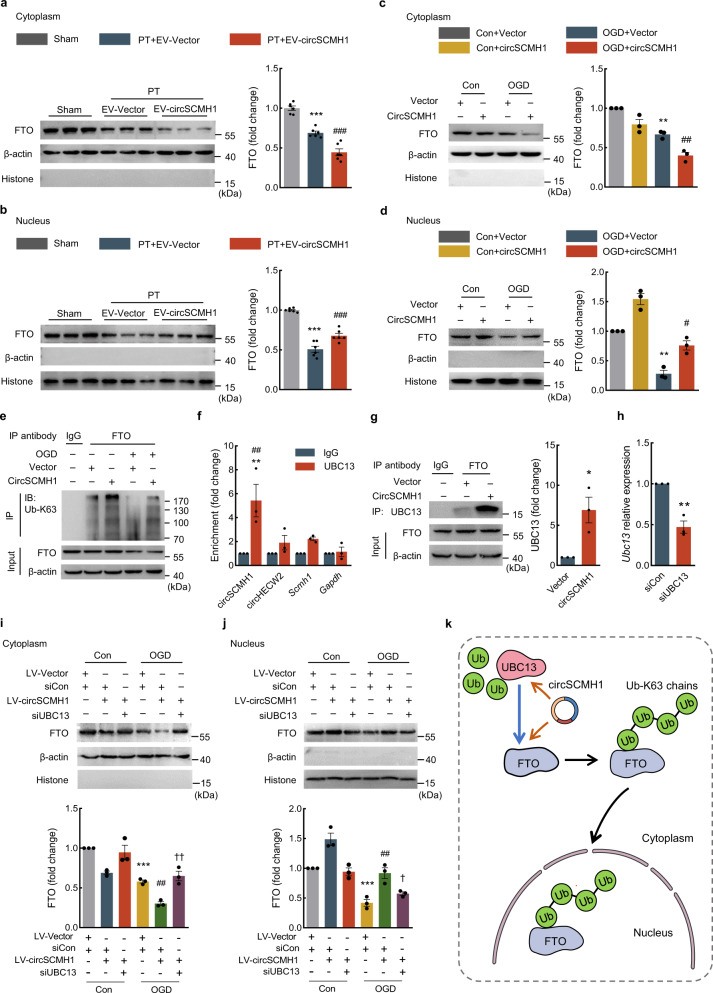
CircSCMH1 altered the subcellular localization of FTO via ubiquitination. **a**, **b** FTO expression in peri-infarct tissue’s cytoplasm (**a**) and nucleus (**b**) at day 28 after PT. Three representative immunoblots were presented from 6 mice/group. ^***^P < 0.0001 (**a**, **b**) versus sham, ^###^*P* < 0.0001 (**a**), ^###^*P* = 0.0008 (**b**) versus PT + EV-Vector. **c**, **d** Representative western blotting of FTO expression in the cytoplasm (**c**) and nucleus (**d**) of the primary mouse brain microvascular ECs at 12 h after OGD. Data were presented by three independent experiments. ^**^P = 0.0012 (**c**), ^**^P = 0.0029 (**d**) versus Con+Vector; ^#^*P* = 0.0235, (**d**) ^##^*P* = 0.0039 (**c**) versus OGD + Vector. **e** Immunoprecipitation detected Ub-K63 modification of FTO in primary mouse brain microvascular ECs at 12 h after OGD. Data were representative of three independent experiments. **f** Interaction between circSCMH1 and UBC13 was detected by RNA-binding immunoprecipitation in bEnd.3 cells. Data were presented by three independent experiments. ^**^P = 0.0076 versus UBC13 pull-down of circHECW2; ^##^*P* = 0.0011 versus UBC13 pull-down of *Gapdh* mRNA. **g** Immunoprecipitation showed the binding of FTO with UBC13 in bEnd.3 cells. Data were presented by three independent experiments. ^*^P = 0.0210 versus vector. **h** The bEnd.3 cells were transfected with siUBC13, the level of *Ubc13* mRNA was measured by qPCR. Data were presented by three independent experiments. ^**^P = 0.0020 versus siCon. **i**, **j** Western blot analysis of FTO expression in the cytoplasm (**i**) and nucleus (**j**) of bEnd.3 cells with LV-circSCMH1 and siUBC13 at 12 h after OGD. Data were presented by three independent experiments. ^***^P = 0.0004 (**i**), ^***^P = 0.0007 (**j**) versus Con+LV-Vector+siCon; ^##^*P* = 0.0073 (**i**), ^##^*P* = 0.0019 (**j**) versus OGD + LV-Vector + siCon; ^†^*P* = 0.0184 (**j**), ^††^*P* = 0.0018 (**i**) versus OGD + LV-circSCMH1+siCon. **k** Proposed model of the regulatory role of circSCMH1 and UBC13 for FTO translocation into the nucleus. The data in **a**, **b**, **f**, **i**, **j** were expressed as mean ± SEM; one-way ANOVA followed by Holm–Sidak post hoc multiple comparison test. The data in **c**, **d** were expressed as mean ± SEM; two-way ANOVA followed by Bonferroni’s post hoc multiple comparison tests. The data in **g**, **h** were expressed as mean ± SEM; using the Student *t*-test (two-sided). Source data are provided as a Source Data file. Con control, IB immunoblot, siUBC13 *Ubc13* siRNA, Ub-K63 lysine 63-linked ubiquitination.

The fact that circSCMH1 did not affect the level of FTO in the peri-infarct tissues of PT mice (or in ECs after OGD) prompted us to examine the nucleocytoplasmic redistribution of FTO. Intriguingly, western blotting showed that the FTO level was significantly decreased in the cytoplasm and increased in the nucleus in the peri-infarct cortex of PT mice after administration of EV-circSCMH1 compared with EV-Vector (Fig. 4a, b). This finding was confirmed in primary mouse brain microvascular ECs and bEnd.3 cells where the circSCMH1 overexpression decreased the level of FTO in the cytoplasm and increased in the nucleus (Fig. 4c, d and Supplementary Fig. 6a, b).

Given the previously reported knowledge that ubiquitination promotes FTO translocation to the nucleus from the cytoplasm^30^, we next examined the potential effect(s) of circSCMH1 on the ubiquitination of FTO. The lysine 63-linked ubiquitination (Ub-K63) level of FTO was significantly increased by circSCMH1 overexpression in OGD-treated primary mouse brain microvascular ECs (Fig. 4e). UBC13 was a known E2 ubiquitin-conjugating enzyme for Ub-K63^31^. The RNA-binding immunoprecipitation assay revealed that, compared with the negative control circHECW2, circSCMH1 showed stronger affinity to UBC13 in bEnd.3 cells (Fig. 4f). Since circRNAs were shown to act as a scaffold to enhance the binding of ubiquitin enzyme with its target^32^, we next sought to examine whether circSCMH1 enhances the binding of FTO with UBC13. As shown in Fig. 4g, the interaction between FTO and UBC13 was increased in a circSCMH1-treated group compared with the Vector group as determined by co-immunoprecipitation analysis. Moreover, to further explore the effect of UBC13 on FTO transferring into the nucleus, cotransfection of circSCMH1 overexpression lentivirus and UBC13 siRNA were performed and showed that in OGD-exposed bEnd.3 cells, UBC13 knockdown significantly attenuated the circSCMH1-enhanced FTO translocation into the nucleus (Fig. 4h–j). The proposed model of the regulatory role of circSCMH1 and UBC13 for FTO translocation into the nucleus was as illustrated in Fig. 4k. Collectively, these findings indicate that circSCMH1 increases the Ub-K63 of FTO via UBC13 enzyme and promotes the transportation of FTO from the cytoplasm into the nucleus.

### CircSCMH1-regulated m^6^A modification of *Plpp3* mRNA in the peri-infarct cortex of PT mice

Having determined that circSCMH1 decreased the level of m^6^A methylation in PT stroke model, we next explored the downstream methylated genes. Thus, we performed a transcriptome-wide detection of m^6^A modification in the peri-infarct cortex of PT mice. A volcano plot showed that m^6^A levels were significantly elevated at 279 peak regions of transcripts and significantly decreased at 898 peak regions of transcripts after PT model induction; in addition, EV-circSCMH1 significantly increased 213 peak regions and decreased 499 peak regions of transcripts after PT model induction compared with EV-Vector (Supplementary Fig. 7a). The highly over-represented consensus sequence “GGACU” motif was identified using the HOMER algorithm in PT mice after EV-circSCMH1 and EV-Vector treatment, suggesting the successful enrichment of m^6^A-modified mRNA after PT stroke (Supplementary Fig. 7b).

**Fig. 5:**
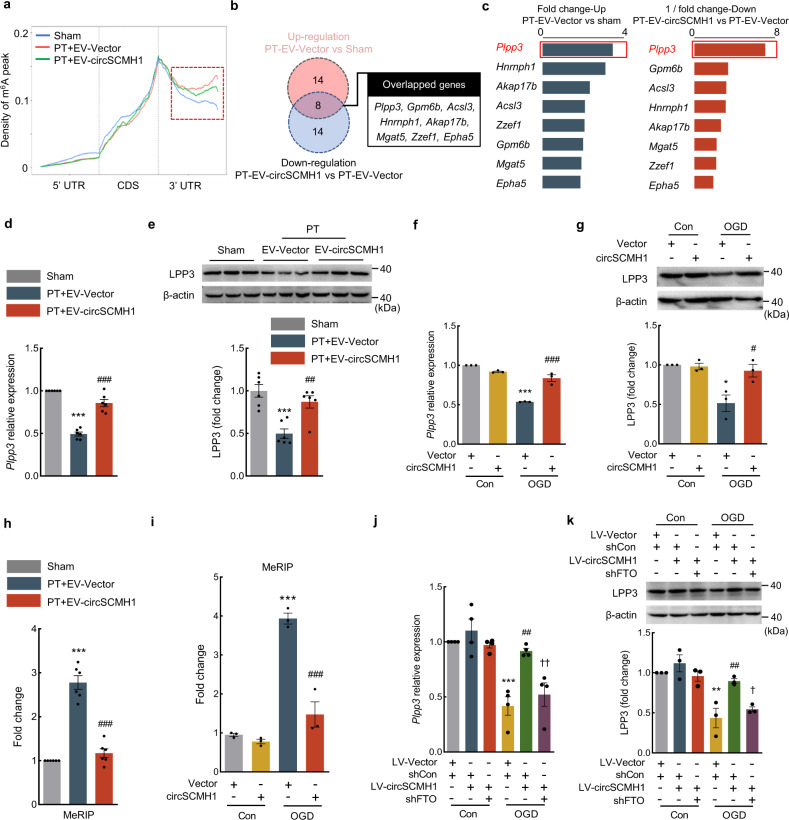
*Plpp3* was one of the critical target genes of circSCMH1-regulated m^6^A modification in the peri-infarct cortex of PT mice. **a** Distribution of m^6^A peaks across 5′-UTR, CDS, and 3′-UTR of mRNA at day 14 after PT. **b** Venn diagram showing numbers of genes with significant changes in expression (up: fold change ≥ 2, *P* < 0.05; down: fold change ≤ 0.5, *P* < 0.05, rescaled hypergeometric test). **c** The m^6^A level of *Plpp3* transcript was regulated in PT mice after EV-circSCMH1 administration. **d**, **e** Effect of EV-circSCMH1 on *Plpp3* mRNA (**d**) and LPP3 (**e**) levels in mice at day 14 after PT. *n* = 6 mice/group. Three representative immunoblots were presented from 6 mice/group. ^***^P < 0.0001 (**d**), ^***^P = 0.0003 (**e**) versus sham; ^##^*P* = 0.0030 (**e**), ^###^*P* < 0.0001 (**d**) versus PT + EV-Vector. **f**, **g** Effect of circSCMH1 plasmid on the expression of *Plpp3* mRNA (**f**) and LPP3 (**g**) in primary mouse brain microvascular ECs after OGD. Data were presented by three independent experiments. *P = 0.0171 (**g**), ^***^P < 0.0001 (**f**) versus Con + Vector; ^#^*P* = 0.0375 (**g**), ^###^*P* = 0.0003 (**f**) versus OGD + Vector. **h** Specific primers against m^6^A peak were designed to amplify m^6^A peak of *Plpp3* transcript in RNA from the peri-infarct cortex of mice at day 14 after PT. *n* = 6/each group. ^***^P < 0.0001 versus the sham; ^###^*P* < 0.0001 versus the PT + EV-Vector. **i** Specific primers against the m^6^A peak were designed to amplify the m^6^A peak of *Plpp3* transcript in bEnd.3 cells. Data were presented by three independent experiments. ^***^P = 0.0002 versus Con + Vector; ^###^*P* = 0.0007 versus OGD + Vector. **j**, **k** The bEnd.3 cells were transfected with LV-circSCMH1 and shFTO. The *Plpp3* mRNA (**j**) and LPP3 (**k**) was detected at 12 h after OGD. Data were presented by 4 (**j**) or 3 (**k**) independent experiments. **P = 0.0020 (**k**), ^***^P = 0.0003 (**j**) versus Con + LV-Vector + shCon; ^##^*P* = 0.0012 (**j**), ^##^*P* = 0.0087 (**k**) versus OGD + LV-Vector + shCon; ^†^*P* = 0.0408 (**k**), ^††^*P* = 0.0083 (**j**) versus OGD + LV-circSCMH1 + shCon. The data in **d**, **e**, **h**, **j**, **k** were expressed as mean ± SEM; one-way ANOVA followed by Holm–Sidak post hoc multiple comparison test. The data in **f**, **g**, **i** were expressed as mean ± SEM; two-way ANOVA followed by Bonferroni’s post hoc multiple comparison tests. Source data are provided as a Source Data file. CDS coding region, shRNA short hairpin RNA, 3′ UTR 3′ untranslated regions, 5′ UTR 5′ untranslated regions.

By analyzing the density of m^6^A peaks, we found that the level of m^6^A modification was increased in the peri-infarct area after PT stroke induction. EV-circSCMH1 treatment suppressed the PT-induced m^6^A modification (Fig. 5a). Additionally, we identified that the significantly increased m^6^A peaks focused on 14 transcripts after PT model induction in comparison to sham mice (fold change ≥2; *P* < 0.05), and those significantly decreased peaks concentrated upon 14 transcripts after EV-circSCMH1 treatment compared with EV-Vector treatment in PT mice (fold change ≤0.5; *P* < 0.05), respectively (Fig. 5b). There were eight overlapping transcripts between the two comparisons, which might be the target transcripts involved in circSCMH1-mediated vascular repair after PT (Fig. 5c).

Knowing that m^6^A is often enriched in 3’-UTR, and involved in several diverse RNA mechanisms, most notably RNA stability^19,33^, we further investigated whether the changes in m^6^A methylation affected the level of these eight overlapping transcripts. By qPCR detection, only the expression of *Plpp3*, an angiogenesis-associated gene, was significantly decreased after PT surgery and reversed by EV-circSCMH1 treatment (Fig. 5d and Supplementary Fig. 8a–g). The same change was also observed at the protein level of LPP3 by western blotting (Fig. 5e). Overexpression of circSCMH1 also attenuated the decrease of *Plpp3* mRNA and LPP3 in primary mouse brain microvascular ECs after OGD exposure (Fig. 5f, g).

The level of m^6^A modification of *Plpp3* was further analyzed and showed that the increase was attenuated by EV-circSCMH1 after PT model induction in the peak region (chr4: 105232584-105232764) of the 3′-untranslated region (UTR) of *Plpp3* mRNA in the peri-infarct cortex (Fig. 5h). The EV-circSCMH1 also decreased the m^6^A modification of *Plpp3* 3′ UTR region in microvessels isolated from the peri-infarct cortex of PT mice (Supplementary Fig. 9a). This change also observed in bEnd.3 cells, showing that OGD treatment increased the level of m^6^A modification of *Plpp3*, which was attenuated by circSCMH1 overexpression (Fig. 5i).

Moreover, cotransfection of circSCMH1 overexpression lentivirus and FTO short hairpin RNA (shFTO) in OGD-exposed ECs showed that circSCMH1 overexpression attenuated the decreased expression of LPP3 and *Plpp3* mRNA, which was rescued by FTO shRNA (Fig. 5j, k). The mRNA stability of *Plpp3* mRNA was further examined in bEnd.3 cells. In the presence of actinomycin D, a transcription inhibitor, circSCMH1 overexpression significantly inhibited the degradation of *Plpp3* mRNA, whereas FTO knockdown significantly attenuated this process, indicating that m^6^A modification mediated the circSCMH1-enhanced *Plpp3* mRNA stability (Supplementary Fig. 9b).

YTHDF2 is known to regulate mRNA degradation by mediating the lifetime of target transcripts^34,35^. Thus, we designed the YTHDF2 siRNA (Supplementary Fig. 9c) and examined the effects of YTHDF2 knockdown on the degradation of *Plpp3* mRNA. In OGD-treated bEnd.3 cells, FTO knockdown significantly promoted the degradation of *Plpp3* mRNA, whereas YTHDF2 siRNA significantly inhibited this process (Supplementary Fig. 9d). Additionally, knockdown of YTHDF2 significantly increased the level of *Plpp3* mRNA in OGD-treated bEnd.3 cells and primary human microvascular ECs (Supplementary Fig. 9e–g). These findings suggest that YTHDF2 recognizes the m^6^A-modified *Plpp3* mRNA and promotes its degradation.

Next, we sought to examine whether circSCMH1 affected the expression of tight junction proteins via FTO/LPP3 in ECs. CircSCMH1 overexpression attenuated the decreased expression of ZO-1, Occludin, and Claudin-5 in bEnd.3 cells after OGD treatment, which was significantly attenuated by shFTO or shLPP3 (Supplementary Fig. 10a, b). To further examine the effect of *Plpp3*/LPP3 on vascular repair, we used AAV-BR1-Plpp3 to construct LPP3 overexpression in ECs and found that *Plpp3* overexpression in ECs significantly increased vascular area, total vascular length, and branch numbers in the peri-infarct cortex of PT mice (Supplementary Fig. 11a–d). Together, these results suggest that *Plpp3* mRNA may be one of the targets of circSCMH1-mediated vascular repair after stroke by FTO.

### Endothelial-targeted FTO overexpression promoted vascular repair in PT mice

**Fig. 6:**
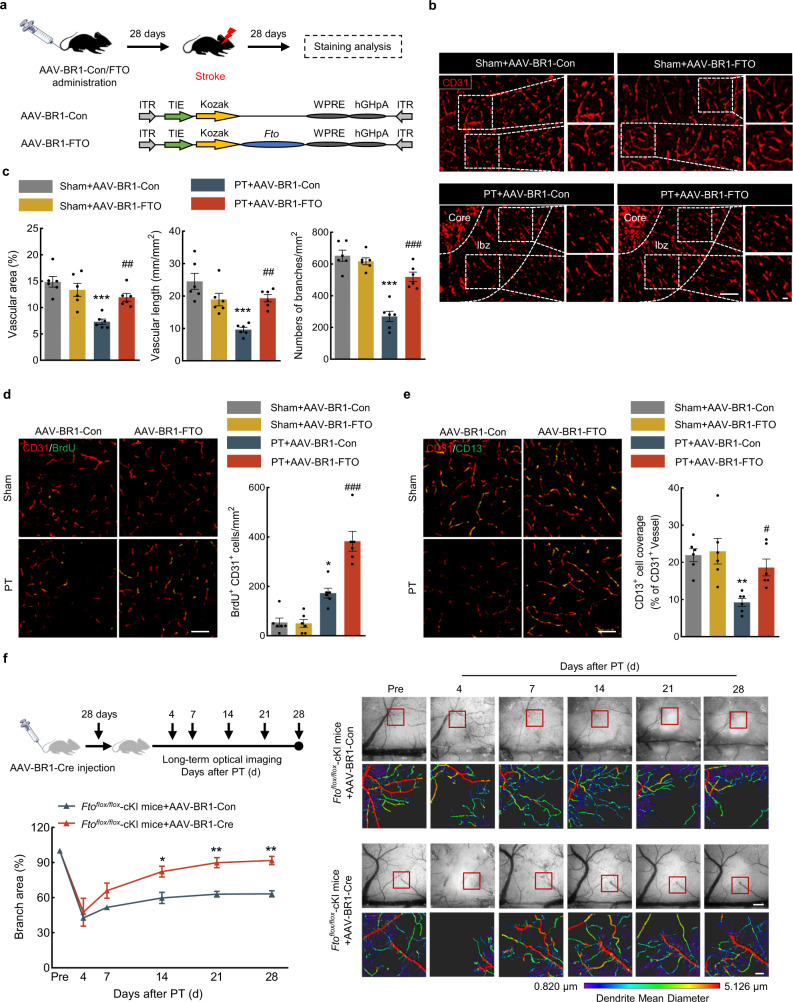
Endothelial-targeted FTO overexpression promoted vascular repair in PT mice. **a** Schematic of AAV-BR1-FTO administration and staining analysis. **b**, **c** Representative images with CD31 staining showing blood vessels in the peri-infarct cortex at day 28 after PT in mice, followed by the analysis of vascular area fraction, total vascular length, and the number of branches. *n* = 6 mice/group. Scale bars, 100 μm (overview), 20 μm (insets). ^***^P < 0.0001 (vascular area, numbers of branches), ^***^P = 0.0001 (vascular length) versus sham + AAV-BR1-Con; ^##^*P* = 0.0075 (vascular area), ^##^*P* = 0.0071 (vascular length), ^###^*P* < 0.0001 (numbers of branches) versus PT + AAV-BR1-Con. **d** Representative images and quantification of newly generated BrdU^+^/CD31^+^ endothelial cells at day 28 after PT. *n* = 6 mice/group. Scale bars, 20 μm. ^*^P = 0.0101 versus sham + AAV-BR1-Con; ^###^*P* < 0.0001 versus PT + AAV-BR1-Con. **e** Representative images and quantification of CD13^+^ pericyte coverage on CD31^+^ microvessels in the peri-infarct cortex at day 28 after PT. Scale bars, 20 μm. *n* = 6 mice/group. ^**^P = 0.0054 versus the sham+AAV-BR1-Con group; ^#^*P* = 0.0485 versus PT + AAV-BR1-Con. **f** Representative images obtained by using LEDs at λ = 570 nm for the HBT at day 4, 7, 14, 21, and 28 after PT in *Fto*^*flox/flox*^ cKI mice with AAV-BR1-Con or AAV-BR1-Cre, followed by 2D reconstruction and analysis of branch area fraction using Imaris x64 9.0.0. *n* = 3 mice/group. Scale bars, 100 μm (overview), 20 μm (insets). ^*^P = 0.0112 (14d), ^**^P = 0.0027 (21d), ^**^P = 0.0019 (28d) versus AAV-BR1-Con. The data in **c**–**e** were expressed as mean ± SEM; two-way ANOVA followed by Bonferroni’s post hoc multiple comparison tests. The data in **f** were expressed as mean ± SEM; two-way repeated-measures ANOVA followed by Holm–Sidak post hoc multiple comparison test. Components of this figure were created using Servier Medical Art templates, which are licensed under a Creative Commons Attribution 3.0 Unported License; https://smart.servier.com. Source data are provided as a Source Data file. AAV adeno-associated virus, cKI conditional knock-in, Con control, ITR inverted terminal repeat, LSL *loxP*-STOP-*loxP,* Pre pre-injury, WPRE woodchuck hepatitis virus post-transcriptional regulation.

Since circSCMH1 enhanced vascular repair and FTO lied downstream of circSCMH1, we next investigated the impacts of FTO in vascular repair following PT model induction. AAV-BR1-FTO was intravenously injected for the specific overexpression of FTO in ECs, as illustrated in Fig. 6a. The level of circSCMH1, *Plpp3*, LPP3, and FTO were further measured after EC-specific FTO overexpression, showing that EC-specific FTO overexpression attenuated the decreased expression of FTO and *Plpp3*/LPP3 after PT stroke, whereas had no effect on the ischemia-induced circSCMH1 decrease (Supplementary Fig. 12a–e). Additionally, EC-specific FTO overexpression reduced the m^6^A methylation of both total RNAs and *Plpp3* mRNA (Supplementary Fig. 12f, g). Immunostaining of CD31 to analyze the vascular repair of the peri-infarct cortex after endothelial-specific overexpression of FTO showed that FTO overexpression in ECs significantly attenuated the decreased level of the vascular area, vascular length, and branch numbers in the peri-infarct cortex of PT mice (Fig. 6b, c). The same effect of vascular repair was observed in the peri-infarct cortex of dMCAO and tMCAO mice after EC-specific overexpression of FTO (Supplementary Fig. 13a–f).

Moreover, AAV-BR1-FTO administration significantly increased the numbers of BrdU^+^/CD31^+^ ECs compared with AAV-BR1-Con administration in PT mice (Fig. 6d). The staining of CD31 and CD13 was performed and demonstrated that FTO overexpression in ECs by AAV-BR1-FTO significantly attenuated the decreased levels of CD13^+^ pericyte coverage in PT mice (Fig. 6e). These results suggest that FTO in ECs contributes to vessel regeneration and constitutes more mature vessel network after PT stroke.

In addition, we found the levels of the ZO-1, Occludin, and Claudin-5 were significantly decreased in the peri-infarct cortex of PT mice, and AAV-BR1-FTO treatment ameliorated this process compared with AAV-BR1-Con treatment (Supplementary Fig. 14a, b), indicating that the specific expression of FTO in ECs enhanced the repair of BBB disruption in the peri-infarct cortex after stroke.

*Fto*^*flox/flox*^ conditional knock-in (cKI) mice were generated to further examine the effect of endothelial-targeted FTO overexpression on vascular repair after PT stroke. To selectively express FTO in ECs, we used Cre recombinase-expressing AAV with TIE promoter to selectively ablate the loxP-flanked STOP cassette in *Hipp 11*, which was positioned upstream of the exogenous *Fto* cDNA, as illustrated in Supplementary Fig. 15. The flow cytometry was used to sort ECs, neurons, astrocytes, and microglia from the brain in *Fto*^*flox/flox*^ cKI mice at day 28 after AAV-BR1-Con or AAV-BR1-Cre injection. We measured the level of *Fto* mRNA by qPCR and found that the *Fto* mRNA in the AAV-BR1-Cre group was significantly increased in ECs compared with the AAV-BR1-Con group, and there was no significant difference in neurons, astrocytes, and microglia (Supplementary Fig. 16a). The increase of FTO in the isolated microvessels was evidenced using western blotting (Supplementary Fig. 16b). Moreover, LPP3 levels were significantly increased in peri-infarct cortex from AAV-BR1-Cre group compared with AAV-BR1-Con group after PT model induction (Supplementary Fig. 16c).

HBT was further analyzed by the optical imaging platform in the peri-infarct cortex of living mice. There was an increased vascular repair in *Fto*^*flox/flox*^ cKI mice injected by AAV-BR1-Cre compared with mice injected by AAV-BR1-Con, reflected by the increased vascular branch area 2D reconstructed according to HBT at day 14, 21, and 28 after PT model induction (Fig. 6f). Therefore, it is indicated that specific expression of FTO in ECs contributes to vascular repair after stroke.

### Endothelial-targeted FTO overexpression promoted motor function recovery after stroke

**Fig. 7:**
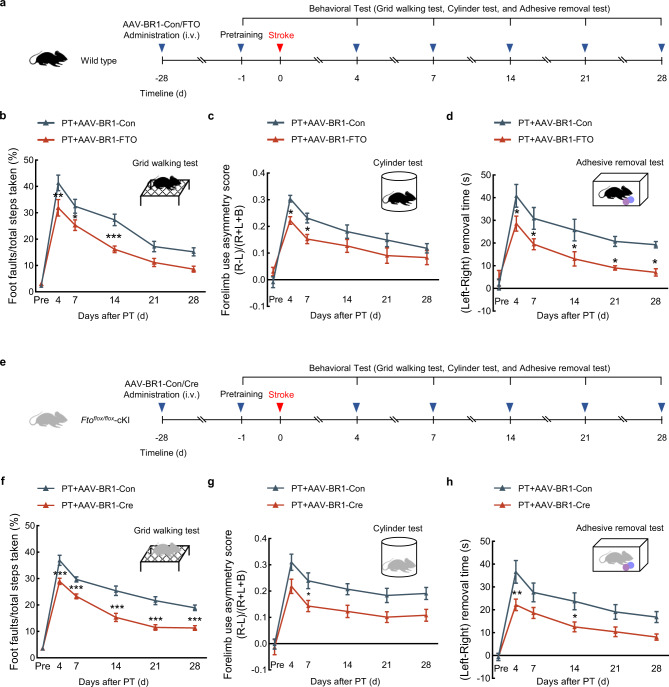
Endothelial-targeted FTO overexpression promoted motor functional recovery in PT mice. **a** Schematic of AAV-BR1-FTO administration and behavioral analysis. **b**–**d** Endothelial-targeted FTO overexpression improved behavioral recovery at different time points after PT as measured by the grid-walking test, cylinder test, and adhesive removal test. L indicates left forepaw in cylinder test; R, right forepaw in cylinder test; B, both forepaws in cylinder test. *n* = 19 mice for PT + AAV-BR1-Con group or PT + AAV-BR1-FTO group. ^*^P = 0.0408 (7d), ^**^P = 0.0039 (4d), ^***^P = 0.0005 (14d) versus the PT + AAV-BR1-Con in **b**. ^*^P = 0.0400 (4d), ^*^P = 0.0444 (7d) versus the PT + AAV-BR1-Con in **c.** ^*^P = 0.0391 (4d), ^*^P = 0.0391 (7d), ^*^P = 0.0347 (14d), ^*^P = 0.0391 (21d), ^*^P = 0.0391 (28d) versus the PT + AAV-BR1-Con in **d**. **e** Schematic of AAV-BR1-Cre administration and behavioral analysis. **f**–**h** *Fto*^*flox/flox*^ cKI mice with endothelial-targeted Cre expression showed behavioral recovery at different time points after PT as measured by the grid-walking test, cylinder test, and adhesive removal test. L indicates left forepaw in cylinder test; R, right forepaw in cylinder test; B, both forepaws in cylinder test. *n* = 11 mice for PT + AAV-BR1-Con group or PT + AAV-BR1-Cre group. ^***^P < 0.0001 (4d), ^***^P = 0.0007 (7d), ^***^P < 0.0001 (14d), ^***^P < 0.0001 (21d), ^***^P < 0.0001 (28d) versus the PT + AAV-BR1-Con in **f**. ^*^P = 0.0442 (7d) versus the PT + AAV-BR1-Con in **g**. ^*^P = 0.0348 (14d), ^**^P = 0.0034 (4d) versus the PT + AAV-BR1-Con in **h**. The data in **b**–**d**, **f**–**h** were expressed as mean ± SEM; two-way repeated-measures ANOVA followed by Holm–Sidak post hoc multiple comparison test. Source data are provided as a Source Data file. AAV adeno-associated virus, circSCMH1 circular RNA SCMH1, d day, FTO fat mass and obesity-associated protein, PT photothrombotic.

Next, we examined the effect of endothelial-targeted FTO overexpression on motor function recovery after stroke. Pre-trained male mice were subjected to PT surgery followed by the administration of AAV-BR1-Con/FTO/Cre. Motor function recovery was evaluated with multiple behavioral tests from day 4 to 28 after PT stroke (Fig. 7a). For the grid-walking task, the intravenous injection of AAV-BR1-FTO significantly decreased the rate of foot faults in comparison with AAV-BR1-Con injection on day 4, 7, and 14 after PT model induction (Fig. 7b). Mice that received AAV-BR1-FTO treatment showed significantly reduced bias at day 4 and 7 after PT model induction in the cylinder test (Fig. 7c). Moreover, the significant recovery effect was observed in the adhesive removal test, reflected by the decreased removal time after AAV-BR1-FTO treatment compared with the AAV-BR1-Con at day 4, 7, 14, 21, and 28 after PT model induction (Fig. 7d).

*Fto*^*flox/flox*^ cKI mice were used to further confirm the above inference. AAV-BR1-Cre performed endothelial-targeted FTO overexpression in *Fto*^*flox/flox*^ cKI mice to evaluate motor function recovery after PT stroke (Fig. 7e). *Fto*^*flox/flox*^ cKI mice injected with AAV-BR1-Cre showed improved performance with reduced foot faults at day 4, 7, 14, 21, and 28 after PT model induction in comparison with the AAV-BR1-Con group in a grid-walking test (Fig. 7f). Similar recovery effect was observed with the cylinder test, where animals treated with AAV-BR1-Cre showed reduced bias after PT model induction (Fig. 7g). For the adhesive removal test, *Fto*^*flox/flox*^ cKI mice administered AAV-BR1-Cre showed decreased removal time compared with AAV-BR1-Con after PT model induction (Fig. 7h).

To further identify the effect of EC-specific FTO overexpression on motor function recovery of dMCAO and tMCAO mice, the pre-trained male mice were administrated with AAV-BR1-Con/FTO, and motor function recovery was evaluated after dMCAO or tMCAO surgery (Supplementary Fig. 17a). For dMCAO model, animals injected AAV-BR1-FTO showed improved performance with reduced foot faults at days 4 and 14 after dMCAO surgery in comparison with the AAV-BR1-Con group in the grid-walking test (Supplementary Fig. 17b). Similar results were recorded with the cylinder test, where animals injected with AAV-BR1-FTO showed significantly reduced bias at day 4, 7, 14, and 28 after stroke (Supplementary Fig. 17c). The same evidence for the therapeutic effect was found in the adhesive removal test, which revealed significant differences in two groups (Supplementary Fig. 17d).

For the tMCAO model, the AAV-BR1-FTO significantly decreased the rate of foot faults in comparison with AAV-BR1-Con injection at days 4, 7, and 14 after the tMCAO model induction (Supplementary Fig. 17e). And similar therapeutic effect were recorded with the cylinder test, where animals injected with AAV-BR1-FTO showed significantly reduced bias at day 14 after stroke (Supplementary Fig. 17f). For the adhesive removal test, there was a significant difference at day 4 and 7 in tMCAO mice from AAV-BR1-FTO group injection (Supplementary Fig. 17g). These data indicated that endothelial-targeted FTO overexpression facilitated motor function recovery in the dMCAO and tMCAO animal models, generalizing our previous results to “acute ischemic stroke”.

Collectively, these results indicate that specific expression of FTO in ECs improves motor function recovery after stroke.

## Discussion

**Fig. 8:**
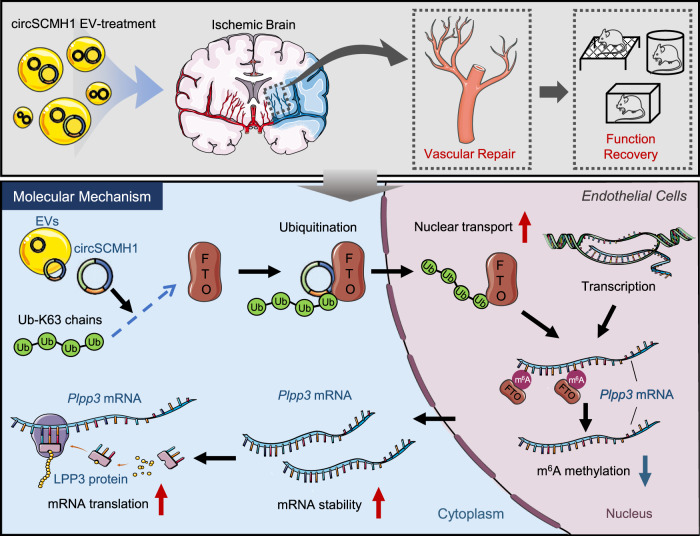
Schematic illustration of the enhancement effect of EV-circSCMH1 on vascular repair after ischemic stroke. Intravenous administration of EV-circSCMH1 after ischemic stroke significantly enhanced vascular repair in mouse and monkey models. The level of m^6^A was significantly increased in the somatosensory cortex of AIS patients and the peri-infarct cortex of mouse stroke models. Endothelial-targeted FTO overexpression significantly improved functional recovery in mice by enhancement of vascular repair. In ECs, circSCMH1 bound with FTO and promoted its transfer to the nucleus by Ub-K63, which decreased the m^6^A methylation of *Plpp3* mRNA. Components of this figure were created using Servier Medical Art templates, which are licensed under a Creative Commons Attribution 3.0 Unported License; https://smart.servier.com. CircSCMH1 circular RNA SCMH1, EV extracellular vesicle, FTO fat mass and obesity-associated protein, m^6^A *N*^6^-methyladenosine, *Plpp3*/LPP3 lipid phosphate phosphatase 3, Ub-K63 lysine 63-linked ubiquitination.

Our current study discovered that m^6^A methylation was increased in stroke patients and multiple mouse stroke models. CircSCMH1 promoted vascular repair after stroke through FTO-dependent m^6^A methylation. Mechanically, circSCMH1 increased the nuclear translocation of FTO via promoting FTO ubiquitination, leading to the m^6^A demethylation of *Plpp3* mRNA, and thereby induced the increasing of LPP3 level in ECs with subsequent enhancement of vascular repair (Fig. 8). This study sheds light on the functional link between circSCMH1 and m^6^A methylation, indicating m^6^A methylation regulation may be a potential therapeutic strategy for stroke recovery.

Our previous study demonstrated that circSCMH1, in particular, is a potential therapeutic target^17^. The recognition that higher levels of vascular repair correlate with improved functional recovery in animal models and stroke patients represents a therapeutic option for ischemic stroke^4,6^, which led us to further dissect the role of circSCMH1 involved in vascular repair after ischemic stroke. In the current study, microvascular length, area, and branch numbers were increased by EV-circSCMH1 administration in the peri-infarct cortical areas of monkeys or mice after PT stroke. Moreover, EV-circSCMH1 administration enhanced revascularization as well as cerebrovascular integrity. Together, our results indicate that circSCMH1 facilitates vascular repair during the convalescence of ischemic stroke.

M^6^A is a reversible modification mediated by methylases and demethylases in mRNA, and it participates in the regulation of mRNA processing, translation, and stability^18,19^. Consistent with other reports^23–25^, our results showed that total m^6^A was significantly increased after ischemic stroke, suggesting nucleus methylase downregulation or demethylase upregulation. FTO belongs to the α-ketoglutarate-dependent dioxygenase ALKB family of proteins^36^ and participates in the regulation of vascular repair in heart failure and oculopathy^36–38^. Consistent with the previous report^23^, our study indicated that decreased expression of FTO and increased levels of m^6^A were observed in ECs or the peri-infarct cortex after ischemic stroke. Furthermore, circSCMH1 ameliorated the increased m^6^A methylation of the peri-infarct cortex or ECs, but did not attenuate the decreased expression of FTO, implying that circSCMH1 affects m^6^A methylation through other molecular mechanisms rather than regulation of FTO expression. Previous studies indicated the shuttling of FTO in the nucleus and cytoplasm^39^. We evidenced that circSCMH1 interacted with FTO and increased its translocation into the nucleus, alleviating the increased level of m^6^A methylation after ischemic stroke.

Increasing evidence indicates that ubiquitination affects the translocation of FTO into the nucleus^30^. Ubiquitination is covalently coupled to lysine residues on target proteins by a cascade of enzymatic reactions carried out by activating (E1), conjugating (E2), and ligating (E3) enzymes^40^. The cellular functions of ubiquitination span a wide spectrum^41^. Previous studies have demonstrated that the Ub-K63 regulated protein localization^42^. In our study, circSCMH1 was shown to increase the Ub-K63 of FTO. Moreover, UBC13 is a ubiquitin E2 conjugating enzyme that specifically catalyzes the form of Ub-K63 chains^31^. Our study demonstrated that circSCMH1 functioned as a scaffold to enhance the interaction of FTO and UBC13 with subsequently increased Ub-K63 modification of FTO. This molecular mechanism of circSCMH1-mediated Ub-K63 is analogous with other reports that circNDUFB2 facilitates the ubiquitination of IGF2BPs by enhancing the interaction between IGF2BPs and TRIM25 (a kind of E3 ubiquitin-ligating enzyme)^32^.

During vascular repair, ECs perform highly orchestrated morphogenic events that include basement membrane degradation, EC sprouting and branching, vessel lumen formation, vessel anastomosis and maturation, and mural cell recruitment, resulting in a new vascular network that provides blood to the hypoxic tissue^43–45^. In the current study, endothelial-targeted FTO overexpression was shown to enhance vascular area, total length, and branch numbers as well as BBB integrity, further improving motor functional recovery after PT stroke. Moreover, the Cre recombinase is selectively expressed in ECs of the *Fto*^*flox/flox*^ cKI mice to generate endothelial-targeted FTO overexpression. This model mice were observed with an increased level of vascular repair as indicated by the long-term optical imaging. FTO-regulated vascular repair has been demonstrated in retinal and cardiac blood vessels^37,38^, our findings pushes the frontier of basic understanding of the functional link between FTO and vascular repair in ischemic stroke.

M^6^A methylation affects mRNA stability and regulates multiple bioprocesses^19^. In our study, m^6^A modulation was shown to regulate the stability of *Plpp3* mRNA. *Plpp3* encoded LPP3, a cell-surface enzyme that regulates the bioactivities of lysophosphatidic acid (LPA)^46^. The effect of LPA on vascular repair has been demonstrated in previous studies^47^. LPP3 knockdown was shown to decrease the integrity of the endothelial monolayer and the level of angiogenesis in human aortic ECs^48,49^. Anti-LPP3 antibody inhibited basic fibroblast growth factor (bFGF)- and vascular endothelial growth factor (VEGF)-induced capillary morphogenesis of ECs^50^. In our study, circSCMH1 overexpression attenuated *Plpp3* deficiency by FTO-mediated m^6^A methylation, and promoted vascular repair after stroke. This finding indicated that circSCMH1-mediated m^6^A facilitated vascular repair to improve motor functional recovery, most likely by regulating the m^6^A-modified target *Plpp3* mRNA. Even so, we could not rule out the possibility that other mechanisms may underlie the functions of circSCKH1-mediated m^6^A methylation.

Taken together, the findings of this study reveal the functional link between circSCMH1 and m^6^A methylation: circSCMH1 bound with FTO and increased its translocation into the nucleus by ubiquitination, leading to decreased m^6^A methylation of *Plpp3* mRNA and inhibiting degradation of *Plpp3* in ECs, with subsequent improvement of motor functional behaviors. Our study provided proof-of-concept evidence that circSCMH1 enhances vascular repair via FTO-regulated methylation, indicating that regulation of m^6^A methylation may be viewed as a potential therapeutic intervention for functional recovery after stroke in clinical applications.

## Methods

### Ethics statement

All methods and study materials are available in the article and its Data Supplement or from the corresponding author. All mouse experiments were approved by the Institutional Animal Care and Use Committee at the Medical School of Southeast University, Nanjing, Jiangsu, China (approval ID 20200324001). All monkey experiments were approved by the Institutional Animal Care and Use Committee of the Kunming Institute of Zoology, Chinese Academy of Sciences (Institutional Animal Care and Use Committee No. 18016), Kunming, Yunnan, China. The human experiments were approved by the South-Central University for Nationalities Research Ethics and Safety Committee (approval No. 2021-scuec-034).

### Postmortem brain samples

Postmortem brain samples were purchased from the Chinese Brain Bank Center (CBBC) (Wuhan, China) (http://www.cbbcnet.cn/). The CBBC is jointly managed and operated by Tongji Medical College of Huazhong University of Science and Technology, and the Wuhan Institute of Neuroscience and Neural Engineering of South-Central Minzu University. Frozen frontal cortex samples were dissected from Brodmann areas 4 or 6. Brains were dissected by trained neuroanatomists and stored at –80 °C. The acute ischemic stroke individual died by AIS, whereas nonstroke patients were individuals who died naturally and did not have evidence of stroke, active malignant diseases, or neurological and psychiatric diseases (Supplementary Table 1).

### Mice

An all-male mouse colony was used in our experiments to eliminate the interference of the physiological cycles of female mice, in particular, the production of hormone such as estrogen and progesterone, which might disturb exercise performance by altering carbohydrate, fat, and protein metabolism^51^. Adult male C57BL/6 J mice (7–8 weeks old) were purchased from the GemPharmatech Co., Ltd. (Nanjing, China). Mice diet (SFS9112, Xietong Shengwu) and water were available ad libitum. All animals were group-housed under conditions of constant temperature (approximately 21 ^o^C) and humidity (~50%), and a 14-h light/10-h dark cycle. All animal experiments were approved by the Institutional Animal Care and Use Committee at the Medical School of Southeast University and performed in accordance with the Animal Research: Reporting of In Vivo Experiments (ARRIVE) guidelines. The sample size required for animal research was based on previous experimental results and was similar to the sample size commonly used in the field. The mice were coded and randomly divided into experimental and control groups. Euthanasia was done first by exposing mice to CO_2_ air and then followed by cervical dislocation.

### Generation and identification of *Fto*^*flox*^ cKI mice

The targeting construct for the FTO (Gene ID: 26383) overexpressing mice was generated with the assistance of GemPharmatech Co., Ltd. (Nanjing, China). The Cre-loxP recombination technology was used to generate the *Fto*^*flox*^ cKI mice (Supplementary Fig. 15a). This transgenic line was maintained in the C57/BL6 background. *Fto*^*flox*^ cKI mice were generated by insertion of the *Fto* cDNA into a pCAG-STOP-Kozak-ATG plasmid (downstream of the STOP cassette). The mixture containing Cas9, gRNA (sequence: 5′-CTGAGCCAACAGTGGTAGTA−3′), and donor vector was injected into the oosperm of C57/BL6 mice by microinjection. *Fto*^*flox*^ cKI mice possessed loxP sites flanking the STOP cassette. The mouse lines containing the *Fto*^*flox*^ transgene were identified by PCR with three pairs of primers (Supplementary Fig. 15b). The sequences of three pairs (5′arms, 3′arms, and WT) of primers were listed in Supplementary Table 6. *Fto*^*flox*^ cKI mice were homozygous for the respective alleles. To produce endothelial-specific overexpression, Cre recombinase was specifically overexpressed in endothelial cells used by the adeno-associated virus serotype BR1 (i.v.) (5 × 10^11^ v.g for each mouse). Further details are available on request.

### Photothrombotic (PT) stroke mouse model

As previously described in ref. ^17^, cortical microvascular photothrombosis induced focal cortical ischemia in mice. Briefly, anesthesia was induced with 3% isoflurane (R510-22, RWD). The face mask and 1.5% isoflurane were used to maintain the anesthesia. Anesthetized mice were fixed in a stereotaxic device. The skull was exposed via a midline incision of the skin, and the surface was kept clean and dry. Mice were treated with Rose Bengal (30 mg/kg, 330,000, Sigma) by tail vein injection. A cold light source (World Precision Instruments, USA) was positioned 1.5 mm lateral from the bregma. Five minutes after Rose Bengal, the brain was illuminated for 5 min by a cold light source (12,000 lux) attached to an opaque template with a 2 mm diameter. With light stimulation, Rose Bengal occluded blood vessels, resulting in a focal cortical stroke. Sham mice received the same dose of Rose Bengal without illumination. The rectal temperature of mice was controlled at 37.0 ± 0.5 °C with a thermostatic blanket during surgery.

### Transient middle cerebral artery occlusion (tMCAO)

The tMCAO was performed according to the previous study^52^. Mice were induced anesthesia with 3% isoflurane, and the anesthesia was maintained with 1.5% isoflurane using a face mask. The right external carotid artery was exposed, a silicone rubber-coated 6-0 nylon filament (602356PK5Re, Doccol, USA) was inserted and pushed 9–10 mm along the internal carotid artery into the origin of the middle cerebral artery. One hour after the occlusion, the filament was removed to restore blood flow in the central cerebral artery region. During the surgical and recovery periods, the body temperature of mice was maintained at 37.0 ± 0.5 °C using a thermostatic blanket.

### Distal middle cerebral artery occlusion (dMCAO)

As previously described in ref. ^53^, direct occlusion of the distal middle cerebral artery (MCA) induced focal cerebral ischemia. Anesthesia was induced with 3% isoflurane. The face mask and 1.5% isoflurane were used to maintain the anesthesia. Erythromycin eye ointment (H44023089, Baiyunshan, China) was used to prevent eye dryness. Under a dissection microscope (Amscope, California, USA), a vertical skin incision (0.5 cm) was made between the right eye and ear, and the temporal muscle was cut and separated to expose the right lateral aspect of the skull. The MCA was identified through the semi-translucent skull, which was thinned out using a microdrill. The artery was coagulated with the electrocoagulation forceps proximal for 30 s. The temporal muscle was relocated to its original position. Next, the wound was sutured and the animal was placed in a nursing box at 37.0 ± 0.5 °C to recover from the anesthesia.

### Nonhuman primates

All experiments were carried out according to the previous report^17^. Eight male rhesus monkeys (Macaca mulatta, 5.55–9.40 kg, 5–9 years in age) were housed in adjoining individual primate cages, and the animal house was controlled for humidity (~60%), temperature (~21 ^o^C), and light (12 h/12 h: daylight/night cycle) at the Kunming Institute of Zoology (Kunming, China). The residential area is maintained with a 12-h light/dark cycle, with all experiments conducted between 9:00–18:00. All samples were naive monkeys without any history of experimentation. No statistical methods were used to pre-determine the sample size. Researchers and animal care staff monitored the monkeys daily to ensure their health and welfare. A commercial primate diet and fresh fruits and vegetables were provided daily, and water was provided through an automatic watering system in each cage. In the current experiment, intramuscular injection of hydrochloric acidulated ketamine (10 mg/kg) was used to induce anesthesia and sodium pentobarbital (25 mg/kg) was used to maintain anesthesia. Under deep anesthesia, the monkeys were perfused with 4% paraformaldehyde in 1×PBS at PH 7.4. All efforts were made to minimize the pain and discomfort of the animals throughout the course of the study.

### PT stroke model in nonhuman primate

The PT surgery in nonhuman primates was performed according to the previous report^17^. Briefly, monkeys were anesthetized and fixed on the operating table. Their body temperature was maintained using a heating pad. An average of 3.7 cm × 2.8 cm oval-shaped piece of the skull over the sylvian fissure was removed and a cross incision was made at the cerebral dura mater to expose the sylvian fissure and the central sulcus. After 6 min of intravenous injection of Rose Bengal (20 mg/kg), three sites were sequentially exposed to the photoirradiation (532 nm, 1.4 × 10^6^ lux, light spot diameter with 8 mm). The sites are as follows: the first site is the sylvian fissure zone where the M3 segment of the MCA is located. The second and third sites are the adjacent central anterior gurus and central posterior gurus. Gentamicin sulfate (320 U/mL) was used to clean the wound area. The animals were returned to cages for observation until they recovered from anesthesia. Antibiotics was administrated for 3–7 consecutive days to prevent infection. After the surgery, the monkeys were continuously monitored twice a day and provided soft food and fruit when necessary. EV-Vector and EV-circSCMH1 (3 mg/kg) were administered intravenously to the vein of the hind limb 24 and 48 h after the PT surgery. The dosage of EVs was calculated based on the EVs injected in mice (12 mg/kg) adjusted for body surface area according to FDA guidance. (https://www.fda.gov/regulatory-information/search-fda-guidance-documents/estimating-maximum-safe-starting-dose-initial-clinical-trials-therapeutics-adult-healthy-volunteers).

### EV isolation and purification

The EV isolation and purification method was described in our previous study^17^. 293 T cells (SCSP-502) were kindly provided by Cell Bank/Stem Cell Bank, Chinese Academy of Sciences. Briefly, 293 T cells were seeded in 225 cm^2^ flasks (431082, Corning) and cultivated in 5% CO_2_. When reached ~70% confluence, the cells were co-transfected with the vector or circSCMH1 plasmid and RVG-lamp2b (71294, ADDGENE) using Lipofectamine 2000 (11668019, Invitrogen). After 48 h, hygromycin B (10843555001, Roche) and puromycin (AK058, GPC Biotechnology) were added to the cell culture medium to obtain stable strains. EVs were collected from cell culture supernatants by ultracentrifugation. The conditional medium was centrifuged at 300 × *g* for 10 min, 3000 × *g* for 15 min, and 10,000 × *g* for 60 min at 4 °C to remove cells and debris. The supernatants were filtered with 0.22-μm pore filters (SLGPR33RB, Millipore) and centrifuged for 90 min at 200,000 × *g* at 4 °C using an XPNPsi0 ultracentrifuge (Optima XPN-100, Beckman Coulter, USA). The pellet was resuspended with phosphate-buffered saline (PBS) for further study.

### Intravenous delivery of circSCMH1 in animals

EV-circSCMH1 or EV-Vector were administered intravenously in monkeys (3 mg/kg) and mice (12 mg/kg). We divided the monkeys into two groups: PT + EV-Vector and PT + EV-circSCMH1; and we divided the mice into the following groups: Sham, PT + EV-Vector and PT + EV-circSCMH1.

### Mouse behavioral tests

All behavioral tests were carried out according to the previous protocols^17^. Mouse behavioral tests were performed by an independent investigator who was blind to the experimental groups, and the data were analyzed by separate investigators.

The elevated grid area of 32 cm × 20 cm × 50 cm (length × width × height) made of 12 mm square wire mesh was used for the grid-walking task. Each mouse was placed individually on the wire grid and can move freely for a minimum of 100 steps. A foot fault is counted (1) if a step was not able to provide support and the foot went through the grid hole or (2) if the mouse was resting with the grid at the level of its wrist. The number of foot faults and non-faults for each limb were counted. A ratio was calculated as follows: number of foot faults/(number of foot faults + number of non-faults) × 100%.

The cylinder test was used to monitor the use of forelimbs for wall exploration/press in a cylinder. Mice were placed inside a plastic cylinder (15 cm tall with a diameter of 10 cm) and videotaped for 5 min. The exploration/press score was calculated as follows: (number of right hand − number of left hand)/(number of right hand + number of left hand + number of both hands).

For the adhesive removal somatosensory test, two small pieces of adhesive-backed paper dots (of equal size, 25 mm^2^) were used as bilateral tactile stimuli occupying the distal-radial region of the wrist of each forelimb. Time for removing each stimulus from the forelimb was recorded. Before surgery, animals were trained for 3 days. The result was calculated as follows (time in seconds): time of left wrist-time of the right wrist.

### In vivo two-photon laser scanning microscopy

This operation was carried out following our previous protocol^54^. Mice were anesthetized and fixed on a custom-made head holder. The craniotomy was performed over the right cortex using a high-speed microdrill. A window was created and contained the infarction and peri-infarct area of the cortex. To analyze microvascular perfusion, FITC-dextran of 2,000,000 Da (10 mg/mL, FD2000S, Sigma-Aldrich) was injected via the tail vein. Z-stack images were acquired from 100 to 150 μm below the surface of the cortex. The images were scanned and reconstructed using ZEN2011 Imaging Software (Zeiss). The perfused capillary length was analyzed by Angiotool (64 0.6a) software.

### Long-term optical imaging

The custom-built multimodal optical imaging platform and its protocol have been reported in the previous study^55^. Briefly, animals were anesthetized and fixed on a custom-made head holder. The cortical bone was thinned using a dental drill and carefully removed, leaving the dura intact. The region was immediately covered by a 3 × 3 mm^2^sterile coverslip and sealed with biocompatible glue. Images were captured and analyzed using the multimodal optical imaging platform, which includes multi-wavelength (MW) spectral and fluorescence imaging and laser speckle contrast imaging (LSI) modules (MW-LSI). In MW-LSI, the high-brightness light-emitting diodes (LEDs) at the wavelength of λ = 570 nm was used for total hemoglobin (HBT) imaging. The back-reflected light from this channel was collected through a modified zoom microscope (AZ100, Nikon) and acquired by a 16-bit sCMOS camera (Zela 4.2; Andor).

### Flow cytometry analysis and cell sorting

The peri-infarct tissues of mice were collected and then temporarily placed on ice. Tissues were dissociated and digested for 1 h at 37 °C by Papain (2 mg/mL, LS003119, Worthington) in RPMI 1640 medium (C11875500BT, Gibico). The mixture was passed through a 70-μm nylon mesh. Dispersed cells were collected by centrifugation with 300 × *g* for 10 min. The cell pellet was resuspended in 30% Percoll density gradient (17089109, Cytiva) and centrifuged at 900 × *g* for 25 min. Samples were blocked with FcR Blocking Reagent (130-092-575, Miltenyi Biotec) and resuspended in PBS containing 2% FBS. The different cells were assayed for surface antigens by flow cytometry as previously described^17^. Cells were used for fluorescence acquisition and stained with APC anti-Mouse NCAM-1/CD56 Allophycocyanin MAb (FAB7820A-100, R&D), PE anti-mouse ACSA-2 (130-116-244, Miltenyi Biotec), FITC anti-mouse/human CD11b Antibody (101205, BioLegend), PerCp-CyTM5.5 anti-mouse CD45 Antibody (561869, BD Pharmingen), and Brilliant Violet 605™ anti-mouse CD31 (102427, BioLegend). Gating was determined based on the appropriate negative isotype-stained controls. Flow cytometry (BD Biosciences, FACSAria II SORP) was used for fluorescence acquisition, and data were analyzed using FlowJo-V10 software.

### Cell cultures

The mouse brain endothelial cell line-bEnd.3 was purchased from ATCC (CRL-2299, RRID: CVCL-0170) and cultured in 5% CO_2_ at 37 °C in DMEM (30-2002, ATCC) supplemented with 10% (v/v) FBS (10099-141, Gibico), penicillin (100 U/mL), and streptomycin (100 U/mL) (10378-016, Invitrogen). The mouse brain endothelial bEnd.3 cells were used in experiments after 4–10 passages.

Primary human brain microvascular endothelial cells were purchased from iCell Bioscience (HUM-icell-n001, iCell Bioscience) and cultured in 5% CO_2_ at 37 °C in primary endothelial cell basal medium (PriMed-iCell-002, iCell Bioscience). The primary human microvascular endothelial cells were used in experiments after 5–10 passages.

Primary mouse brain microvascular endothelial cells of the cerebral cortex were obtained from C57BL/6 J mice from 8 weeks. Briefly, the mice were sterilized in 75% ethanol for 3 min. The mouse brain was removed under aseptic conditions and placed in a DMEM medium. The cortex was isolated and its apparent blood vessels were removed. The tissues were dissected and incubated in type II collagenase (C6885, Sigma) and DNase solution (18047019, Invitrogen) at 37 °C. After enzymatic hydrolysis for 1 h, the mixture was centrifuged at 4 °C, 1000 × *g* for 10 min. Next, the precipitate was resuspended in sterile 25% bovine serum albumin (BSA, 4240GR100, BioFroxx) and then centrifuged at 4 °C, 1000 × *g* for 20 min. The precipitate was further digested by the collagenase/dispersing enzyme and DNase at 37 °C for 1 h. The endothelial cells were collected in culture medium ECM (1001, ScienCell) and maintained at 37 °C in humidified air containing 5% CO_2_. The medium was changed every 3 days.

### CircSCMH1 construct generation

Detailed information regarding the circSCMH1 overexpression plasmid can be found in our previous study^17^. In short, a basic sequence was synthesized, which contained the 3′ half-intron-exon-II fragment (splice acceptor) of the bacteriophage T4 td gene, a small space sequence, and an exon-I splice donor 5′ half-intron segment. The basic sequence, driven by a CMV promoter, was cloned into the multiple cloning sites of pEGFP-N1 to generate circSCMH1 by replacing the small space sequence. The circSCMH1 sequences was listed in Supplementary Table 2 in the Supplement.

### Plasmid, siRNA, and lentivirus transfection

Primary mouse brain microvascular endothelial cells or bEnd.3 cells of 2 × 10^5^ were transfected with 50 nM siRNA, 1 μg circSCMH1 plasmid, and 1 μL lipofectamine 2000 in 500 μL DMEM for 24 h. For cotransfection of lentivirus and shRNA or siRNA, bEnd.3 cells of 2 × 10^5^ were transfected with the vector and circSCMH1 lentivirus (10^9^ TU/mL) at a multiplicity of infection of 5 in 500 μL DMEM for 24 h. Next, the bEnd.3 cells were transfected with 1 μg shRNA or 50 nM siRNA and 1 μL lipofectamine 2000 in 500 μL DMEM for 24 h. The oligomers for shFTO and shLPP3 were purchased from Genechem Company. The oligomers for siUBC13, siYTHDF2 for mouse, siYTHDF2 for human, and si-circSCMH1 were purchased from GenePharma Company. The sequences of oligomers were listed in Supplementary Table 3.

### Adeno-associated virus (AAV) and lentivirus (LV) injection

AAV-BR1-TIE-Con (AAV-BR1-Con), AAV-BR1-TIE-FTO (AAV-BR1-FTO), AAV-BR1-TIE-Cre (AAV-BR1-Cre), and AAV-BR1-TIE-Plpp3 (AAV-BR1-Plpp3) (AAV-BR1 serotype) were constructed and packaged by Genechem Company. The AAV-BR1-Con, AAV-BR1-FTO, AAV-BR1-Cre, and AAV-BR1-Plpp3 were injected via the tail vein to infect cerebrovascular endothelial cells (5 × 10^11^ v.g for each mouse). The sequences were listed in Supplementary 3in the Supplement.

The LV-si-circSCMH1 were constructed and packaged by Genechem Company. The stereotaxic injection site (from bregma) was as follows: anteroposterior, 0 mm; mediolateral, 1.5 mm; dorsoventral, 1.3 mm. After injection, the needle was left in place for 10 min to ensure well distribution of the lentivirus.

### Microvessel isolation

Brain microvessels were isolated as described previously^56^. Animals under anesthesia were perfused with PBS. The brains were removed and immediately immersed in ice-cold isolation buffer A (103 mM NaCl, 4.7 mM KCl, 2.5 mM CaCl_2_, 1.2 mM KH_2_PO_4_, 1.2 mM MgSO_4_, and 15 mM HEPES [N-2-hydroxyethylpiperazine-*N*′−2-ethanesulfonic acid], pH 7.4, with protease inhibitor). The choroid plexus, meninges, cerebellum, and brain stem were removed, followed by homogenization of the brain in 5 mL of isolation buffer B (Buffer A with 25 mM NaHCO_3_, 10 mM glucose, 1 mM Na pyruvate, and dextran [molecular weight 64 kD; 10 g/L], pH 7.4) with protease inhibitor. Dextran (26%) was then added to the homogenates, followed by centrifugation at 5800 × *g* for 20 min. Pellets were resuspended in isolation buffer B and filtered through a 70-μm mesh filter (352350, Corning). Filtered homogenates were repelleted by centrifugation and smeared on slides.

### Evans blue extravasation assay

As described in our previous study^11^, we evaluated cerebrovascular integrity in C57BL/6 J mice. To assess cerebrovascular permeability, we injected a 2 % Evans blue solution (4 mL/kg) into the tail vein. After 4 h, the mice were anesthetized and perfused with ice-cold PBS (pH 7.4) to remove the intro-vascular Evans blue. The homogenates were precipitated in 15% trichloroacetic acid (1:1 v/v) and centrifuged at 1000 × *g* for 10 min. The pH was adjusted by adding 5 M sodium hydroxide. Evans blue concentrations were spectrophotometrically measured at 620 nm.

For staining images of Evans blue, the brain tissues were cut into 30-μm coronal slices and subsequently incubated with 0.3% Triton X-100 in PBS for 15 min and blocked with 10% normal goat serum (ZLI-9056, ZSGB-BIO) in 0.3% Triton X-100 for 1 h at room temperature. The sections were incubated with biotinylated- Isolectin B_4_ (Ib4, L2140, Sigma-Aldrich) overnight and then streptavidin-FITC secondary antibody for 1 h.

### Oxygen-glucose deprivation (OGD) treatment

OGD treatment was performed as previously described in ref. ^17^. Briefly, cells were cultured with DMEM without glucose (11966-025, Gibco) in an incubator (Thermo Scientific) with premixed gas (95% N_2_and 5% CO_2_) for 12 h. The control group was cultured with normal DMEM in 5% CO_2_ for the same incubation time.

### Immunostaining and image analysis

Brain tissues were cut into 30-μm coronal slices and subsequently incubated with 0.3% Triton X-100 in PBS for 15 min and blocked with 10% normal goat serum (ZLI-9056, ZSGB-BIO) in 0.3% Triton X-100 for 1 h at room temperature. The sections were incubated with primary antibodies (Supplementary Table 7) overnight. Sections were then incubated with Alexa Fluor goat anti-rabbit IgG or Alexa Fluor goat anti-mouse IgG for 1 h at room temperature. The sections were mounted onto glass slides after being washed with PBS. Images were captured by confocal microscopy (FV3000, Olympus, Japan) with the transmission electron microscope (H-7650, HITACHI, Japan) and analyzed by Image J (1.53k) and Angiotool (64 0.6a) software.

### Examination of in vivo endothelial cell proliferation by BrdU labeling

The newly proliferated cells were labeled with 5-bromo-2′-deoxyuridine (BrdU, B5002, Sigma-Aldrich) as previously described^57^. Briefly, BrdU (50 mg/kg) was injected intraperitoneally once per day on days 3–10 after PT. The brain sections were prepared as described above. Sections were pretreated in 1 N HCl for 30 min, followed by 0.1 M boric acid (pH 8.5) for 10 min at room temperature. Sections were subsequently incubated with 0.3% Triton X-100 in PBS for 15 min and blocked with 10% normal goat serum (ZLI-9056, ZSGB-BIO) in 0.3% Triton X-100 for 1 h at room temperature. The mouse anti-BrdU antibody (sc-51514, Santa Cruz) was added to sections in buffer and incubated overnight at 4 °C. After a series of washes with PBS, sections were incubated with Alexa Fluor goat anti-rabbit IgG or Alexa Fluor goat anti-mouse IgG for 1 h at room temperature. The sections were mounted onto glass slides after being washed with PBS. Images were captured by confocal microscopy (FV3000, Olympus, Japan) with a transmission electron microscope (H-7650, HITACHI, Japan) and analyzed by Image J (1.53k) software.

### RNA-binding protein immunoprecipitation (RIP)

Cultured bEnd.3 cells or primary mouse brain microvascular endothelial cells were harvested with 0.5 mL lysis buffer according to the manufacturer’s protocol of Magna RIP RNA-binding protein immunoprecipitation kit (17–700, Millipore). The magnetic beads were washed with RIP washing buffer. Next, antibodies were added to the beads in RIP washing buffer and incubated overnight for 30 min at room temperature. Next, cell lysates were incubated with the antibody-bound beads in RIP immunoprecipitation buffer overnight at 4 °C. RIP immunoprecipitation buffer was centrifuged for 5 min at 13,523 × *g* to remove the supernatant and the pellet was resuspended in the immuno-precipitated buffer containing in proteinase K and incubated at 55 °C for 30 min with shaking to digest the protein. Purified RNAs were examined for the expression of circSCMH1 via quantitative polymerase chain reaction (qPCR).

### Measurement of total m^6^A

Total m^6^A levels of total RNA were measured using an m^6^A RNA methylation quantification kit (P-9005; EpiGentek, Farmingdale, NY) according to the manufacturer’s instructions. In brief, 200 ng RNA was bound to strip wells using RNA high-binding solution. M^6^A was enhanced and detected using capture and detection antibodies. The detected signal of m^6^A is enhanced and then quantified calorimetrically by reading the absorbance in a microplate spectrophotometer at 450 nm.

In order to calculate intra-assay variability, we applied the m^6^A detection kit to detect the same sample ten times in one independent test, and the level of m^6^A in total RNA was converted according to the calculation formula. As for inter-assay variability, the level of m^6^A of the same sample was subjected to six independent trials. The intra-assay and inter-assay variability were calculated as 6.80% and 9.16%, respectively, by dividing the standard deviation of a set of measurements by the set mean, and multiplying by 100.

### Methylated RNA immunoprecipitation

MeRIP assay was conducted with an *N*^6^-Methylated RNA Immunoprecipitation (MeRIP) Kit (Bes5203, BersinBio, China) according to the guidelines. In brief, total RNA was fragmented to approximately 300 nt by fragmentation buffer at 94 ^o^C for 2 min, and ethylenediamine tetraacetic acid (EDTA) was added to stop the reaction. One-ninth of the fragmented RNA was saved as an input control, and the rest of the fragmented RNA was applied to immunoprecipitation with a 5 μg m^6^A antibody at 4 ^o^C overnight. The prepared Magnetic Beads of Protein A/G were incubated with IP mix buffer at 4 ^o^C for 3 h, then digested the beads with Proteinase K at 55 ^o^C for 45 min. The IP fragmented RNA was extracted with Phenol-Chloroform-Isoamylol. The m^6^A status of *Plpp3* was further measured by real-time PCR using m^6^A peak primers in Supplementary Table 7.

### MeRIP sequencing

Total RNAs from peri-infarct tissues of PT mice treated with EV-Vector or EV-circSCMH1 (*n* = 12 each group) were extracted and decomposed into 100–200 nt-sized fragments. The fragmented polyadenylated RNA was incubated with anti-m^6^A antibody and dynabeads protein in 1 × IP buffer (10 mM Tris-HCl, pH 7.4, 150 mM NaCl, 0.1% NP-40). After being washed three times with 1 × IP buffer, all samples were enriched by m^6^A immunoprecipitation and then subjected to single-end sequencing on Illumina HiSeq 4000 by Aksomics. The Gene Expression Omnibus (GEO) accession code for the data in this manuscript number is GSE193633. The detailed m^6^A peaks were shown in Supplementary Tables 4, 5.

### RNA pull-down assays

RNA pull-down assays were performed as previously described^17^. In brief, 10^7^ cells were washed in ice-cold phosphate-buffered saline buffer and lysed in 500 μL co-IP buffer (P0013F, Beyotime) with a mixture of proteinase inhibitors and phosphatase inhibitors. The sample was incubated with a 6 μg biotin-labeled circSCMH1 probe against endogenous or ectopically expressed circSCMH1 at room temperature for 2 h. The mouse circSCMH1 probe sequence, which was biotinylated at the 5′-end, was 5′-aaaTTGGAGGTGTGTAGGACTTTGGTGCCAGGTGG-3′, which was synthesized by Invitrogen. Lysate solution was further mixed with streptavidin C1 magnetic beads (65002, Invitrogen) for 1 h at room temperature. The co-IP buffer was used to wash the beads five times. Western blot was used to analyze the binding proteins in the pull-down materials.

### Co-immunoprecipitation (Co-IP)

Co-IP was performed to detect the protein–protein interactions and the level of ubiquitination. Cells were washed in ice-cold phosphate-buffered saline buffer and lysed in 500 μL co-IP buffer (P0013F, Beyotime) with a mixture of proteinase inhibitors and phosphatase inhibitors. The lysates were incubated with 5 μg antibodies. After incubation at 4 °C overnight, a total of 50 μL washed magnetic beads (sc-2003, Santa) were added, and the lysates were further incubated at 4 °C overnight. The beads were washed five times with co-IP buffer. The bound proteins in the pull-down materials were analyzed by western blot.

### qPCR

The qPCR was performed according to our previous study in an Applied Biosystems qPCR System^17,58^. TRIzol reagent (15596026, Invitrogen) was used to extract total RNA. The cDNA was acquired using the HiScript QRT SuperMix for qPCR Kit (R123-01, Vazyme). CircRNAs and mRNAs were quantified using SYBR Green qPCR Master Mix (Q141-02, Vazyme) and calibrated by internal control GAPDH. The cycle threshold was detected by StepOne^TM^ Real-Time PCR instrument (StepOne^TM^ 4480845, applied biosystems®). The primers were synthesized by Invitrogen and listed in Supplementary Table 6.

### RNA stability

The bEnd.3 cells were co-transfected with circSCMH1 and FTO shRNA or siYTHDF2. The cells were treated with 5 μg/mL actinomycin D (A9415, Sigma) and harvested at 0, 3, and 6 h. Total RNA was extracted, and *Plpp3* mRNA was measured by qPCR.

### Preparation of protein extract and western blot analysis

As previously described in ref. ^17^, nuclear and total protein extracts were prepared using the nuclear and plasma separation kit (P0028, Beyotime) and the RIPA lysis buffer (P0013B, Beyotime), respectively. Protein extract was separated onto sodium dodecyl sulfate-polyacrylamide gels and electrophoretically transferred onto polyvinylidene fluoride membranes. The membranes were cultivated by specific primary antibodies overnight at 4 °C and then incubated with horseradish peroxidase-conjugated goat anti-mouse/rabbit IgG secondary antibodies (1:2000, SA00001/SA00001-2, Proteintech). Signals were detected by a Tanon (Tanon 5200, Shanghai) digital image scanner and quantified using Image J software. The information of the primary antibody was listed in Supplementary Table 7.

### Statistics

All data for continuous variables are presented as mean ± SEM or median (interquartile range). Significance was assessed with the Student *t*-test (two-sided) for comparisons of two groups. One-way ANOVA followed by the Holm–Sidak test and two-way analysis of variance followed by Bonferroni’s post hoc multiple comparison tests were used for comparisons of ≥3 groups. Behavioral data collected at multiple, sequential time points (i.e., for a single animal at six-time points) were analyzed using two-way repeated-measures ANOVA, followed by the Holm–Sidak post hoc test. The statistical analyses used for different experiments are described in respective figure legends, and results were statistically significant if *P* < 0.05. Statistical analysis was performed using GraphPad Prism 8.0.1 Software.

### Reporting summary

Further information on research design is available in the Nature Portfolio Reporting Summarylinked to this article.

## Supplementary information

Supplementary Information

Reporting Summary

## Data Availability

The MeRIP-sequencing data in this study have been deposited in the Gene Expression Omnibus database under accession code GSE193633. All the data supporting this study are available within the article, the Supplementary file, and the Source data file, as indicated in the Reporting summary for this article. Source dataare provided with this paper.

## Source data

Source Data
