# Supplementary material for: FTO-dependent m6A modification of Plpp3 in circSCMH1-regulated vascular repair and functional recovery following stroke
Source: Nat Commun. 2023 Jan 30;14:489. doi: 10.1038/s41467-023-36008-y (PMC9886939; doi:10.1038/s41467-023-36008-y)
Supplement: Supplementary file 1 — Supplementary Information [file 41467_2023_36008_MOESM1_ESM.pdf]

Supplementary Information

FTO-Dependent M^{6}A Modification of *Plpp3* in CircSCMH1-Regulated Vascular Repair and Functional Recovery Following Stroke

Bin Li[1,#], Wen Xi[1,#], Ying Bai[1], Xue Liu[1], Yuan Zhang[1], Lu Li[1], Liang Bian[1], Chenchen Liu[1], Ying Tang[1], Ling Shen[1], Li Yang[1], Xiaochun Gu[2], Jian Xie[1], Zhongqiu Zhou[1], Yu Wang[1], Xiaoyu Yu[1], Jianhong Wang[3], Jie Chao[4*], Bing Han[1*], and Honghong Yao[1,5,6*]

[1] Department of Pharmacology, Jiangsu Provincial Key Laboratory of Critical Care Medicine, School of Medicine, Southeast University, Nanjing, Jiangsu, China

[2] Jiangsu Key Laboratory of Molecular and Functional Imaging, Department of Radiology, Zhongda Hospital, Medical School of Southeast University, Nanjing, Jiangsu, China

[3] National Research Facility for Phenotypic and Genetic Analysis of Model Animals, Kunming Institute of Zoology, Chinese Academy of Sciences, Kunming, Yunnan, China

[4] Department of Physiology, Jiangsu Provincial Key Laboratory of Critical Care Medicine, School of Medicine, Southeast University, Nanjing, Jiangsu, China

[5] Co-innovation Center of Neuroregeneration, Nantong University, Nantong, Jiangsu, China

[6] Institute of Life Sciences, Key Laboratory of Developmental Genes and Human Disease, Southeast University, Nanjing, Jiangsu, China

[#] These authors contributed equally: Bin Li, Wen Xi.

Addresses for Correspondence:

Honghong Yao, Ph.D
Department of Pharmacology, Jiangsu Provincial Key Laboratory of Critical Care Medicine
Medical School of Southeast University
Nanjing, 210009, Jiangsu, China
Tel: +86 25 8327 2551
E-mail: yaohh@seu.edu.cn

Bing Han, Ph.D
Department of Pharmacology, Jiangsu Provincial Key Laboratory of Critical Care Medicine
Medical School of Southeast University
Nanjing, 210009, Jiangsu, China
Tel: +86 25 8327 2551
E-mail: hanb@seu.edu.cn

Jie Chao, Ph.D
Department of Physiology, Jiangsu Provincial Key Laboratory of Critical Care Medicine
Medical School of Southeast University
Nanjing, 210009, Jiangsu, China
Tel: +86 25 8327 2312
E-mail: chaojie@seu.edu.cn

Supplementary Table 1. The information of postmortem brain samples

	Control (n=6)	AIS (n=6)	*P* value
**Age (years)**	62.33±1.05	62.83±2.39	0.851
**Gender**	5 males/1 female	5 males/1 female	NA
**PMI (h)**	10.8±1.02	9.05±0.30	0.133
**Brain weight (g)**	1300±61.99	1203±42.38	0.226
**Storage time (years)**	1.65±0.40	2.125±0.17	0.304
**Clinical diagnoses**	Toxemia (2)	AIS (6)	NA
	Pneumonia (1)		
	Respiratory failure (1)		
	Hypertension (2)		

Note: AIS, acute ischemic stroke; PMI, post-mortem interval; NA, not applicable; Data were presented as the mean±SEM; the Student *t* test (two-sided).

Supplementary Table 2. The sequences used for plasmid construction.

Name	Sequence
mmu_circ_0001254 (circSCMH1)	TCCTACACACCTCCAAGTAATGAGTTCAAGATCAGCATGAAATTG<br>GAAGCACAGGATCCCAGGAACACCACATCCACCTGTATTGCCACG<br>GTCGTTGGATTGACAGGTGCCCGACTTCGTCTGCGCCTTGATGGC<br>AGTGACAACAAGAATGACTTCTGGAGACTGGTTGACTCCTCTGAA<br>ATCCAGCCAATTGGAAACTGTGAGAAGAATGGCGGGATGCTGCAG<br>CCCCCTCTAGGATTTCGGCTGAATGCCTCCTCTTGGCCCATGTTCC<br>TTTTGAAGACACTAAATGGAGCAGAGATGGCTCCCATCAAGATTT<br>TCCATAAGGAGCCACCATCACCTTCCCACAACTTCTTCAAAATGG<br>GAATGAAGTTAGAAGCTGTAGACAGAAAGAACCCTCATTTCATTT<br>GCCCAGCCACTATTGGAGAAGTTCGAGGCGCAGAAGTGCTAGTCA<br>CCTTTGATGGGTGGCGAGGCGCATTTGACTACTGGTGCCGCTTTG<br>ACTCCCGGGACATCTTTCCTGTGGGCTGGTGTTCTTTGACTGGAG<br>ATAACCTGCAGCCACCTGGCACCAAAG
Rhesus monkey 1:44027532|44047653 (circSCMH1)	TCCTACACACCTCCAAGCAACGAATTCAAGATCAGTATGAAATTG<br>GAAGCACAGGACCCCAGGAACACCACATCCACCTGTATTGCCACA<br>GTAGTTGGACTGACAGGAGCCCGCCTTCGCCTGCGCCTTGATGGG<br>AGTGACAACAAAAATGACTTCTGGCGGCTGGTTGACTCAGCTGAA<br>ATTCAGCCTATTGGGAACTGTGAAAAGAATGGGGGTATGCTACAG<br>CCACCTCTTGGATTTCGGCTGAATGCATCTTCTTGGCCCATGTTCCT<br>TTTGAAGACATTAAATGGAGCAGAGATGGCTCCCATCAGGATTTTC<br>CACAAGGAGCCACCATCACCTTCCCACAACTTCTTCAAAATGGGA<br>ATGAAGCTAGAAGCTGTGGACAGGAAGAACCCTCATTTCATTTGC<br>CCAGCCACTATTGGGGAGGTTCGGGGCTCAGAGGTGCTTGTCACT<br>TTTGATGGGTGGCGAGGGGCCTTTGACTACTGGTGCCGCTTCGAC<br>TCCCGGGACATCTTCCCTGTGGGCTGGTGTTCCTTGACTGGAGAC<br>AACCTACAGCCTCCTGGCACCAAAG
Fat mass and obesity associated (FTO)	ATGAAGCGCGTCCAGACCGCGGAGGAACGAGAGCGGGAAGCTAA<br>GAAACTGAGGCTCCTTGAGGAGCTTGAAGACACTTGGCTTCCTTA<br>CCTGACCCCCAAAGATGATGAGTTCTATCAGCAGTGGCAGCTGAA<br>ATACCCTAAACTGGTTTTCCGAGAGGCCGGCAGCATACCAGAGGA<br>GCTGCATAAGGAGGTCCCCGAGGCCTTTCTCACACTGCATAAGCA<br>TGGCTGCTTGTTTCGGGACGTGGTGAGGATCCAAGGCAAAGATGT<br>GCTCACCCCAGTGTCTCGCATCCTCATCGGGGACCCAGGCTGCAC<br>CTACAAGTACTTGAACACCAGACTCTTCACGGTGCCCTGGCCCGT<br>GAAGGGCTGCACGGTCAAGTACACAGAGGCTGAGATCGCCGCTG<br>CATGTCAGACCTTCCTAAAGCTCAATGACTACCTCCAGGTGGAGA<br>CCATCCAGGCCTTGGAAGAACTGGCTGTCAGAGAGAAGGCCAAT<br>GAAGACGCTGTGCCACTGTGCATGGCAGAGTTCCCCAGGGCCGG<br>CGTGGGGCCGTCCTGCGATGATGAAGTGGACCTTAAGAGCAGAGC<br>AGCCTACAACGTGACTTTGCTAAACTTCATGGATCCTCAGAAGAT<br>GCCCTACTTGAAAGAGGAGCCCTATTTCGGCATGGGGAAGATGGC<br>GGTGAGCTGGCATCACGATGAGAACCTGGTGGACAGGTCAGCCG

	TGGCAGTGTACAGCTATAGCTGCGAAGGCTCTGAGGATGAAAGTG<br>AGGACGAGTCCAGCTTCGAAGGCAGAGATCCTGATACTTGGCATG<br>TTGGTTTTAAGATCTCTTGGGACATCGAGACACCAGGATTAACAAT<br>CCCTCTTCACCAGGGAGACTGCTATTTCATGCTGGATGACCTCAAT<br>GCCACCCACCAGCACTGTGTTTTGGCTGGCTCACAGCCTCGGTTT<br>AGTTCCACTCACCGTGTGGCAGAGTGCTCAACAGGCACCTTGGAT<br>TATATCTTAGAACGCTGTCAGTTGGCGCTGCAGAATGTCCTCAATG<br>ACTCAGACGATGGCGACGTCTCGTTGAAATCCTTTGATCCTGCAGT<br>TTTGAAACAAGGAGAGGAAATCCATAATGAGGTGGAGTTTGAGTG<br>GCTGAGGCAGTTCTGGTTTCAAGGCAATCGATACAAACTTTGCAC<br>CGATTGGTGGTGTGAGCCCATGACTCACCTGGAGGGGCTGTGGAA<br>GAAGATGGAGAGCATGACAAATGCGGTGCTCCGTGAAGTTAAAA<br>GAGAGGGGCTCCCGGTGGAACAAAGGAGTGAGATTCTGTCTGCC<br>ATCCTGGTCCCGCTCACCGTGCGCCAGAACCTGAGGAAGGAGTG<br>GCATGCCAGGTGCCAGTCCCGAGTCGTCCGGACTTTACCAGTACA<br>GCAGAAACCAGACTGCCGGCCATATTGGGAGAAGGATGACCCTTC<br>CATGCCTCTGCCCTTTGACCTCACAGACGTGGTTTCCGAGCTCAG<br>AGGCCAGCTGCTGGAAGCAAGATCCGGCAGCGGCGAGGGCAGAG<br>GAAGTCTTCTAACATGCGGTGACGTGGAGGAGAATCCCGGCCCT
Lipid phosphate phosphatase 3 (*Plpp3*)	ATGCAAAGCTACAAGTACGACAAGGCGATCGTCCCTGAGAGTAAG<br>AACGGCGGCAGCCCGGCGCTCAACAACAACCCGAGGAAGGGCG<br>GCAGCAAGCGGGTGCTGCTCATCTGCCTGGACCTCTTCTGCCTCTT<br>CATGGCGGCTCTGCCCTTCCTCATCATCGAGACAAGCACCATTAAG<br>CCTTACCGTCGAGGGTTTTACTGCAATGACGAGAGCATCAAGTATC<br>CCCTGAAAGTCAGTGAGACTATAAACGATGCTGTGCTCTGTGCGG<br>TGGGGATCGTCATCGCCATCCTGGCGATCATTACAGGGGAATTCTA<br>CCGGATCTATTACCTCAAGGAGAAGTCCCGCTCCACCACTCAGAA<br>CCCGTATGTGGCAGCTCTCTATAAGCAAGTGGGATGCTTCCTTTTC<br>GGCTGTGCCATTAGCCAGTCCTTCACCGACATCGCCAAAGTGTCC<br>ATCGGGCGCCTGAGGCCTCACTTCCTGAGCGTCTGTGACCCTGAT<br>TTCAGTCAGATCAATTGCTCCGAGGGCTACATTCAGAACTACAGG<br>TGCAGAGGAGAAGACAGCAAAGTACAGGAGGCCAGGAAGTCCTT<br>CTTCTCGGGCCACGCCTCCTTCTCCATGTTCACTATGCTGTATCTGG<br>TGCTCTACCTTCAGGCCCGCTTCACCTGGCGCGGGGCCCGACTGC<br>TCCGCCCCCTCCTGCAGTTCACTTTGCTCATGATGGCCTTCTACAC<br>GGGATTGTCACGGGTATCTGACTACAAGCATCATCCTAGCGATGTC<br>CTGGCAGGATTTGCCCAAGGAGCTCTGGTGGCCTGCTGCATAGTG<br>TTCTTCGTGTCCGACCTCTTCAAGACTAAGACGAGCCTCTCACTG<br>CCCGCCCCTGCGATCAGGAGGGAGATCCTGTCTCCCGTGGACATC<br>ATCGACAGGAACAATCACCATAACATGGTGGGCAGCGGCGAGGG<br>CAGAGGAAGTCTTCTAACATGCGGTGACGTGGAGGAGAATCCCG<br>GCCCT
Cyclization recombination	ATGTCCAATTTACTGACCGTACACCAAAATTTGCCTGCATTACCGG<br>TCGATGCAACGAGTGATGAGGTTCGCAAGAACCTGATGGACATGT

(Cre)	TCAGGGATCGCCAGGCGTTTTCTGAGCATACCTGGAAAATGCTTCT<br>GTCCGTTTGCCGGTCGTGGGCGGCATGGTGCAAGTTGAATAACCG<br>GAAATGGTTTCCCGCAGAACCTGAAGATGTTCGCGATTATCTTCTA<br>TATCTTCAGGCGCGCGGTCTGGCAGTAAAAACTATCCAGCAACATT<br>TGGGCCAGCTAAACATGCTTCATCGTCGGTCCGGGCTGCCACGAC<br>CAAGTGACAGCAATGCTGTTTCACTGGTTATGCGGCGGATCCGAA<br>AAGAAAACGTTGATGCCGGTGAACGTGCAAAACAGGCTCTAGCG<br>TTCGAACGCACTGATTTCGACCAGGTTCGTTCACTCATGGAAAATA<br>GCGATCGCTGCCAGGATATACGTAATCTGGCATTTCTGGGGATTGC<br>TTATAACACCCTGTTACGTATAGCCGAAATTGCCAGGATCAGGGTT<br>AAAGATATCTCACGTACTGACGGTGGGAGAATGTTAATCCATATTG<br>GCAGAACGAAAACGCTGGTTAGCACCGCAGGTGTAGAGAAGGCA<br>CTTAGCCTGGGGGTAACTAAACTGGTCGAGCGATGGATTTCCGTCT<br>CTGGTGTAGCTGATGATCCGAATAACTACCTGTTTTGCCGGGTCAG<br>AAAAAATGGTGTTGCCGCGCCATCTGCCACCAGCCAGCTATCAAC<br>TCGCGCCCTGGAAGGGATTTTTGAAGCAACTCATCGATTGATTTAC<br>GGCGCTAAGGATGACTCTGGTCAGAGATACCTGGCCTGGTCTGGA<br>CACAGTGCCCGTGTCGGAGCCGCGCGAGATATGGCCCGCGCTGGA<br>GTTTCAATACCGGAGATCATGCAAGCTGGTGGCTGGACCAATGTA<br>AATATTGTCATGAACTATATCCGTAACCTGGATAGTGAAACAGGGG<br>CAATGGTGCGCCTGCTGGAAGATGGCGATGGCAGCGGCGAGGGC<br>AGAGGAAGTCTTCTAACATGCGGTGACGTGGAGGAGAATCCCGG<br>CCCT

Supplementary Table 3. The sequences of oligomers

Name	Sequence
shFTO (mouse)	Sense: 5'- GCAGCUGAAAUACCCUAAATT -3'
	Antisense: 5'- UUUAGGGUAUUUCAGCUGCTT -3'
shLPP3 (mouse)	Sense: 5'- GCUGCAUAGUGUUCUUCGUTT -3'
	Antisense: 5'- ACGAAGAACACUAUGCAGCTT -3'
siUBC13 (mouse)	Sense: 5'- CGCAGGAUCAUCAAGGAAATT -3'
	Antisense: 5'- UUUCCUUGAUGAUCCUGCGTT -3'
siYTHDF2 (mouse)	Sense: 5'- GCCCUAUCUAACUUCUUAUTT -3'
	Antisense: 5'- AUAAGAAGUUAGAUAGGGCTT -3'
siYTHDF2 (human)	Sense: 5'- CCGUUCCAUUAAGUAUAAUTT -3'
	Antisense: 5'- AUUAUACUUAAUGGAACGGTT -3'
si-circSCMH1 (mouse)	Sense: 5'- ACCUGGCACCAAAGUCCUATT -3'
	Antisense: 5'- UAGGACUUUGGUGCCAGGUTT -3'

Supplementary Table 4. Significantly upregulated m^{6}A modification peaks (PT+EV-Vector vs Sham)

Gene_name	fold_change	P_value	Peak_start	Peak_end	Trans_id	Peak_region
Plpp3	**3.70635**	**1.14815E-07**	**105232584**	**105232764**	**ENSMUST00000064139.7**	**utr3**
Hnrnph1	3.31728	0.0123027	50386437	50386527	ENSMUST00000069304.13	utr3
Hnrnph1	3.24901	0.0141254	50386437	50386528	ENSMUST00001109142.7	utr3
Hnrnph1	3.24901	0.0141254	50386437	50386528	ENSMUST00000077817.7	utr3
Plxnb2	2.92817	0.00549541	89155578	89155728	ENSMUST00000060808.9	utr3
Plxnb2	2.88786	0.0060256	89155578	89155729	ENSMUST00001109331.7	utr3
Akap17b	2.49666	0.030903	36611777	36611987	ENSMUST00000051906.12	cds
Isca2	2.34567	0.0218776	84774996	84775085	ENSMUST00000021667.6	utr3
Grin2b	2.28153	0.0186209	135731082	135731263	ENSMUST00000111905.7	utr3
Acsl3	2.23457	0.0131826	78706874	78706995	ENSMUST00000035779.14	utr3
Grin2b	2.21914	0.0239883	135731081	135731262	ENSMUST00000053880.12	utr3
Zzef1	2.18859	0.00707946	72926877	72927118	ENSMUST00000207107.1	utr3
Fam49a	2.14355	0.00194984	12376268	12376389	ENSMUST00000069066.13	utr3
Zzef1	2.07053	0.00707946	72926849	72927120	ENSMUST00000172220.7	utr3
Zzef1	2.07053	0.00707946	72926849	72927120	ENSMUST00000069395.6	utr3
Gpm6b	2.05623	2.29087E-07	166388898	166388988	ENSMUST00000060210.13	utr3
Gpm6b	2.05623	2.29087E-07	166388898	166388988	ENSMUST00000112233.7	utr3
Mgat5	2.05623	0.0346737	127487856	127488037	ENSMUST00000038361.10	utr3
Tmem35a	2.04202	0.00562341	134304890	134305070	ENSMUST00000037687.7	utr3
Gpm6b	2.02792	3.46737E-07	166388897	166388988	ENSMUST00000112229.8	utr3
Gpm6b	2.02792	3.46737E-07	166388897	166388988	ENSMUST00000112227.8	utr3
Gpm6b	2.02792	3.46737E-07	166388897	166388988	ENSMUST00000112226.2	utr3
Gpm6b	2.02792	3.46737E-07	166388897	166388988	ENSMUST00000112223.7	utr3
Gpm6b	2.02792	3.46737E-07	166388897	166388988	ENSMUST00000112228.7	utr3
Gpm6b	2.02792	3.46737E-07	166388897	166388988	ENSMUST00000112224.7	utr3
Gpm6b	2.02792	3.46737E-07	166388897	166388988	ENSMUST00000112235.7	utr3
Epha5	2	0.0263027	84416608	84416819	ENSMUST00000113401.3	utr5

H13	2	0.00144544	152704782	152705082	ENSMUST00000125366.7	utr3
Luzp2	1.99861	0.0128825	55268167	55268258	ENSMUST00000082373.7	utr3
Arhgap21	1.94801	0.00812831	20847918	20848069	ENSMUST00000154230.8	utr3
Arhgap21	1.94801	0.00812831	20847918	20848068	ENSMUST00000114594.7	utr3
Mfsd2a	1.93187	0.0489779	122946849	122947029	ENSMUST00000030408.11	utr3
Slc2a3	1.90264	0.0301995	122727838	122727959	ENSMUST00000032476.10	utr3
Tdrkh	1.9	0.0371535	94425947	94426158	ENSMUST00000196386.4	utr3
Epha5	1.88557	0.0301995	84416591	84416831	ENSMUST00000053733.14	utr5
Brd9	1.88296	0.0112202	73960773	73960894	ENSMUST00000222399.1	utr3
Tdrkh	1.87125	0.040738	94425948	94426154	ENSMUST00000200486.4	cds, utr3
Brd9	1.86607	0.0123027	73960775	73960895	ENSMUST00000099384.3	utr3
Ptges3	1.84165	0.00512861	128077032	128077272	ENSMUST00000052798.13	utr3
Cyfip2	1.8302	0.00316228	46193854	46193945	ENSMUST00000093166.10	utr3
Cyfip2	1.81001	0.00281838	46193849	46193940	ENSMUST00000165599.8	utr3
Cyfip2	1.81001	0.00281838	46193849	46193940	ENSMUST00000093165.11	utr3
Slc43a2	1.80751	0.0323594	75577031	75577331	ENSMUST00000169547.8	utr3
Slc43a2	1.80751	0.0323594	75577035	75577335	ENSMUST00000042561.13	utr3
Pafah1b1	1.80375	0.0114815	74674008	74674069	ENSMUST00000021091.14	utr3
Apc	1.79502	0.0288403	34276566	34279278	ENSMUST00000115781.9	cds
Dnajc11	1.78757	0.0251189	151981066	151981245	ENSMUST00000062904.10	utr3
Tpm3	1.76418	0.0457088	90086496	90087485	ENSMUST00000127955.1	cds, utr5
Arl8b	1.76296	0.000758578	108823578	108823787	ENSMUST00000032196.8	utr3
Nap1l4	1.76174	0.0446684	143534366	143549104	ENSMUST00000207948.1	utr5
Lmbrd1	1.7593	0.00812831	24762835	24763015	ENSMUST00000095062.9	utr3
Usp10	1.74836	0.0371535	119957375	119957555	ENSMUST00000144458.7	utr3
Ntrk2	1.72907	0.0114815	58932491	58932881	ENSMUST00000224259.1	utr3
H13	1.7219	0.0112202	152704769	152705036	ENSMUST00000089059.8	utr3
Usp10	1.7219	0.040738	119957380	119957560	ENSMUST00000108988.8	utr3
Scn2b	1.70291	0.0229087	45127294	45127444	ENSMUST00000170998.8	utr3

Txndc11	1.66324	0.0380189	11074910	11075179	ENSMUST00000038424.13	utr3, cds
Dhx9	1.66209	0.0229087	153455787	153455998	ENSMUST00000042141.11	utr3
Dhx9	1.66209	0.0229087	153455787	153455996	ENSMUST00000186380.6	utr3
Prrc2c	1.66094	0.00389045	162671934	162672234	ENSMUST00000183223.7	utr3
Slc38a1	1.65176	0.0316228	96571417	96571568	ENSMUST00000100262.2	utr3
Slc38a1	1.65176	0.0316228	96571417	96571568	ENSMUST00000088454.11	utr3
Slc38a1	1.65176	0.0316228	96571417	96571567	ENSMUST00000088452.10	utr3
Prrc2c	1.64947	0.00398107	162671936	162672236	ENSMUST00000182393.7	utr3
Grin2b	1.64832	0.00831764	135713232	135713383	ENSMUST00000053880.12	utr3
Ntrk2	1.64832	0.0194984	58932483	58932872	ENSMUST00000109838.9	utr3
Araf	1.64262	0.0060256	20852680	20852889	ENSMUST00000120356.7	utr3
C1qa	1.64148	0.0199526	136895916	136896095	ENSMUST00000046285.5	utr3
Prrc2c	1.63467	0.0047863	162671924	162672225	ENSMUST00000182660.7	utr3
Grin2a	1.63241	0.0144544	9570746	9571017	ENSMUST00000199708.4	utr3
Lmbrd1	1.63014	0.0141254	24762818	24763028	ENSMUST00000191471.6	utr3
Fgf13	1.62789	0.0223872	59062621	59062920	ENSMUST00000119833.7	utr3
Fgf13	1.62789	0.0229087	59062622	59062921	ENSMUST00000119306.1	utr3
Prrc2c	1.62789	0.00512861	162671922	162672222	ENSMUST00000182149.7	utr3
Brd2	1.62338	0.0398107	34112022	34112202	ENSMUST00000114242.8	utr3
Araf	1.58777	0.00645654	20852665	20852904	ENSMUST00000122312.7	utr3
Dst	1.58227	0.0489779	34174749	34177930	ENSMUST00000183302.5	cds
Brd2	1.58118	0.0489779	34112022	34112203	ENSMUST00000025193.12	utr3
Numa1	1.56917	0.0229087	101999271	101999689	ENSMUST00000209639.1	cds
Fgf13	1.56808	0.0251189	59062620	59062948	ENSMUST00000033473.11	utr3
Kcnip3	1.56374	0.0323594	127457566	127457745	ENSMUST00000103215.10	utr3
Gpm6b	1.54864	0.0186209	166386509	166386719	ENSMUST00000112235.7	utr3
Cops8	1.53475	0.00676083	90612895	90613133	ENSMUST00000036153.11	utr3
Ubr4	1.52944	0.0275423	139489377	139489558	ENSMUST00000097822.9	utr3
Mpp3	1.52838	0.0245471	102000310	102000641	ENSMUST00000062801.10	utr3, cds

Mpp3	1.52838	0.0245471	102000310	102000641	ENSMUST00000100400.8	utr3, cds
Ubr4	1.52838	0.0346737	139489379	139489530	ENSMUST00000165860.7	utr3
Kpnb1	1.52732	0.0446684	97159713	97159864	ENSMUST00000001479.4	utr3
Mpp3	1.51992	0.0251189	102000305	102000633	ENSMUST00000107168.7	utr3, cds
Frmd4a	1.51467	0.0436516	4611529	4612009	ENSMUST00000075767.13	utr3
Ppp3r1	1.5042	0.0158489	17200314	17200375	ENSMUST00000102880.4	utr3
Bnip3l	1.50108	0.0436516	66989415	66999534	ENSMUST00000022634.8	cds

Note: Rescaled hypergeometric test was used for data analysis.

Supplementary Table 5. Significantly downregulated m^6A modification peaks (PT+EV-circSCMH1 vs PT+EV-Vector)

Gene_name	fold_change	*P*_value	Peak_start	Peak_end	Trans_id	Peak_region
Plpp3	**0.133046**	**2.51E-11**	**105232644**	**105232764**	**ENSMUST00000064139.7**	**utr3**
Gpm6b	0.279322	2.51E-19	166388898	166388988	ENSMUST00000060210.13	utr3
Gpm6b	0.279322	2.51E-19	166388898	166388988	ENSMUST00000112233.7	utr3
Gpm6b	0.283221	6.31E-19	166388897	166388988	ENSMUST00000112235.7	utr3
Gpm6b	0.283221	6.31E-19	166388897	166388988	ENSMUST00000112228.7	utr3
Gpm6b	0.283221	6.31E-19	166388897	166388988	ENSMUST00000112229.8	utr3
Gpm6b	0.283221	6.31E-19	166388897	166388988	ENSMUST00000112226.2	utr3
Gpm6b	0.283221	6.31E-19	166388897	166388988	ENSMUST00000112223.7	utr3
Gpm6b	0.283221	6.31E-19	166388897	166388988	ENSMUST00000112227.8	utr3
Gpm6b	0.283221	6.31E-19	166388897	166388988	ENSMUST00000112224.7	utr3
Acsl3	0.295248	0.002692	78706877	78706967	ENSMUST00000142704.7	utr3
Acsl3	0.29937	0.001318	78706874	78706965	ENSMUST00000035779.14	utr3
Hnrnph1	0.301452	0.012303	50386437	50386527	ENSMUST00000069304.13	utr3
Hnrnph1	0.307786	0.014125	50386437	50386528	ENSMUST00000109142.7	utr3
Hnrnph1	0.307786	0.014125	50386437	50386528	ENSMUST00000077817.7	utr3
Akap17b	0.353553	0.025119	36611777	36611957	ENSMUST00000051906.12	cds
Mfsd2a	0.403321	0.006026	122946849	122946999	ENSMUST00000030408.11	utr3
Mgat5	0.423373	0.020893	127487856	127488007	ENSMUST00000038361.10	utr3
Zzef1	0.429283	0.000363	72926817	72927118	ENSMUST00000207107.1	utr3
Zzef1	0.432269	0.000417	72926819	72927120	ENSMUST00000069395.6	utr3
Zzef1	0.432269	0.000417	72926819	72927120	ENSMUST00000172220.7	utr3
C1qa	0.444421	1.26E-14	136895916	136896065	ENSMUST00000046285.5	utr3
Slc2a3	0.444421	0.012883	122727838	122727929	ENSMUST00000032476.10	utr3
Tdrkh	0.466516	0.044668	94425977	94426128	ENSMUST00000196386.4	utr3
Pafah1b1	0.486327	0.012589	74673978	74674039	ENSMUST00000021091.14	utr3
Tpm3	0.493116	0.013804	90086496	90087485	ENSMUST00000127955.1	cds, utr5
Epha5	0.496546	0.040738	84416626	84416806	ENSMUST00000113399.5	utr5

Epha5	0.496546	0.040738	84416626	84416806	ENSMUST00000113406.7	utr5
Pafah1b1	0.5	0.014791	74673979	74674040	ENSMUST00000102520.8	utr3
Tdrkh	0.504526	0.039811	94425948	94426154	ENSMUST00000200486.4	cds, utr3
Epha5	0.505226	0.038019	84416638	84416819	ENSMUST00000113401.3	utr5
Epha5	0.506628	0.044668	84416625	84416806	ENSMUST00000113403.7	utr5
Epha5	0.506628	0.044668	84416625	84416806	ENSMUST00000113398.7	utr5
Ubr4	0.510152	0.000562	139489407	139489558	ENSMUST00000097822.9	utr3
Fgf13	0.512989	0.026303	59062620	59062799	ENSMUST00000033473.11	utr3
Ubr4	0.512989	0.00123	139489409	139489530	ENSMUST00000165860.7	utr3
Isca2	0.514057	0.019953	84774966	84775085	ENSMUST00000021667.6	utr3
Epha5	0.516199	0.030903	84416621	84416831	ENSMUST00000053733.14	utr5
Fgf13	0.516915	0.026915	59062621	59062801	ENSMUST00000119833.7	utr3
Fgf13	0.530344	0.033884	59062622	59062802	ENSMUST00000119306.1	utr3
Nap1l4	0.534403	0.039811	143534366	143549104	ENSMUST00000207948.1	utr5
Luzp2	0.53663	0.035481	55268167	55268258	ENSMUST00000082373.7	utr3
Slc43a2	0.537002	0.015488	75577061	75577361	ENSMUST00000169547.8	utr3
Apc	0.537747	0.044668	34276605	34279285	ENSMUST00000066133.6	cds
Grin2b	0.540737	0.002291	135713232	135713383	ENSMUST00000053880.12	utr3
Apc	0.541863	0.045709	34276596	34279278	ENSMUST00000115781.9	cds
Tmem35a	0.549046	0.036308	134304860	134305011	ENSMUST00000037687.7	utr3
Slc43a2	0.549427	0.018621	75577065	75577365	ENSMUST00000042561.13	utr3
Plxnb2	0.550189	0.01122	89155548	89155788	ENSMUST00000109331.7	utr3
Plxnb2	0.550189	0.01122	89155548	89155788	ENSMUST00000060808.9	utr3
Scn2b	0.551717	0.035481	45127294	45127444	ENSMUST00000170998.8	utr3
Dhx9	0.554785	0.003981	153455846	153456026	ENSMUST00000186380.6	utr3
H13	0.554785	0.000708	152704752	152705112	ENSMUST00000125366.7	utr3
Bnip3l	0.558644	0.017378	66989445	66993933	ENSMUST00000022634.8	cds
Araf	0.559806	0.001479	20852680	20852859	ENSMUST00000120356.7	utr3
Dhx9	0.560972	0.004571	153455847	153456028	ENSMUST00000042141.11	utr3

Bnip3l	0.56331	0.019953	66989426	66993913	ENSMUST00000111115.7	cds
Kcnip3	0.566442	0.012589	127457566	127457715	ENSMUST00000103215.10	utr3
Kcnip3	0.569197	0.012883	127457576	127457727	ENSMUST00000028850.14	utr3
Usp10	0.577543	0.023442	119957380	119957560	ENSMUST00000108988.8	utr3
Lmbrd1	0.581157	0.015849	24762835	24763015	ENSMUST00000095062.9	utr3
Arhgap21	0.582367	0.018197	20847918	20848069	ENSMUST00000154230.8	utr3
Arhgap21	0.582367	0.018197	20847918	20848068	ENSMUST00000114594.7	utr3
Brd9	0.586011	0.007762	73960745	73960895	ENSMUST00000099384.3	utr3
Brd9	0.586011	0.007762	73960743	73960894	ENSMUST00000222399.1	utr3
Usp10	0.587231	0.027542	119957375	119957555	ENSMUST00000144458.7	utr3
Prrc2c	0.587638	0.002344	162671934	162672234	ENSMUST00000183223.7	utr3
Arhgap21	0.592957	0.021878	20847939	20848090	ENSMUST00000173194.7	utr3
Prrc2c	0.594604	0.002692	162671924	162672225	ENSMUST00000182660.7	utr3
Txndc11	0.596668	0.014454	11074910	11075208	ENSMUST00000038424.13	utr3, cds
Araf	0.599155	0.002951	20852665	20852845	ENSMUST00000122312.7	utr3
Dst	0.601235	0.019498	34308510	34308571	ENSMUST00000097785.9	utr3
Ntrk2	0.601652	0.013804	58932462	58932881	ENSMUST00000224259.1	utr3
Dnajc11	0.604159	0.0302	151981066	151981245	ENSMUST00000062904.10	utr3
Prrc2c	0.604578	0.003548	162671922	162672222	ENSMUST00000182149.7	utr3
Fam49a	0.605417	0.015136	12376238	12376389	ENSMUST00000069066.13	utr3
H13	0.607518	0.00631	152704769	152705036	ENSMUST00000089059.8	utr3
Prrc2c	0.60794	0.002455	162671907	162672236	ENSMUST00000182393.7	utr3
Numa1	0.610473	0.012589	101999241	101999689	ENSMUST00000209639.1	cds
Brd2	0.612593	0.022387	34112022	34112202	ENSMUST00000114242.8	utr3
Brd2	0.612593	0.021878	34112022	34112203	ENSMUST00000025193.12	utr3
Ptges3	0.616426	0.01122	128077032	128077272	ENSMUST00000052798.13	utr3
Ntrk2	0.622437	0.017378	58932453	58932872	ENSMUST00000109838.9	utr3
Numa1	0.624165	0.010233	101999248	101999758	ENSMUST00000084852.12	cds
Numa1	0.624165	0.010471	101999258	101999769	ENSMUST00000210475.1	cds

Frmd4a	0.628942	0.019498	4611512	4612023	ENSMUST00000091497.10	utr3
Frmd4a	0.629379	0.016982	4611509	4612047	ENSMUST00000176828.7	utr3
Lmbrd1	0.631126	0.032359	24762818	24762998	ENSMUST00000191471.6	utr3
Frmd4a	0.635516	0.02138	4611521	4612032	ENSMUST00000177457.7	utr3
Arl8b	0.636397	0.008318	108823578	108823758	ENSMUST00000032196.8	utr3
Kpnb1	0.64038	0.022909	97159713	97159864	ENSMUST00000001479.4	utr3
Frmd4a	0.640824	0.020893	4611500	4612039	ENSMUST00000075767.13	utr3
Slc38a1	0.640824	0.045709	96571417	96571567	ENSMUST00000088452.10	utr3
Slc38a1	0.640824	0.045709	96571417	96571568	ENSMUST00000088454.11	utr3
Slc38a1	0.640824	0.045709	96571417	96571568	ENSMUST00000100262.2	utr3
Cops8	0.644387	0.005129	90612895	90613133	ENSMUST00000036153.11	utr3
Mpp3	0.644834	0.017783	102000280	102000581	ENSMUST00000062801.10	utr3, cds
Mpp3	0.644834	0.017783	102000280	102000581	ENSMUST00000100400.8	utr3, cds
Mpp3	0.648869	0.022909	102000305	102000574	ENSMUST00000107168.7	utr3, cds
Cyfip2	0.660211	0.003467	46196100	46196250	ENSMUST00000142017.1	utr3
Ppp3r1	0.662963	0.012023	17200314	17200375	ENSMUST00000102880.4	utr3

Note: Rescaled hypergeometric test was used for data analysis.

Supplementary Table 6. The primer sequences used for RT-PCR analysis

List of oligonucleotide sequences	5’>3’
circSCMH1 (mouse)-F	CTACTGGTGCCGCTTTGACT
circSCMH1 (mouse)-R	GGCACCTGTCAATCCAACGA
circSCMH1 (human)-F	CACTTTTGATGGGTGGCGAG
circSCMH1 (human)-R	TCGTTGCTTGGAGGTGTGTAG
Scmh1 (mouse)-F	GTCAGAGGTCAGAGCAAGGT
Scmh1 (mouse)-R	ATTCCAGTCACTAGGCTGCAT
circHECW2 (mouse)-F	AACAGGGACCTCGTGGGATT
circHECW2 (mouse)-R	GGCTGTCAATCCGTGCCTC
Gapdh (mouse)-F	AGGTCGGTGTGAACGGATTTG
Gapdh (mouse)-R	TGTAGACCATGTAGTTGAGGTCA
PLPP3 (human)-F	CTGCGTCCTCACTTCTTGAGT
PLPP3 (human)-R	ACACGAAGAAAACTATGCAGC
Plpp3 (mouse)-F	ATGCTGTATCTGGTGCTCTACC
Plpp3 (mouse)-R	AGCTCCTTGGGCAAATCCTG
Plpp3-peak (mouse)-F	TGTCAGTATTTTTGTAAGATTCCGT
Plpp3-peak (mouse)-R	TGGTTAGTTGTGTTATGAGCTTTT
Hnrnph1 (mouse)-F	AAATGGGGCTCAAGGTATTCG
Hnrnph1 (mouse)-R	GGACCAGTATGCTTCAACACC
Akap17b (mouse)-F	AAGAACAAGACCATGATAAGGGC
Akap17b (mouse)-R	GCAAACCACTTACAGGGCAAG
Mgat (mouse)-F	CTATGGATGGCTACCCCCACT
Mgat (mouse)-R	AAGGTAAACGAGGACACCAATTT
Zzef1 (mouse)-F	GTCGCAACAGAAGTGGAAAGA
Zzef1 (mouse)-R	TCATGTCCGAGGTAAATCGGT
Acsl3 (mouse)-F	AACCACGTATCTTCAACACCATC
Acsl3 (mouse)-R	AGTCCGGTTTGGAACTGACAG
Epha5 (mouse)-F	ATGGTACCTGCCAAGCTCCT

Epha5 (mouse)-R	CCCTCGGCTGTAATACTGGTC
Gpm6b (mouse)-F	AACTTGTGCCACCGGCTATC
Gpm6b (mouse)-R	CAGCATTCGAAGCAACCCAT
Ubc13 (mouse)-F	GCTGGCAGAACCAGTTCCT
Ubc13 (mouse)-R	TCCCTCAAAGGGGGAATCCTG
YTHDF2 (human)-F	AGCCCCACTTCCTACCAGATG
YTHDF2 (human)-R	TGAGAACTGTTATTTCCCCATGC
Ythdf2 (mouse)-F	GAGCAGAGACCAAAAGGTCAAG
Ythdf2 (mouse)-R	CTGTGGGCTCAAGTAAGGTTC
ACTB (human)-F	GTTACAGGAAGTCCCTTGCCATC
ACTB (human)-R	TGTGGACTTGGGAGAGGACTG
Actb (mouse)-F	GGCTGTATTCCCCTCCATCG
Actb (mouse)-R	CCAGTTGGTAACAATGCCATGT
Fto (mouse)-F	CCTCTTCACCAGGGAGACTG
Fto (mouse)-R	GCCACTCAAACTCCACCTCAT
5’arms-F	ATGCCCACCAAAGTCATCAGTGTAG
5’arms-R	AGGCGGGCCATTTACCGTAAGTTA
3’arms-F	CCTCCTCTCCTGACTACTCCCAGTC
3’arms-R	TCACAGAAACCATATGGCGCTCC
WT-F	AGTCTTTCCCTTGCCTCTGCT
WT-R	GGGTCTTCCACCTTTCTTCAG

Supplementary Table 7. Details about antibodies

Antibody	Vendor	Catalog Number	Concentration
APC anti-Mouse NCAM-1/CD56 Allophycocyanin MAb	R&D	FAB7820A-100	1μl:2x10^5 cells (Flow Cyt)
Brilliant Violet 605™ anti-mouse CD31	BioLegend	102427	1μl:2x10^5 cells (Flow Cyt)
FITC anti-mouse/human CD11b Antibody	BioLegend	101205	1μl:1x10^6 cells (Flow Cyt)
PE anti-mouse ACSA-2	Miltenyi Biotec	130-116-244	1μl:2x10^5 cells (Flow Cyt)
PerCp-CyTM5.5 anti-mouse CD45 Antibody	BD Pharmingen	561869	1μl:1x10^6 cells (Flow Cyt)
CD31	Abcam	ab9498	1:50 (IF)
CD31	Invitrogen	PA5-32321	1:100 (IF)
BrdU	SantaCruz	sc-51514	1:100 (IF)
Biotinylated-Isolectin B_4	SigmaAldrich	L2140	1:200 (IF)
ZO-1	Proteintech	21773-1-AP	1:1000 (WB)
Occludin	Proteintech	13409-1-AP	1:1000 (WB)
Claudin-5	Affinity	AF5216	1:1000 (WB)
CD13	R&D	AF2335	1:400 (IF)
CD31	Proteintech	28083-1-AP	1:2000 (WB)
FTO	Proteintech	27226-1-AP	1:1000 (WB)
ALKBH5	Proteintech	16837-1-AP	1:1000 (WB)
METTL3	Proteintech	15073-1-AP	1:1000 (WB)
METTL14	Proteintech	26158-1-AP	1:1000 (WB)
Synaptophysin	Proteintech	17785-1-AP	1:2000 (WB)
WTAP	Proteintech	10200-1-AP	1:1000 (WB)
UBC13	Proteintech	10243-1-AP	1:1000 (WB)
Ub-K63	Abways	CY6579	1:2000 (WB)
LPP3/PPAP2B	Invitrogen	PA5-90665	1:1000 (WB)

β-actin	Proteintech	66009-1-Ig	1:3000 (WB)
Histone-H3	Proteintech	17168-1-AP	1:3000 (WB)
HRP-conjugated Affinipure goat anti-mouse IgG (H+L)	Proteintech	SA00001 -1	1:2,000 (WB)
HRP-conjugated Affinipure goat anti-rabbit IgG (H+L)	Proteintech	SA00001-2	1:2,000 (WB)
Alexa Flour™ 488 goat anti-mouse IgG secondary antibody	Invitrogen	A11001	1:300 (IF)
Alexa Flour™ 594 goat anti-mouse IgG secondary antibody	Invitrogen	A11005	1:300 (IF)
Alexa Flour™ 594 goat anti-rabbit IgG secondary antibody	Invitrogen	A11012	1:300 (IF)
Alexa Flour™ 488 goat anti-rabbit IgG secondary antibody	Invitrogen	A11008	1:300 (IF)
Streptavidin-FITC secondary antibody	Invitrogen	434311	1:100 (IF)

Notes: Flow Cyt: flow cytometry; IF: Immunofluorescence; WB: Western Blot.

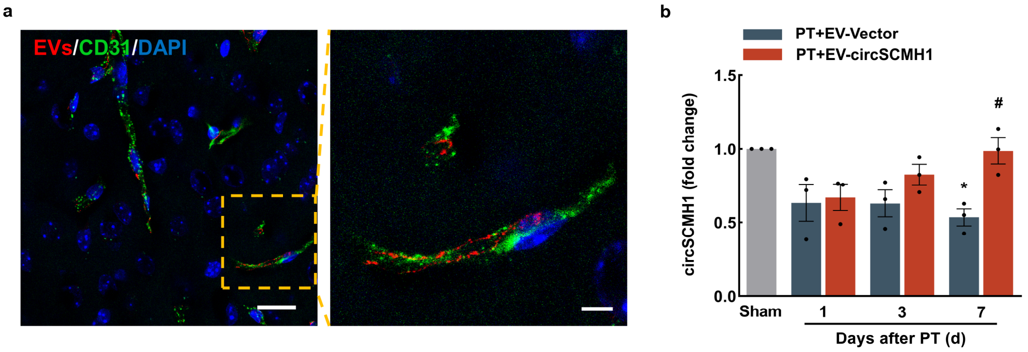

Supplementary Fig. 1 Relative expression of circSCMH1 in the sorted endothelial cells from PT model mice with EV-circSCMH1 treatment.

a, The distribution of EVs in endothelial cells ($CD31^{+}$) at 6 hours after intravenous injection. Scale bars, 20 μm (overview), 5 μm (insets). Red dots, DiI^{+} EVs. Data are representative of three independent experiments. **b**, ECs were isolated from peri-infarct tissues of PT stroke mice with EV-circSCMH1 treatment. Relative expression of circSCMH1 in the sorted ECs as determined by qPCR. n=3 samples for each group. Each sample was pooled from 8 animals. $^{*}P$=0.0308 versus sham, $^{\#}P$=0.0372 versus PT+EV-Vector. The data in **b** were expressed as means±SEM; one-way ANOVA followed by Holm-Sidak post hoc multiple comparison test. Source data are provided as a Source Data file. CD31: platelet endothelial cell adhesion molecule-1; circSCMH1: circular RNA SCMH1; DAPI: 4',6-Diamidino-2-phenylindole; d: day; EV: extracellular vesicle; PT: photothrombotic.

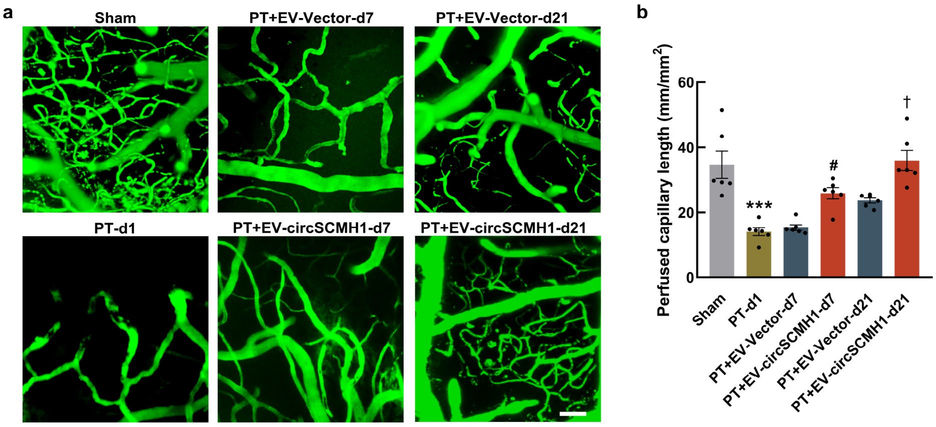

Supplementary Fig. 2 *In-vivo* multiphoton microscopic images in cortical capillaries.

a, Representative *in vivo* multiphoton microscopic images of cortical capillaries in real time in the living mouse brain. For visualization of the brain vasculature, FITC-dextran (MW=2,000,000 Da) was injected intravenously just before the imaging experiment. Scale bars, 50 μm. **b**, Perfused capillary length was analyzed by Angitool. n=6 mice/group. $^{***}P$<0.0001 versus sham; $^{\#}P$=0.0303 versus PT+EV-Vector-d7; †P=0.0105 versus PT+EV-Vector-d21. The data in **b** were expressed as means±SEM; one-way ANOVA followed by Holm-Sidak post hoc multiple comparison test. Source data are provided as a Source Data file. circSCMH1: circular RNA SCMH1; d: day; EV: extracellular vesicle; PT: photothrombotic.

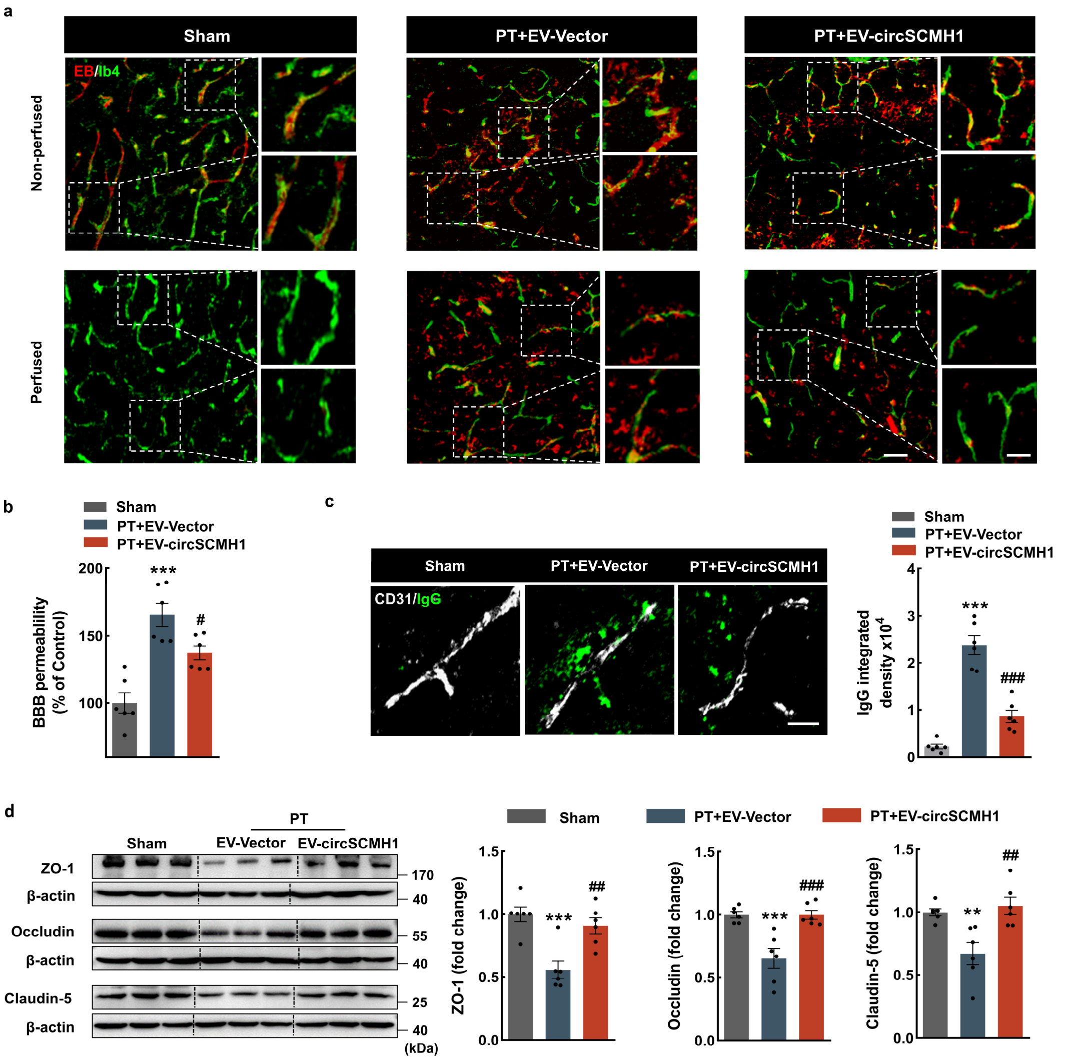

Supplementary Fig. 3 EV-mediated circSCMH1 maintained the integrity of the blood-brain barrier during stroke recovery.

a, Representative images of Evans blue extravascular deposits in the peri-infarct cortex at day 21 after PT. Scale bars, 20 μm (overview), 5 μm (insets). **b**, Cerebrovascular permeability was determined by measuring the concentrations of brain-extracted Evans blue using spectrophotometry at 620 nm at day 21 after PT. n=6 mice/group, $^{***}P<0.0001$ versus sham; $^{\#}P=0.0142$ versus PT+EV-Vector. **c**, Representative images of IgG extravascular deposits in the peri-infarct cortex at day 21 after PT. Scale bars, 10 μm. the IgG integrated density was measured by ImageJ. n=6 mice/group, $^{***}P<0.0001$ versus sham; $^{\#\#\#}P<0.0001$ versus PT+EV-Vector. **d**, Western blot analysis of ZO-1, Occludin and Claudin-5 in the peri-infarct cortex at day 28 after PT. Three representative immunoblots were presented from 6 mice/group. $^{**}P=0.0068$ (Claudin-5), $^{***}P=0.0006$ (ZO-1), $^{***}P=0.0009$ (Occludin) versus sham; $^{\#\#}P=0.0030$ (ZO-1), $^{\#\#}P=0.0034$ (Claudin-5), $^{\#\#\#}P=0.0009$ (Occludin) versus PT+EV-Vector. The data **b**, **c** and **d** were expressed as means±SEM; one-way ANOVA followed by Holm-Sidak post hoc multiple comparison test. Source data are provided as a Source Data file. BBB: blood-brain barrier; CD31: platelet endothelial cell adhesion molecule-1; circSCMH1: circular RNA SCMH1; EB: Evans blue; EV: extracellular vesicle; Ib4: isolectin-B_4; IgG: immunoglobulin G; PT: photothrombotic; ZO-1: zona occludens 1.

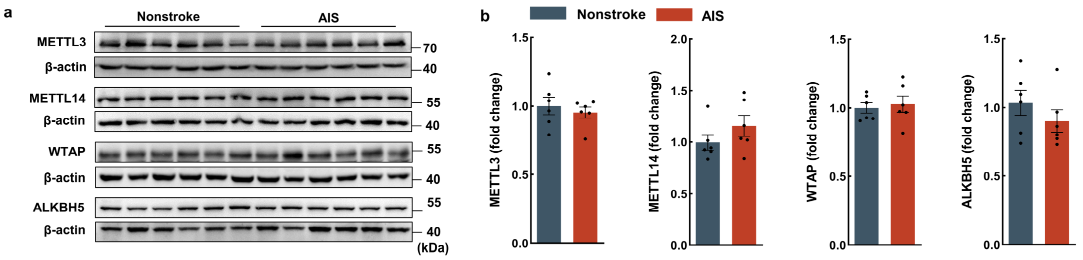

Supplementary Fig. 4. The expression of METTL3, METTL14, WTAP, and ALKBH5 in AIS patients.

a and **b**, Western blot analysis of METTL3, METTL14, WTAP, and ALKBH5 in the somatosensory cortex of AIS patients. There were 6 individuals/group. The significance was analyzed using the Student *t*-test (two-sided). All data were expressed as mean±SEM. Source data are provided as a Source Data file. ALKBH5: alkB homolog 5; AIS: acute ischemic stroke; circSCMH1: circular RNA SCMH1; METTL3: methyltransferase like 3; METTL14: methyltransferase like 14; WTAP: Wilms tumor 1-associated protein.

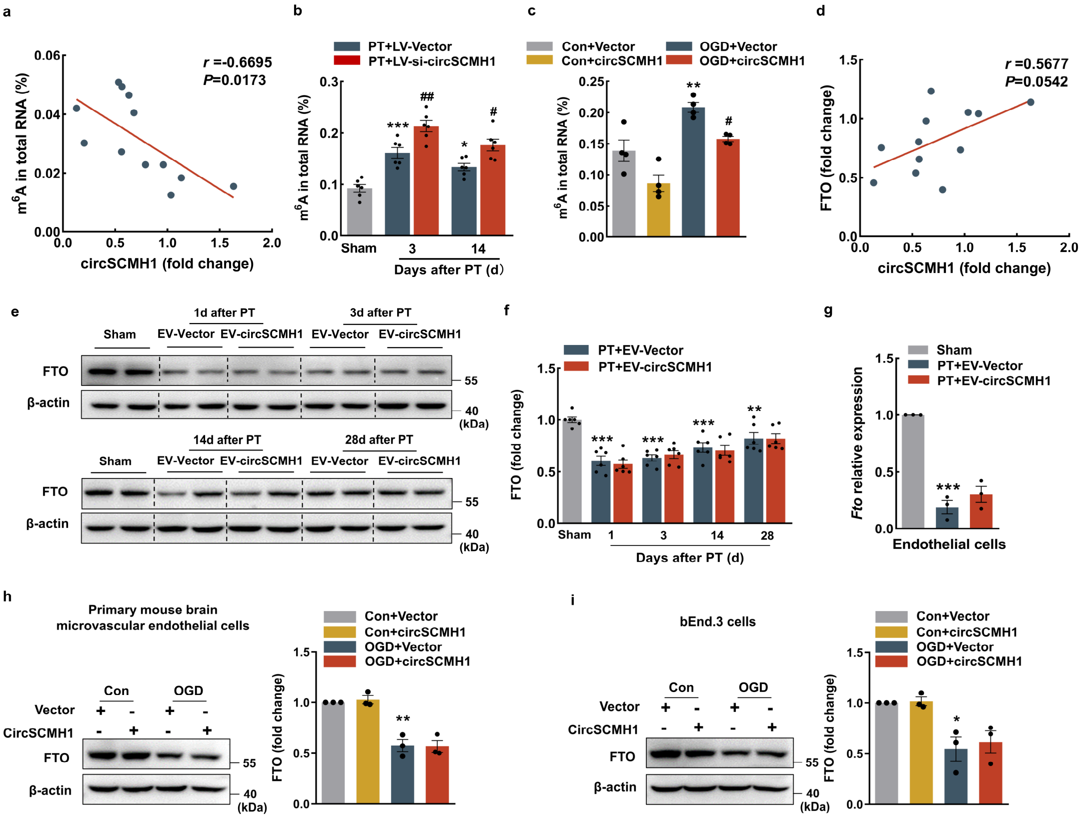

Supplementary Fig. 5 circSCMH1 reduced the level of m^{6}A but did not affect the FTO level after stroke.

a, Correlation analysis was performed based on the circSCMH1 and m^{6}A level (Pearson correlation coefficient *r*=-0.6695, *P*=0.0173, two-sided, n=12) in the somatosensory cortex of nonstroke and AIS patients. **b**, Total RNA was extracted from the peri-infarct cortex of PT mice injected with LV-si-circSCMH1, and m^{6}A levels were determined as the percentage of all adenosine residues in RNA. n=6 mice/group, $^{*}P$=0.0235, $^{***}P$=0.0002 versus sham, $^{\#}P$=0.0218, $^{\#\#}P$=0.0049 versus PT+LV-Vector. **c**, The m^{6}A levels were determined in bEnd.3 cells treated with circSCMH1 overexpression plasmid. Data were presented by 3 independent experiments. $^{**}P$=0.0065 versus Con+Vector, $^{\#}P$=0.0440 versus OGD+Vector. **d**, Correlation analysis between the circSCMH1 and FTO level (Pearson correlation coefficient *r*=0.5677, *P*=0.0542, two-sided, n=12) in the somatosensory cortex of nonstroke and AIS patients. **e, and f**, Western blot analysis of FTO expression after stroke in mice with EV-circSCMH1. Two representative immunoblots were presented from 6 mice/group. $^{**}P$=0.0085 (28d), $^{***}P$<0.0001 (1d and 3d), $^{***}P$=0.0002 (14d) versus sham. **g**, ECs were isolated from the peri-infarct cortex of mice at day 3 after PT. Relative expression of *Fto* mRNA in the sorted ECs as determined by qPCR. n=3 samples for each group. Each sample was pooled from 8 animals. $^{***}P$=0.0001 versus sham. **h**, and **i**, Western blot analysis of FTO after OGD treatment in the primary mouse brain microvascular ECs (**h**) or bEnd.3 cells **(i)**. Data were presented by 3 independent experiments. $^{*}P$=0.0134, $^{**}P$=0.0028 versus Con+Vector. The data in **b**, **f** and **g** were expressed as means±SEM; one-way ANOVA followed by Holm-Sidak post hoc multiple comparison test. The data in **c**, **h** and **i** were expressed as the mean±SEM; two-way ANOVA followed by Bonferroni's post hoc multiple comparison tests. Source data are provided as a Source Data file. AIS: acute ischemic stroke; circSCMH1: circular RNA SCMH1; Con: control; d: day; EV: extracellular vesicle;

FTO: fat mass and obesity-associated protein; LV: lentivirus; m^6A: N^6-methyladenosine; OGD: oxygen glucose deprivation; PT: photothrombotic.

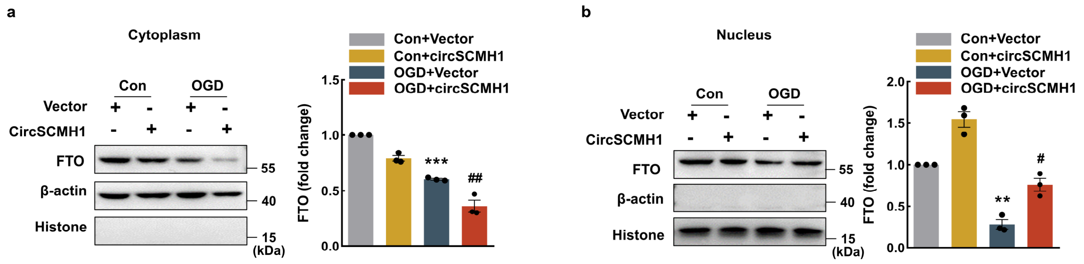

Supplementary Fig. 6 CircSCMH1 altered the subcellular localization of FTO in bEnd.3 cells. **a** and **b**, Western blot analysis of FTO expression in the cytoplasm **(a)** and nucleus **(b)** of bEnd.3 cells. Data were presented by 3 independent experiments. $^{**}P$=0.0029, $^{***}P$=0.0007 versus the Con+Vector group. $^{\#}P$=0.0235, $^{\#\#}P$=0.0090 versus the OGD+Vector group. The data in **a** and **b** were expressed as the mean±SEM; two-way ANOVA followed by Bonferroni's post hoc multiple comparison tests. Source data are provided as a Source Data file. circSCMH1: circular RNA SCMH1; Con: control; FTO: fat mass and obesity-associated protein; OGD: oxygen glucose deprivation.

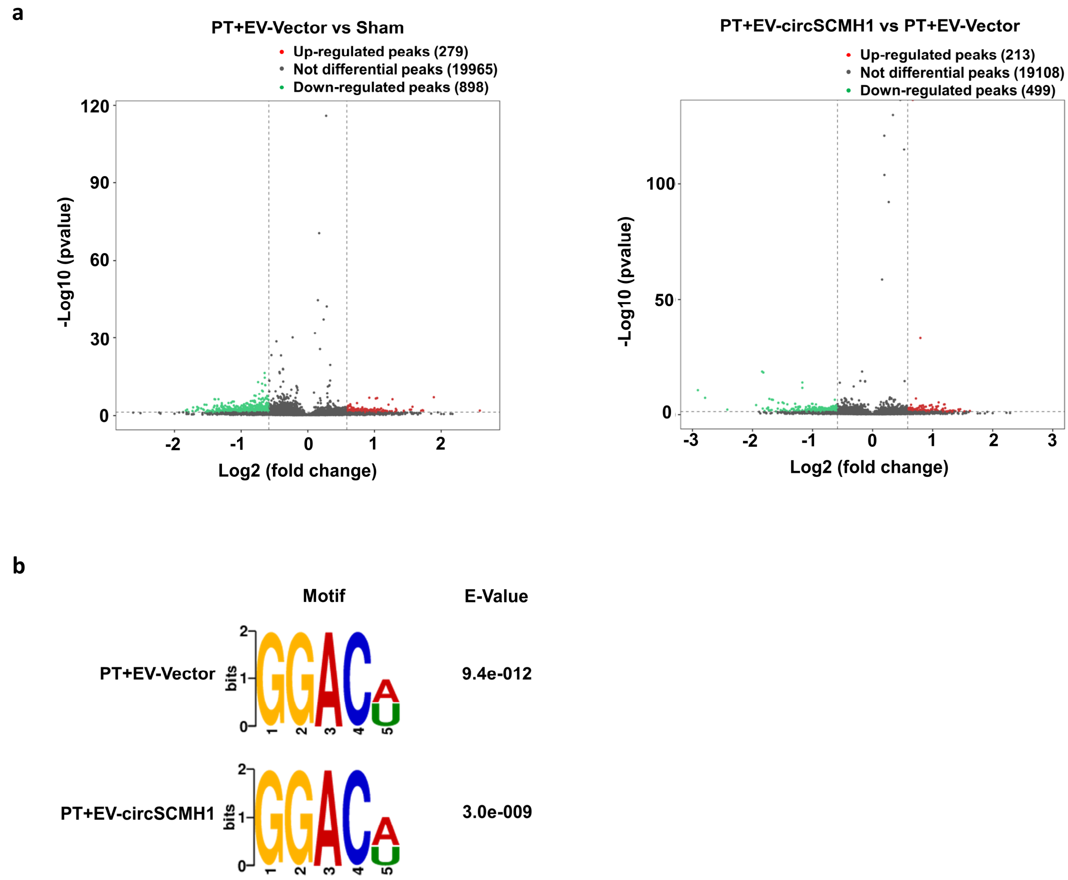

Supplementary Fig. 7 m^{6}A-sequence of the peri-infarct cortex of PT mice after EV-circSCMH1 administration.

a, Significant upregulation and downregulation in m^{6}A methylation peaks of transcripts (fold change≥1.5, P<0.05, rescaled hypergeometric test). Total RNA was extracted from the control cortex of 12 sham mice, the peri-infarct cortex of 12 mice with EV-Vector administration at day 14 post PT, and the peri-infarct cortex of 12 mice with EV-circSCMH1 administration at day 14 post PT. MeRIP sequence analysis was performed after mRNA purification. **b**, Top consensus m^{6}A motif identified by DREME 4.12.0 with m^{6}A peaks in the peri-infarct cortex of PT mice after EV-Vector or EV-circSCMH1 administration. circSCMH1: circular RNA SCMH1; EV: extracellular vesicle; PT: photothrombotic.

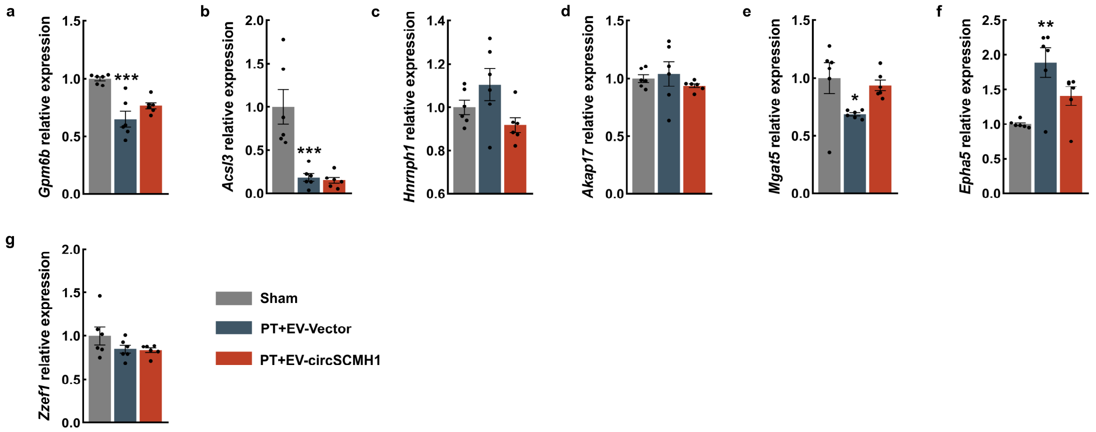

Supplementary Fig. 8 The target gene expression of *Gpm6b, Acsl3, Hnrnph1, Akap17, Mgat5, Epha5* and *Zzef1* in the peri-infarct cortex of mice at day 14 after PT model induction.
a-g, The effect of EV-circSCMH1 on the levels of target gene was evaluated by qPCR in peri-infarct cortex of mice at day 14 post PT. n=6 mice/group. $^{*}P$=0.0499 (**e**), $^{**}P$=0.0019 (**f**), $^{***}P$=0.0001 (**a**), $^{***}P$=0.0005 (**b**) versus sham. All data were expressed as means±SEM; one-way ANOVA followed by Holm-Sidak post hoc multiple comparison test. Source data are provided as a Source Data file. *Acsl3*: Acyl-CoA synthetase long chain family member 3; *Akap17b*: A kinase (PRKA) anchor protein 17B; circSCMH1: circular RNA SCMH1; EV: extracellular vesicle; *Epha5*: EPH receptor A5; *Gpm6b*: glycoprotein M6B; *Hnrnph1*: heterogeneous nuclear ribonucleoprotein H1; *Mgat5*: mannoside acetylglucosaminyltransferase 5; PT: photothrombotic. *Zzef1*: zinc finger ZZ-type with EF hand domain 1.

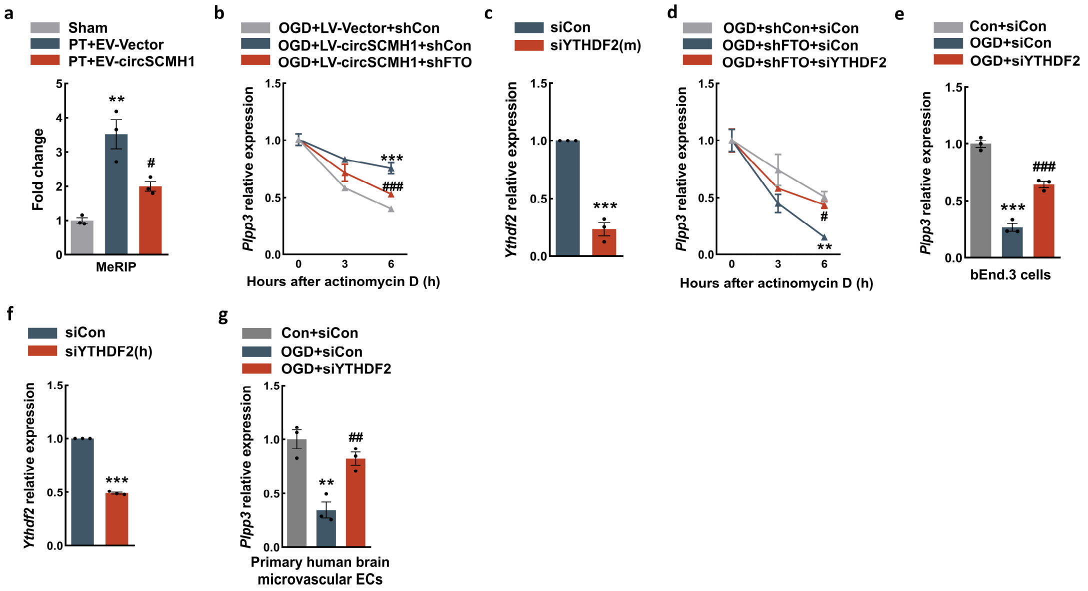

Supplementary Fig. 9 CircSCMH1 increased mRNA stability of *Plpp3* via m^{6}A.

a, The cerebral microvessels were collected from the peri-infarct cortex of 8 mice at day 14 after PT. Specific primer against the m^{6}A peak were designed to amplify the m^{6}A peak of *Plpp3* transcript. Data were presented by 3 independent experiments. $^{**}P$=0.0016 versus sham, $^{\#}P$=0.0135 versus PT+EV-Vector. **b**, *Plpp3* mRNA stability assay after transfection with LV-circSCMH1 and shFTO in bEnd.3 cells. Data were presented by 4 independent experiments. $^{***}P$<0.0001 versus OGD+LV-Vector+shCon; $^{\#\#\#}P$=0.0003 versus OGD+LV-circSCMH1+shCon. **c**, The bEnd.3 cells were transfected with mouse siYTHDF2, the level of *Ythdf2* mRNA was measured by qPCR. Data were presented by 3 independent experiments. $^{***}P$<0.0001 versus siCon group. **d**, *Plpp3* mRNA stability assay after transfection with shFTO and siYTHDF2 in bEnd.3 cells. Data were presented by 3 independent experiments. $^{**}P$=0.0092 versus OGD+shCon+siCon; $^{\#}P$=0.0269 versus OGD+shFTO+siCon. **e**, The bEnd.3 cells were transfected with siYTHDF2, the level of *Plpp3* mRNA was measured in OGD-induced bEnd.3 cells by qPCR. Data were presented by 3 independent experiments. $^{***}P$<0.0001 versus Con+siCon, $^{\#\#\#}P$=0.0003 versus OGD+siCon. **f**, The primary human brain microvascular ECs were transfected with siYTHDF2, the level of *Ythdf2* mRNA was measured by qPCR. Data were presented by 3 independent experiments. $^{***}P$<0.0001 versus siCon. **g**, The primary human brain microvascular ECs were transfected with siYTHDF2, the level of *Plpp3* mRNA was measured by qPCR after OGD. Data were presented by 3 independent experiments. $^{**}P$=0.0026 versus Con+siCon, $^{\#\#}P$=0.0084 versus OGD+siCon. The data in **a**, **e** and **g** were expressed as means±SEM; one-way ANOVA followed by Holm-Sidak post hoc multiple comparison test. The data in **b** and **d** were expressed as means±SEM; two-way repeated measures ANOVA followed by Holm-Sidak post hoc multiple comparison test. The data in **c** and **f** were expressed as means±SEM; the Student *t* test (two-sided). Source data are provided as a Source Data file. Con: control; h: human; LV: lentivirus; m: mouse; OGD: oxygen glucose deprivation; *Plpp3*: lipid phosphate phosphatase 3; siCon: control siRNA; siYTHDF2: YTHDF2 siRNA; shCon: control short hairpin RNA; shFTO: FTO short hairpin RNA; *Ythdf2*: YT521-B homology domain family 2.

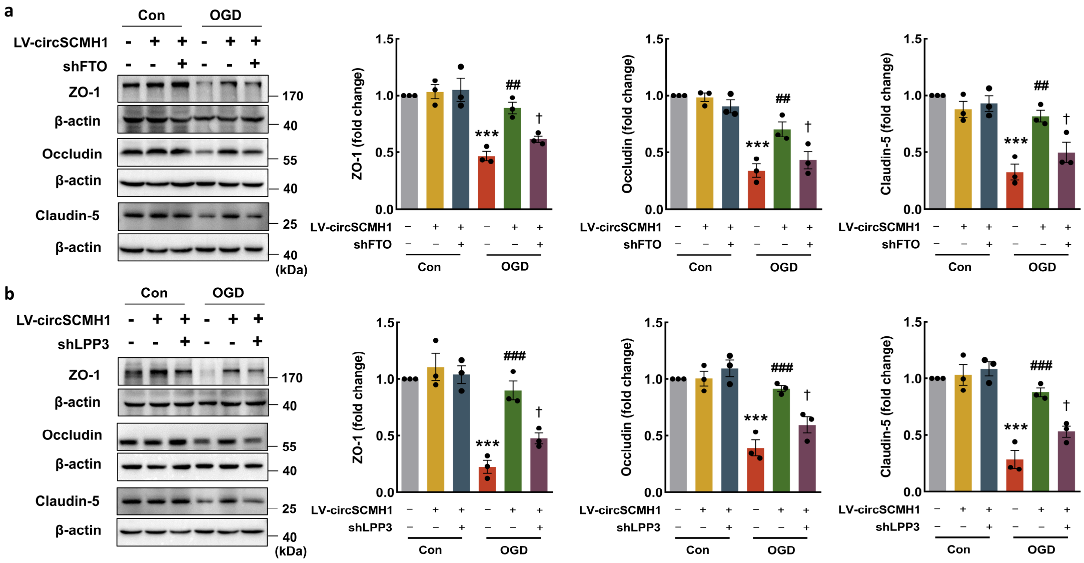

Supplementary Fig. 10 Levels of tight junction proteins ZO-1, Occludin, and Claudin-5 in bEnd.3 cells co-transfected with the circSCMH1 lentivirus and shFTO or shLPP3.

a and **b**, The expressions of ZO-1, Occludin, and Claudin-5 were detected in bEnd.3 cells after co-transfection with the circSCMH1 lentivirus and shFTO (**a**) or shLPP3 (**b**) in bEnd.3 cells. Data were presented by 3 independent experiments. $^{***}P$=0.0004 (**a** ZO-1), $^{***}P$<0.0001 (**a** Occludin and **b** Claudin-5), $^{***}P$=0.0001 (**a** Claudin-5, **b** ZO-1 and **b** Occludin) versus Con; $^{\#\#}P$=0.0025 (**a** ZO-1), $^{\#\#}P$=0.0048 (**a** Occludin), $^{\#\#}P$=0.0018 (**a** Claudin-5), $^{\#\#\#}P$=0.0004 (**b** ZO-1), $^{\#\#\#}P$=0.0005 (**b** Occludin), $^{\#\#\#}P$=0.0002 (**b** Claudin-5) versus OGD; $^{\dagger}P$=0.0434 (**a** ZO-1), $^{\dagger}P$=0.0264 (**a** Occludin), $^{\dagger}P$=0.0352 (**a** Claudin-5), $^{\dagger}P$=0.0130 (**b** ZO-1), $^{\dagger}P$=0.0181 (**b** Occludin), $^{\dagger}P$=0.0137 (**b** Claudin-5) versus OGD+LV-circSCMH1. The data in **a** and **b** were expressed as means±SEM; one-way ANOVA followed by Holm-Sidak post hoc multiple comparison test. Source data are provided as a Source Data file. CircSCMH1: circular RNA SCMH1; FTO: fat mass and obesity-associated protein; LV: lentivirus; shFTO: FTO short hairpin RNA; shLPP3: LPP3 short hairpin RNA; ZO-1: zona occludens 1.

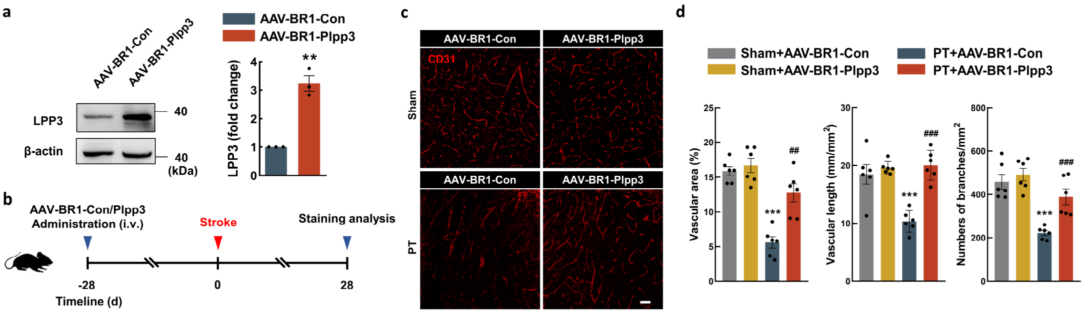

Supplementary Fig. 11 Endothelial-target *Plpp3* overexpression promoted vascular repair in PT mice.

a, The mice were subjected with AAV-BR1-Plpp3 by intravenous injection. The microvessels were collected from three mice at day 28 after AAV-BR1-Plpp3 injection. The expression of LPP3 was measured by western blot analysis in microvessels. Data were presented by 3 independent experiments. $^{**}P$=0.0013 versus AAV-BR1-Con. **b**, Schematic of AAV-BR1-Con/Plpp3 administration and staining analysis in mice. **c** and **d**, Representative images with CD31 staining showing blood vessels in the peri-infarct cortex at day 28 after PT in mice, followed by the analysis of vascular area fraction, total vascular length, and number of branches. n=6 mice/group. Scale bars, 50 µm. $^{***}P$<0.0001 (Vascular area and Number of branches), $^{***}P$=0.0010 (Vascular length) versus sham+AAV-BR1-Con; $^{\#\#}P$=0.0021 (Vascular area), $^{\#\#\#}P$=0.0002 (Vascular length), $^{\#\#\#}P$<0.0001 (Number of branches) versus PT+AAV-BR1-Con. The data in **a** were expressed as means±SEM; the Student *t* test (two-sided). The data in **d** were expressed as the mean±SEM; two-way ANOVA followed by Bonferroni's post hoc multiple comparison tests. Source data are provided as a Source Data file. AAV: adeno-associated virus; CD31: platelet endothelial cell adhesion molecule-1; *Plpp3*/LPP3: lipid phosphate phosphatase 3; PT: photothrombotic.

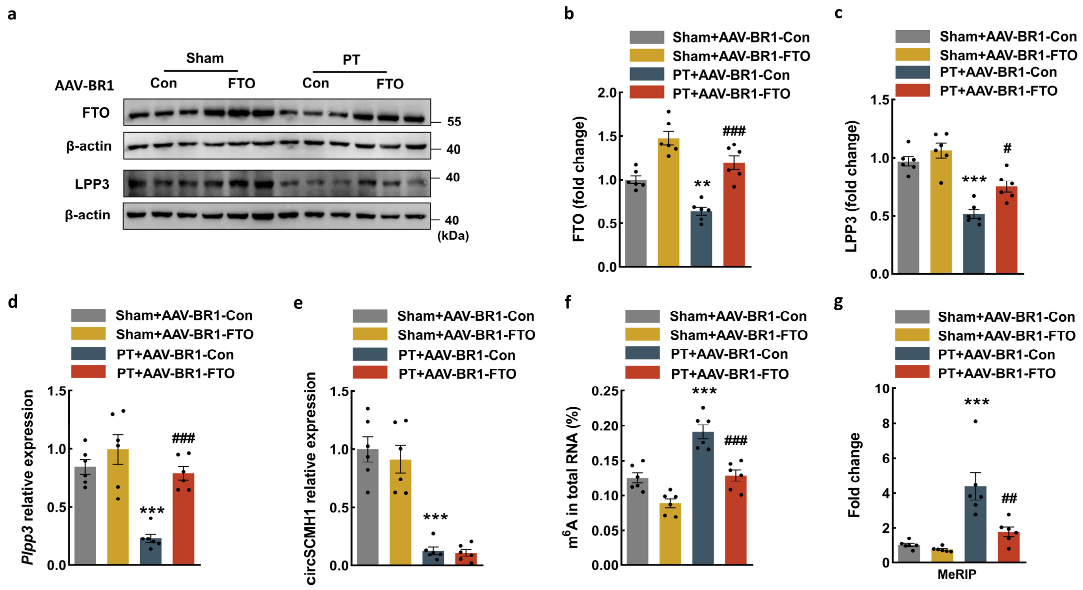

Supplementary Fig. 12 The effect of AAV-BR1-FTO on the levels of FTO, LPP3, circSCMH1, m^{6}A levels in total RNA and *Plpp3* expression and methylation levels.

a, **b** and **c,** The mice were administrated with AAV-BR1-FTO, and subjected to PT stroke at day 28 after AAV-BR1-FTO injection. The peri-infarct cortex was isolated to detection the level of FTO and LPP3 at day 3 after PT model induction. Three representative immunoblots were presented from 6 mice/group. $^{**}P$=0.0085, $^{***}P$=0.0002 versus sham+AAV-BR1-Con; $^{\#}P$=0.0459, $^{\#\#\#}P$=0.0001 versus PT+AAV-BR1-Con. **d**, **e**, **f** and **g,** The total RNA were extracted from the peri-infarct tissue of mice at day 3 after PT to determine the levels of circSCMH1 (**d**) and *Plpp3* (**e**), the m^{6}A levels in total RNA (**f**), the m^{6}A of *Plpp3* (**g**). n=6 mice/group, $^{***}P$=0.0003 (**d**), $^{***}P$<0.0001 (**e**), $^{***}P$=0.0005 (**f**), $^{***}P$=0.0002 (**g**) versus sham+AAV-BR1-Con; $^{\#\#}P$=0.0022 (**g**), $^{\#\#\#}P$=0.0009 (**d**), $^{\#\#\#}P$=0.0008 (**f**) versus PT+AAV-BR1-Con. Data in **b**, **c**, **d**, **e**, **f** and **g** were expressed as the mean±SEM; two-way ANOVA followed by Bonferroni's post hoc multiple comparison tests. Source data are provided as a Source Data file. AAV: adeno-associated virus; circSCMH1: circular RNA SCMH1; *Plpp3*/LPP3 : lipid phosphate phosphatase 3; FTO: fat mass and obesity-associated protein; m^{6}A: *N*^{6}-methyladenosine; PT: photothrombotic.

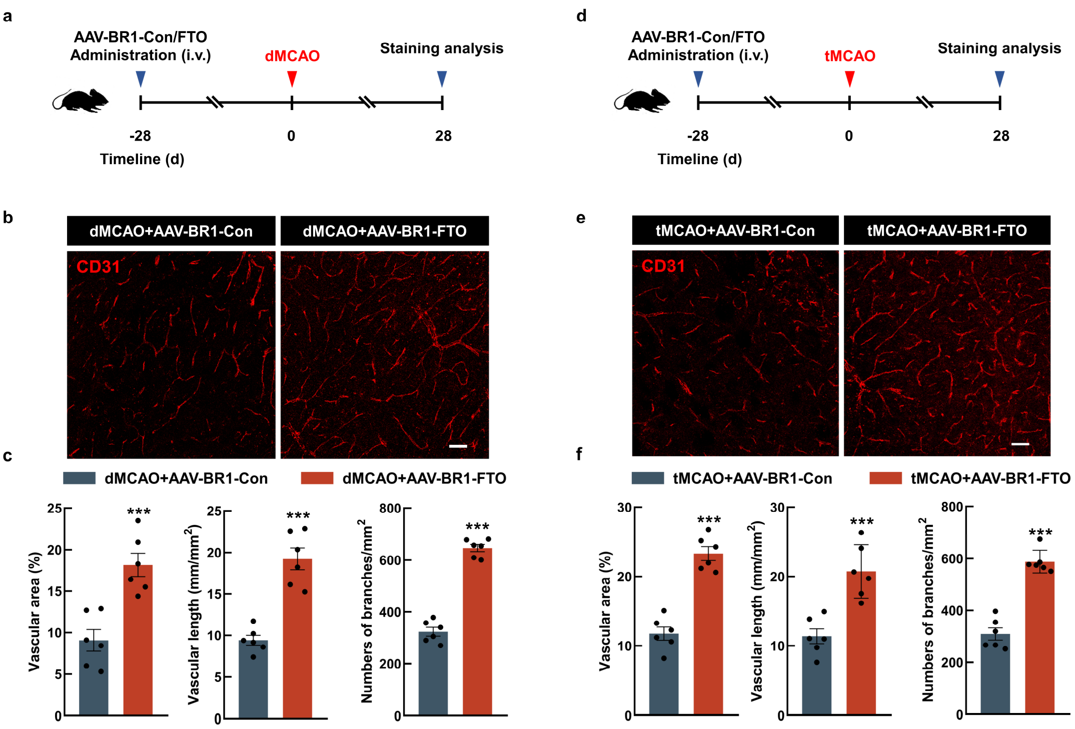

Supplementary Fig. 13 Endothelial-target FTO overexpression promoted vascular repair in dMCAO or tMCAO mice.

a, Schematic of AAV-BR1-FTO administration and staining analysis in dMCAO mice. **b** and **c**, Representative images with CD31 staining showing blood vessels in the peri-infarct cortex at day 28 after dMCAO model induction, followed by the analysis of vascular area fraction, total vascular length, and number of branches. n=6 mice/group. Scale bars, 50 µm. $^{***}P$=0.0008 (Vascular area), $^{***}P$<0.0001 (Vascular length and Numbers of branches) versus dMCAO+AAV-BR1-Con. **d**, Schematic of AAV-BR1-FTO administration and staining analysis in tMCAO mice. **e** and **f**, Representative images with CD31 staining showing blood vessels in the peri-infarct cortex at day 28 after tMCAO model induction, followed by the analysis of vascular area fraction, total vascular length, and number of branches. n=6 mice/group. Scale bars, 50 µm. $^{***}P$<0.0001 (Vascular area and Numbers of branches), $^{***}P$=0.0006 (Vascular length) versus tMCAO+AAV-BR1-Con. The data in **c** and **f** were expressed as means±SEM; the Student *t* test (two-sided). Source data are provided as a Source Data file. AAV: adeno-associated virus; CD31: platelet endothelial cell adhesion molecule-1; d: day; dMCAO: distal middle cerebral artery occlusion; FTO: fat mass and obesity associated protein; tMCAO: transient middle cerebral artery occlusion.

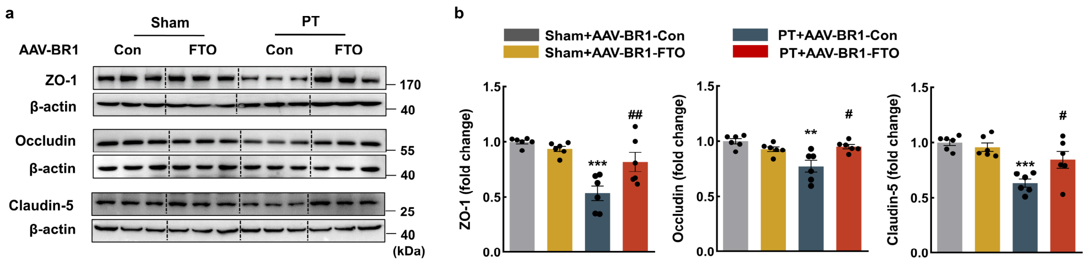

Supplementary Fig. 14 Levels of tight junction proteins ZO-1, Occludin, and Claudin-5 in mice injected with AAV-BRI-FTO at day 28 after PT stroke.

a and **b**, The expression of ZO-1, Occludin, and Claudin-5 was detected in PT mice with AAV-BRI-FTO. Three representative immunoblots were presented from 6 mice/group. $^{**}P=0.0022$ (Occludin), $^{***}P<0.0001$ (ZO-1), $^{***}P=0.0002$ (Claudin-5) versus sham+AAV-BR1-Con, $^{\#}P=0.0156$ (Occludin), $^{\#}P=0.0214$ (Claudin-5), $^{\#\#}P<0.0030$ (ZO-1) versus PT+AAV-BR1-Con. The data in **b** were expressed as the mean±SEM; two-way ANOVA followed by Bonferroni's post hoc multiple comparison tests. Source data are provided as a Source Data file. AAV: adeno-associated virus; FTO: fat mass and obesity-associated protein; PT: photothrombotic; ZO-1: zona occludens 1.

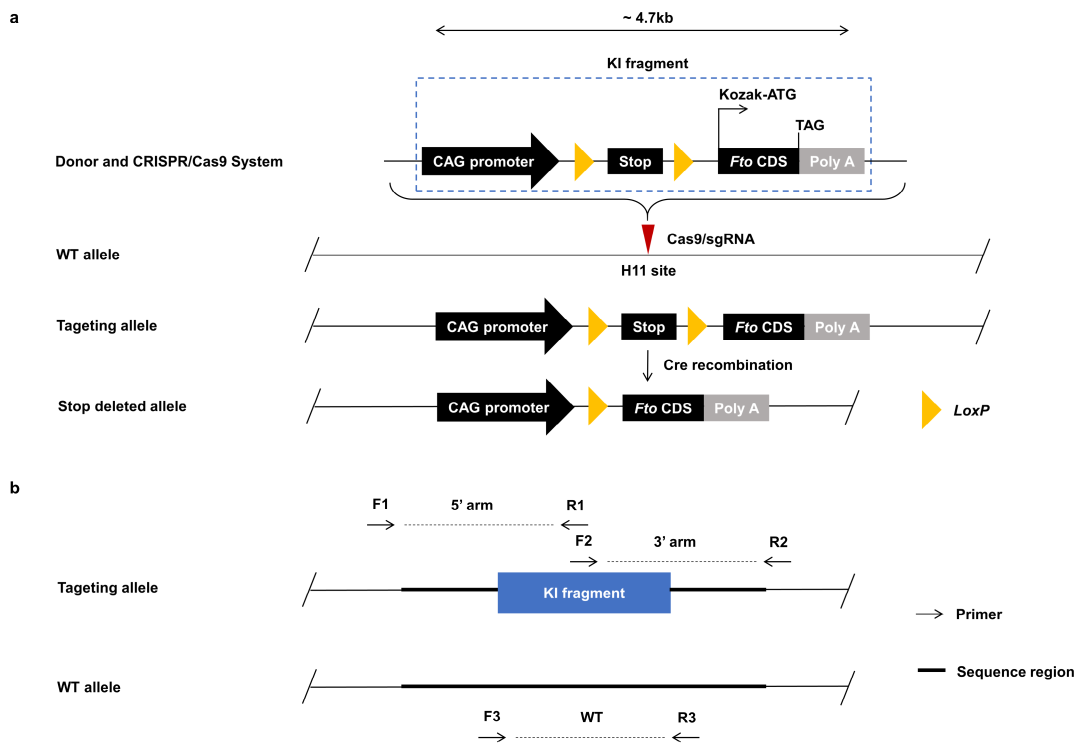

Supplementary Fig. 15 Generation and identification of conditional *Fto*^{flox} cKI mice.

a, Schematic of the targeting construct used to create *Fto*^{flox} cKI mice. For initial characterization, *Fto*^{flox} cKI mice were injected with AAV-BR1-Cre. **b**, Schematic of the identification of *Fto*^{flox} cKI mice. AAV: adeno-associated virus; Cre: cyclization recombination; CRISPR: clustered regularly interspaced short palindromic repeats; FTO: fat mass and obesity-associated protein; H11: *Hippo 11*; sgRNA: single-guide RNA; WT: wild type.

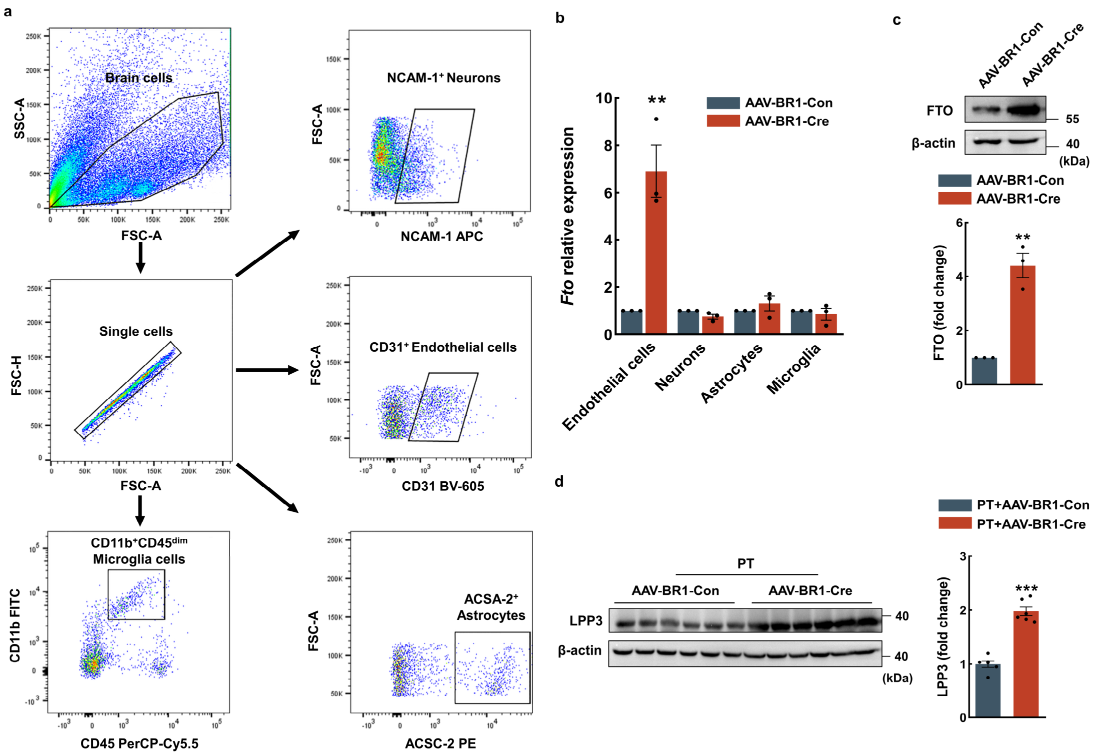

Supplementary Fig. 16 Levels of FTO and LPP3 in *Fto*$^{flox/flox}$ cKI mice injected with AAV-BRI-Cre.

a, Schematic of neurons, astrocytes, microglia, and endothelial cells isolation from PT stroke mice. **b**, The neurons, astrocytes, microglia, and ECs were isolated from total brain of *Fto*$^{flox/flox}$ cKI mice injected with AAV-BR1-Con/Cre for 4 weeks. Relative expression of *Fto* was determined by qPCR. n=3 samples for each group. Each sample was pooled from 3 animals. $^{**}P$=0.0059 versus AAV-BR1-Con. **c**, The microvessels were collected from three *Fto*$^{flox/flox}$ cKI mice injected with AAV-BR1-Con/Cre for 4 weeks. The expression of FTO was measured by western blot analysis in microvessels. Data were presented by 3 independent experiments. $^{**}P$=0.0018 versus AAV-BR1-Con. **d**, The expression of LPP3 was detected in *Fto*$^{flox/flox}$ cKI mice injected with AAV-BR1-Con/Cre at day 28 after PT. The representative immunoblots were presented from 6 mice/group. $^{***}P$<0.0001 versus PT+AAV-BR1-Con. The data in **b**, **c** and **d** were expressed as means±SEM; the Student *t* test (two sided). Source data are provided as a Source Data file. AAV: adeno-associated virus; Cre: cyclization recombination; FTO: fat mass and obesity-associated protein; LPP3: lipid phosphate phosphatase 3; PT: photothrombotic.

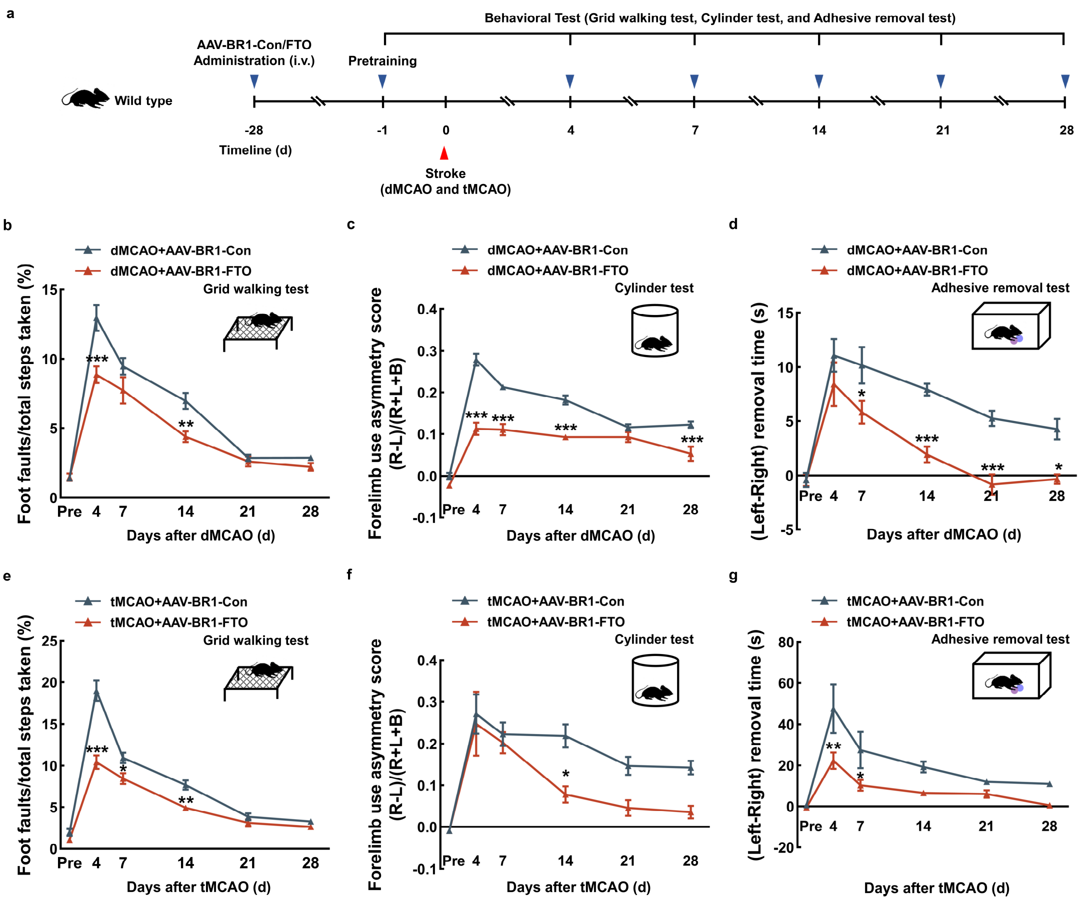

Supplementary Fig. 17 Endothelial-targeted FTO overexpression promoted motor functional recovery in dMCAO and tMCAO mice.

a, Schematic of AAV-BR1-FTO administration and behavioral analysis in dMCAO or tMCAO mice. **b**, **c** and **d**, Endothelial-targeted FTO overexpression improved behavioral recovery at different time points after dMCAO as measured by the grid-walking test, cylinder test and adhesive removal test. L indicates left forepaw in cylinder test; R, right forepaw in cylinder test; B, both forepaws in cylinder test. n=12 mice for dMCAO+AAV-BR1-Con group and dMCAO+AAV-BR1-FTO group. $^{*}P$=0.0182 (adhesive removal test 7d), $^{*}P$=0.0150 (adhesive removal test 28d), $^{**}P$=0.0034 (grid-walking test 14d), $^{***}P$<0.0001 (grid-walking test 4d, cylinder test 4d, 7d, 14d and 28d), $^{***}P$=0.0009 (adhesive removal test 14d and 21d) versus dMCAO+AAV-BR1-Con. **e**, **f** and **g**, Endothelial-targeted FTO overexpression improved behavioral recovery at different time points after tMCAO as measured by the grid-walking test, cylinder test and adhesive removal test. L indicates left forepaw in cylinder test; R, right forepaw in cylinder test; B, both forepaws in cylinder test. n=12 mice for tMCAO+AAV-BR1-Con group and tMCAO+AAV-BR1-FTO group. $^{*}P$=0.0122 (grid-walking test 7d), $^{*}P$=0.0117 (cylinder test 14d), $^{*}P$=0.0470 (adhesive removal test 7d), $^{**}P$=0.0058 (grid-walking test 14d), $^{**}P$=0.0012 (adhesive removal test 4d), $^{***}P$<0.0001 (grid-walking test 4d) versus tMCAO+AAV-BR1-Con. The data in **b**, **c**, **d**, **e**, **f** and **g** were expressed as the mean±SEM; two-way repeated measures ANOVA followed by Holm-Sidak post hoc multiple comparison test. Source data are provided as a Source Data file. AAV: adeno-associated virus; circSCMH1: circular RNA SCMH1; d: day; dMCAO: distal middle cerebral artery occlusion; FTO: at mass and obesity-associated protein; tMCAO: transient middle cerebral artery occlusion; PT: photothrombotic.
