# Supplementary material for: FTO-dependent m6A modification of Plpp3 in circSCMH1-regulated vascular repair and functional recovery following stroke
Source: Nat Commun. 2023 Jan 30;14:489. doi: 10.1038/s41467-023-36008-y (PMC9886939; doi:10.1038/s41467-023-36008-y)
Supplement: Supplementary file 2 — Reporting Summary [file 41467_2023_36008_MOESM2_ESM.pdf]

nature portfolio

Corresponding author(s): Jie Chao, Bing Han, Honghong Yao

Last updated by author(s): Jan 4, 2023

Reporting Summary

Nature Portfolio wishes to improve the reproducibility of the work that we publish. This form provides structure for consistency and transparency in reporting. For further information on Nature Portfolio policies, see our Editorial Policies and the Editorial Policy Checklist.

Statistics

For all statistical analyses, confirm that the following items are present in the figure legend, table legend, main text, or Methods section.

n/a	Confirmed	
☐	☒	The exact sample size (*n*) for each experimental group/condition, given as a discrete number and unit of measurement
☐	☒	A statement on whether measurements were taken from distinct samples or whether the same sample was measured repeatedly
☐	☒	The statistical test(s) used AND whether they are one- or two-sided<br>*Only common tests should be described solely by name; describe more complex techniques in the Methods section.*
☒	☐	A description of all covariates tested
☐	☒	A description of any assumptions or corrections, such as tests of normality and adjustment for multiple comparisons
☐	☒	A full description of the statistical parameters including central tendency (e.g. means) or other basic estimates (e.g. regression coefficient) AND variation (e.g. standard deviation) or associated estimates of uncertainty (e.g. confidence intervals)
☐	☒	For null hypothesis testing, the test statistic (e.g. *F*, *t*, *r*) with confidence intervals, effect sizes, degrees of freedom and *P* value noted<br>*Give P values as exact values whenever suitable.*
☒	☐	For Bayesian analysis, information on the choice of priors and Markov chain Monte Carlo settings
☒	☐	For hierarchical and complex designs, identification of the appropriate level for tests and full reporting of outcomes
☐	☒	Estimates of effect sizes (e.g. Cohen's *d*, Pearson's *r*), indicating how they were calculated

Our web collection on statistics for biologists contains articles on many of the points above.

Software and code

Policy information about availability of computer code

Data collection	We used the following software for data collection: ZEN2011, Olympus FV3000, HITACHI H-7650, Applied biosystems StepOne 4480845.
Data analysis	We used the following software for data analysis: DREME 4.12.0, Image J 1.53k, Angiotool 64 0.6a, Imaris x64 9.0.0, FlowJo software V10, GraphPad Prism 8.0.1.

For manuscripts utilizing custom algorithms or software that are central to the research but not yet described in published literature, software must be made available to editors and reviewers. We strongly encourage code deposition in a community repository (e.g. GitHub). See the Nature Portfolio guidelines for submitting code & software for further information.

Data

Policy information about availability of data

All manuscripts must include a data availability statement. This statement should provide the following information, where applicable:

- Accession codes, unique identifiers, or web links for publicly available datasets
- A description of any restrictions on data availability
- For clinical datasets or third party data, please ensure that the statement adheres to our policy

The MeRIP-sequencing data in this study have been deposited in the Gene Expression Omnibus database under accession code GSE193633 [https://www.ncbi.nlm.nih.gov/geo/query/acc.cgi?acc=GSE193633]. All the data supporting this study are available within the article, the Supplementary file, and the Source data file, as indicated in the Reporting summary for this article. Source data are provided with this paper.

Human research participants

Policy information about studies involving human research participants and Sex and Gender in Research.

Reporting on sex and gender	All postmortem brain samples from human were purchased from the Chinese Brain Bank Center (CBBC) (Wuhan, China) (http://www.cbbcnet.cn/). And the human research participant is not a sex/gender related research and we collected the samples from 10 male and 2 female patients. The disaggregated sex and gender data is provided in the sheets Figure 2a-c, S5a, S5d of source data.
Population characteristics	The acute ischemic stroke individual died by AIS, whereas nonstroke patients were died without evidence of stroke (active malignant diseases or neurological and psychiatric diseases). The detail information was listed in Supplementary Table 1.
Recruitment	Postmortem brain samples were purchased from the Chinese Brain Bank Center (CBBC) (Wuhan, China) (http://www.cbbcnet.cn/). The CBBC is jointly managed and operated by Tongji Medical College of Huazhong University of Science and Technology, and the Wuhan Institute of Neuroscience and Neural Engineering of South-Central Minzu University.
Ethics oversight	South-Central University for Nationalities Research Ethics and Safety Committee (2021-scuec-034)

Note that full information on the approval of the study protocol must also be provided in the manuscript.

Field-specific reporting

Please select the one below that is the best fit for your research. If you are not sure, read the appropriate sections before making your selection.

☒ Life sciences ☐ Behavioural & social sciences ☐ Ecological, evolutionary & environmental sciences

For a reference copy of the document with all sections, see nature.com/documents/nr-reporting-summary-flat.pdf

Life sciences study design

All studies must disclose on these points even when the disclosure is negative.

Sample size	Human data:<br>No statistical methods were used to pre-determine sample sizes. Frozen frontal cortex samples from six nonstroke patients and six acute ischemic stroke patients were used to experiments.<br><br>Monkey data:<br>Base on previous study between monkey and brain damage (Science. 2018 Apr 6;360(6384):50-57.) (Circulation 2020 Aug 11;142(6):556-574.), four monkeys/group were used for experiments (two groups: PT+EV-Vector, PT+EV-circSCMH1).<br><br>Mouse data:<br>The animal ethics requires minimizing the number of animals used. Based on our previous studies (Circulation 2020 Aug 11;142(6):556-574.), the number of mouse is not less than 6 for mouse experiments except long-term image analysis in Figure 1i-f and Figure 6f. For long-term image analysis, sample size is 3-4 mice. Sample size for each experiment is stated in the figure legends.<br><br>Cell data:<br>Base on our previous study in m6A analysis or ischemic stroke analysis(Biol Psychiatry 2020 Sep 1;88(5):392-404.)(Circulation 2020 Aug 11;142(6):556-574.), we used at least n = 3 replicates to calculate the statistical values for each analysis. Sample size for each experiment is stated in the figure legends.
Data exclusions	No data were excluded from the analyses.
Replication	All experiments were repeated and reliably reproduced. Number of mice used and independent experiments performed are indicated in the figure legends.
Randomization	Mice were randomly assigned to groups. Experimental units including mouse cages, culture wells were randomly organized in this study. Orders or treatments in regard to cage location for mouse experiments, and well location for cell culture experiments were random to avoid confounders.
Blinding	Behavioral tests were performed by an investigator blinded to the experimental groups. Gating strategies for flow cytometry analysis were kept the same for control and experimental groups. For quantifications of histological sections and WB, blinding was performed by labeling the sections numerically without prior knowledge of the treatment of the sample. The other samples and analyses were not blinded to the authors.

Behavioural & social sciences study design

All studies must disclose on these points even when the disclosure is negative.

Study description	*Briefly describe the study type including whether data are quantitative, qualitative, or mixed-methods (e.g. qualitative cross-sectional, quantitative experimental, mixed-methods case study).*
Research sample	*State the research sample (e.g. Harvard university undergraduates, villagers in rural India) and provide relevant demographic information (e.g. age, sex) and indicate whether the sample is representative. Provide a rationale for the study sample chosen. For studies involving existing datasets, please describe the dataset and source.*
Sampling strategy	*Describe the sampling procedure (e.g. random, snowball, stratified, convenience). Describe the statistical methods that were used to predetermine sample size OR if no sample-size calculation was performed, describe how sample sizes were chosen and provide a rationale for why these sample sizes are sufficient. For qualitative data, please indicate whether data saturation was considered, and what criteria were used to decide that no further sampling was needed.*
Data collection	*Provide details about the data collection procedure, including the instruments or devices used to record the data (e.g. pen and paper, computer, eye tracker, video or audio equipment) whether anyone was present besides the participant(s) and the researcher, and whether the researcher was blind to experimental condition and/or the study hypothesis during data collection.*
Timing	*Indicate the start and stop dates of data collection. If there is a gap between collection periods, state the dates for each sample cohort.*
Data exclusions	*If no data were excluded from the analyses, state so OR if data were excluded, provide the exact number of exclusions and the rationale behind them, indicating whether exclusion criteria were pre-established.*
Non-participation	*State how many participants dropped out/declined participation and the reason(s) given OR provide response rate OR state that no participants dropped out/declined participation.*
Randomization	*If participants were not allocated into experimental groups, state so OR describe how participants were allocated to groups, and if allocation was not random, describe how covariates were controlled.*

Ecological, evolutionary & environmental sciences study design

All studies must disclose on these points even when the disclosure is negative.

Study description	*Briefly describe the study. For quantitative data include treatment factors and interactions, design structure (e.g. factorial, nested, hierarchical), nature and number of experimental units and replicates.*
Research sample	*Describe the research sample (e.g. a group of tagged Passer domesticus, all Stenocereus thurberi within Organ Pipe Cactus National Monument), and provide a rationale for the sample choice. When relevant, describe the organism taxa, source, sex, age range and any manipulations. State what population the sample is meant to represent when applicable. For studies involving existing datasets, describe the data and its source.*
Sampling strategy	*Note the sampling procedure. Describe the statistical methods that were used to predetermine sample size OR if no sample-size calculation was performed, describe how sample sizes were chosen and provide a rationale for why these sample sizes are sufficient.*
Data collection	*Describe the data collection procedure, including who recorded the data and how.*
Timing and spatial scale	*Indicate the start and stop dates of data collection, noting the frequency and periodicity of sampling and providing a rationale for these choices. If there is a gap between collection periods, state the dates for each sample cohort. Specify the spatial scale from which the data are taken*
Data exclusions	*If no data were excluded from the analyses, state so OR if data were excluded, describe the exclusions and the rationale behind them, indicating whether exclusion criteria were pre-established.*
Reproducibility	*Describe the measures taken to verify the reproducibility of experimental findings. For each experiment, note whether any attempts to repeat the experiment failed OR state that all attempts to repeat the experiment were successful.*
Randomization	*Describe how samples/organisms/participants were allocated into groups. If allocation was not random, describe how covariates were controlled. If this is not relevant to your study, explain why.*
Blinding	*Describe the extent of blinding used during data acquisition and analysis. If blinding was not possible, describe why OR explain why blinding was not relevant to your study.*

Did the study involve field work? ☐ Yes ☐ No

Field work, collection and transport

Field conditions	*Describe the study conditions for field work, providing relevant parameters (e.g. temperature, rainfall).*
Location	*State the location of the sampling or experiment, providing relevant parameters (e.g. latitude and longitude, elevation, water depth).*
Access & import/export	*Describe the efforts you have made to access habitats and to collect and import/export your samples in a responsible manner and in compliance with local, national and international laws, noting any permits that were obtained (give the name of the issuing authority, the date of issue, and any identifying information).*
Disturbance	*Describe any disturbance caused by the study and how it was minimized.*

Reporting for specific materials, systems and methods

We require information from authors about some types of materials, experimental systems and methods used in many studies. Here, indicate whether each material, system or method listed is relevant to your study. If you are not sure if a list item applies to your research, read the appropriate section before selecting a response.

Materials & experimental systems

n/a	Involved in the study	
☐	☒	Antibodies
☐	☒	Eukaryotic cell lines
☒	☐	Palaeontology and archaeology
☐	☒	Animals and other organisms
☒	☐	Clinical data
☒	☐	Dual use research of concern

Methods

n/a	Involved in the study	
☒	☐	ChIP-seq
☐	☒	Flow cytometry
☒	☐	MRI-based neuroimaging

Antibodies

Antibodies used

WB:
ZO-1 (1:1000, 21773-1-AP , Proteintech group),
Occludin (1:1000, 27260-1-AP, Proteintech group),
Claudin-5 (1:1000, AF5216, Affinity),
Beta-actin (1:3000, 66009-1-Ig, Proteintech group),
Histone H3 (1:3000, 17168-1-AP, Proteintech group),
HRP-conjugated goat anti-mouse IgG secondary antibody (1:2000, SA00001-1, Proteintech group),
HRP-conjugated goat anti-rabbit IgG secondary antibody (1:2000, SA00001-2, Proteintech group),
FTO (1:1000, 227226-1-AP, Proteintech group),
Ub-K63 (1:2000, CY6579, Abways),
PPAP2B Polyclonal Antibody (1:1000, PA5-90665, Invitrogen),
METTL3 (1:1000, 15073-1-AP, Proteintech group),
METTL14 (1:1000, 26158-1-AP, Proteintech group),
WTAP (1:1000, 10200-1-AP, Proteintech group),
ALKBH5 (1:1000, 16837-1-AP, Proteintech group),
Synaptophysin (1:2000, 17785-1-AP, Proteintech group),
CD31 (1:2000, 28083-1-AP, Proteintech group),
UBC13 (1:1000, 10243-1-AP, Proteintech group).

Flow Cytometry:
Brilliant Violet 605 anti-mouse CD31 (1ul: 2x10^5 cells, 102427, BioLegend),
APC anti-Mouse NCAM-1/CD56 Allophycocyanin MAb (1ul: 2x10^5 cells, FAB7820A-100, R&D),
PE anti-mouse ACSA-2 (1ul: 2x10^5 cells, 130-116-244, Miltenyi Biotec),
FITC anti-mouse/human CD11b Antibody (1ul: 1x10^6 cells, 101205, BioLegend),
PerCp-CyTM5.5 anti-mouse CD45 Antibody (1ul: 1x10^6 cells, 561869, BD Pharmingen),

IF:
CD31 Polyclonal Antibody (1:100, PA5-32321, Invitrogen) for mouse IF,
Anti-mouse CD31 (1:50, ab9498, abcam) for monkey IF,
BrdU(1:100, 史称SC51514, SantaCruz),
Biotinylated-Isolectin B4 (1:200, L2140, Sigma),
Mouse Aminopeptidase N/CD13 Antibody (1:400, AF2335, R&D),

Streptavidin-FITC secondary antibody (1:100, 434311, Invitrogen),
Alexa 488-conjugated goat anti-mouse IgG (1:300, A11001, Invitrogen),
Alexa 594-conjugated goat anti-mouse IgG (1:300, A11005, Invitrogen),
Alexa 488-conjugated goat anti-rabbit IgG (1:300, A11008, Invitrogen),
Alexa 594-conjugated goat anti-rabbit IgG (1:300, A11002, Invitrogen).

IP:
FTO (8 ug: 5x10^6, 227226-1-AP, Proteintech group),
UBC13 (8 ug: 5x10^6, 10243-1-AP, Proteintech group).

Validation

All antibodies are available on the manufacturer's websites. The antibodies have been validated by the manufacturers. No additional validation was carried out.

Eukaryotic cell lines

Policy information about cell lines and Sex and Gender in Research

Cell line source(s)

HEK293T cells (SCSP-502) were kindly provided by Cell Bank/Stem Cell Bank, Chinese Academy of Sciences.
The mouse brain endothelial cell line bEnd3 was purchased from ATCC (CRL-2299, RRID:CVCL-0170).
Primary human microvascular endothelial cells were purchased from iCell Bioscience (HUM-icell-n001, iCell Bioscience)
Primary brain microvascular endothelial cells of the cerebral cortex were obtained from C57BL/6J mice from 8 weeks.

Authentication

The mouse brain endothelial cell line bEnd3, primary brain microvascular endothelial cells and primary human microvascular endothelial cells were authenticated by immunofluorescent staining of CD31.
HEK293t cells were not authenticated.

Mycoplasma contamination

All cells were negative for mycoplasma.

Commonly misidentified lines
(See ICLAC register)

No commonly misidentified cell lines were used in this study.

Palaeontology and Archaeology

Specimen provenance

Provide provenance information for specimens and describe permits that were obtained for the work (including the name of the issuing authority, the date of issue, and any identifying information). Permits should encompass collection and, where applicable, export.

Specimen deposition

Indicate where the specimens have been deposited to permit free access by other researchers.

Dating methods

If new dates are provided, describe how they were obtained (e.g. collection, storage, sample pretreatment and measurement), where they were obtained (i.e. lab name), the calibration program and the protocol for quality assurance OR state that no new dates are provided.

☐ Tick this box to confirm that the raw and calibrated dates are available in the paper or in Supplementary Information.

Ethics oversight

Identify the organization(s) that approved or provided guidance on the study protocol, OR state that no ethical approval or guidance was required and explain why not.

Note that full information on the approval of the study protocol must also be provided in the manuscript.

Animals and other research organisms

Policy information about studies involving animals; ARRIVE guidelines recommended for reporting animal research, and Sex and Gender in Research

Laboratory animals

Adult male C57BL/6J mice (7-8 weeks old) on a C57BL/6J background and FTO flox/flox conditional knock in mice on a C57BL/6J background, were purchased from the GemPharmatech (Nanjing, China). All mice were co-housed in the same room under similar condition (temperature approximately 21 ℃degree centigrade, humidity approximately 50%, 14 hours/10 hours: daylight/night cycle). Mice were given access to ad libitum water and food (SFS9112, Xietong Shengwu).
Male rhesus monkeys (Macaca mulatta, 5-9 years in age) were housed in adjoining individual primate cages, and the animal house was controlled for humidity (approximately 60%), temperature (approximately 21 degree centigrade), and light (12 hours/12 hours: daylight/night cycle) at the Kunming Institute of Zoology (Kunming, China). Monkeys had access to tap water and were punctually supplied with food three times a day.

Wild animals

No wild animals were used.

Reporting on sex

All animals were male on this study.

Field-collected samples	No field-collected samples were used.
Ethics oversight	All mouse experiments were approved by the Institutional Animal Care and Use Committee at the Medical School of Southeast University (approval ID 20200324001). and performed in accordance with the Animal Research: Reporting of In Vivo Experiments (ARRIVE) guidelines. Euthanasia was done first exposing mice to CO2 air and then followed by cervical dislocation. Researchers and monkeys care staff monitored the monkeys daily to ensure their health and welfare. Commercial primate diet and fresh fruits and vegetables were provided daily, and water was provided through an automatic watering system in each cage. Monkeys were anesthetized with hydrochloric acidulated ketamine (10 mg/kg, i.m.) and maintained with sodium pentobarbital (25 mg/kg, i.m.). They were then perfused with 4% paraformaldehyde in 1×PBS at PH 7.4 under deep anesthesia. All efforts were made to minimize pain and discomfort of the animals throughout the course of the study. In the current experiment, the euthanasia of the monkeys was approved by the IACUC at the Kunming Institute of Zoology, Chinese Academy of Sciences (IACUC No. 18016).

Note that full information on the approval of the study protocol must also be provided in the manuscript.

Clinical data

Policy information about clinical studies

All manuscripts should comply with the ICMJEguidelines for publication of clinical research and a completedCONSORT checklist must be included with all submissions.

Clinical trial registration	*Provide the trial registration number from ClinicalTrials.gov or an equivalent agency.*
Study protocol	*Note where the full trial protocol can be accessed OR if not available, explain why.*
Data collection	*Describe the settings and locales of data collection, noting the time periods of recruitment and data collection.*
Outcomes	*Describe how you pre-defined primary and secondary outcome measures and how you assessed these measures.*

Dual use research of concern

Policy information about dual use research of concern

Hazards

Could the accidental, deliberate or reckless misuse of agents or technologies generated in the work, or the application of information presented in the manuscript, pose a threat to:

No	Yes	
☐	☐	Public health
☐	☐	National security
☐	☐	Crops and/or livestock
☐	☐	Ecosystems
☐	☐	Any other significant area

Experiments of concern

Does the work involve any of these experiments of concern:

No	Yes	
☐	☐	Demonstrate how to render a vaccine ineffective
☐	☐	Confer resistance to therapeutically useful antibiotics or antiviral agents
☐	☐	Enhance the virulence of a pathogen or render a nonpathogen virulent
☐	☐	Increase transmissibility of a pathogen
☐	☐	Alter the host range of a pathogen
☐	☐	Enable evasion of diagnostic/detection modalities
☐	☐	Enable the weaponization of a biological agent or toxin
☐	☐	Any other potentially harmful combination of experiments and agents

ChIP-seq

Data deposition

☐ Confirm that both raw and final processed data have been deposited in a public database such as GEO.

☐ Confirm that you have deposited or provided access to graph files (e.g. BED files) for the called peaks.

Data access links
May remain private before publication.

For "Initial submission" or "Revised version" documents, provide reviewer access links. For your "Final submission" document, provide a link to the deposited data.

Files in database submission

Provide a list of all files available in the database submission.

Genome browser session
(e.g. UCSC)

Provide a link to an anonymized genome browser session for "Initial submission" and "Revised version" documents only, to enable peer review. Write "no longer applicable" for "Final submission" documents.

Methodology

Replicates

Describe the experimental replicates, specifying number, type and replicate agreement.

Sequencing depth

Describe the sequencing depth for each experiment, providing the total number of reads, uniquely mapped reads, length of reads and whether they were paired- or single-end.

Antibodies

Describe the antibodies used for the ChIP-seq experiments; as applicable, provide supplier name, catalog number, clone name, and lot number.

Peak calling parameters

Specify the command line program and parameters used for read mapping and peak calling, including the ChIP, control and index files used.

Data quality

Describe the methods used to ensure data quality in full detail, including how many peaks are at FDR 5% and above 5-fold enrichment.

Software

Describe the software used to collect and analyze the ChIP-seq data. For custom code that has been deposited into a community repository, provide accession details.

Flow Cytometry

Plots

Confirm that:

- [x] The axis labels state the marker and fluorochrome used (e.g. CD4-FITC).
- [x] The axis scales are clearly visible. Include numbers along axes only for bottom left plot of group (a 'group' is an analysis of identical markers).
- [ ] All plots are contour plots with outliers or pseudocolor plots.
- [ ] A numerical value for number of cells or percentage (with statistics) is provided.

Methodology

Sample preparation

The peri-infarct tissues of mice were collected and then temporarily placed on ice. Tissues were dissociated and digested for 1 hour at 37 ℃degree centigrade by Papain (2 mg/mL, LS003119, Worthington) in RPMI 1640 medium (C11875500BT, Gibico). The mixture was passed through a 70 µm nylon mesh. Dispersed cells were collected by centrifugation with 300 g for 10 minutes. The cell pellet was resuspended in 30 % Percoll density gradient (17089109, Cytiva) and centrifuged at 900 g for 25 minutes. Samples were blocked with FcR Blocking Reagent (130-092-575, Miltenyi Biotec) and resuspended in PBS containing 2 % FBS.

Instrument

BD FACSCelestaTM Flow Cytometer

Software

FlowJo software V10 was used to analyse data

Cell population abundance

All sorted samples were checked for post-sorting purity. The abundance of the sorted populations were >95%.

Gating strategy

Mouse endothelial cells were used for fluorescence acquisition and stained with Brilliant Violet 605 anti-mouse CD31 (1ul: 2x10^5 cells, 102427, BioLegend).
M藕色ouse neurons were used for fluorescence acquisition and stained with APC anti-Mouse NCAM-1/CD56 Allophycocyanin MAb (1ul: 2x10^5 cells, FAB7820A-100, R&D).
M藕色ouse astrocytes were used for fluorescence acquisition and stained with PE anti-mouse ACSA-2 (1ul: 2x10^5 cells, 130-116-244, Miltenyi Biotec).
Mouse microglia were used for fluorescence acquisition and stained with FITC anti-mouse/human CD11b Antibody (1ul: 1x10^6 cells, 101205, BioLegend) and PerCp-CyTM5.5 anti-mouse CD45 Antibody (1ul: 1x10^6 cells, 561869, BD Pharmingen),
Gating was determined based on the appropriate negative isotype stained controls.

- [x] Tick this box to confirm that a figure exemplifying the gating strategy is provided in the Supplementary Information.

Magnetic resonance imaging

Experimental design

Design type	*Indicate task or resting state; event-related or block design.*
Design specifications	*Specify the number of blocks, trials or experimental units per session and/or subject, and specify the length of each trial or block (if trials are blocked) and interval between trials.*
Behavioral performance measures	*State number and/or type of variables recorded (e.g. correct button press, response time) and what statistics were used to establish that the subjects were performing the task as expected (e.g. mean, range, and/or standard deviation across subjects).*

Acquisition

Imaging type(s)	*Specify: functional, structural, diffusion, perfusion.*
Field strength	*Specify in Tesla*
Sequence & imaging parameters	*Specify the pulse sequence type (gradient echo, spin echo, etc.), imaging type (EPI, spiral, etc.), field of view, matrix size, slice thickness, orientation and TE/TR/flip angle.*
Area of acquisition	*State whether a whole brain scan was used OR define the area of acquisition, describing how the region was determined.*

Diffusion MRI ☐ Used ☐ Not used

Preprocessing

Preprocessing software	*Provide detail on software version and revision number and on specific parameters (model/functions, brain extraction, segmentation, smoothing kernel size, etc.).*
Normalization	*If data were normalized/standardized, describe the approach(es): specify linear or non-linear and define image types used for transformation OR indicate that data were not normalized and explain rationale for lack of normalization.*
Normalization template	*Describe the template used for normalization/transformation, specifying subject space or group standardized space (e.g. original Talairach, MNI305, ICBM152) OR indicate that the data were not normalized.*
Noise and artifact removal	*Describe your procedure(s) for artifact and structured noise removal, specifying motion parameters, tissue signals and physiological signals (heart rate, respiration).*
Volume censoring	*Define your software and/or method and criteria for volume censoring, and state the extent of such censoring.*

Statistical modeling & inference

Model type and settings	*Specify type (mass univariate, multivariate, RSA, predictive, etc.) and describe essential details of the model at the first and second levels (e.g. fixed, random or mixed effects; drift or auto-correlation).*
Effect(s) tested	*Define precise effect in terms of the task or stimulus conditions instead of psychological concepts and indicate whether ANOVA or factorial designs were used.*

Specify type of analysis: ☐ Whole brain ☐ ROI-based ☐ Both

Statistic type for inference (See Eklund et al. 2016)	*Specify voxel-wise or cluster-wise and report all relevant parameters for cluster-wise methods.*
Correction	*Describe the type of correction and how it is obtained for multiple comparisons (e.g. FWE, FDR, permutation or Monte Carlo).*

Models & analysis

n/a	Involved in the study
☐	☐ Functional and/or effective connectivity
☐	☐ Graph analysis
☐	☐ Multivariate modeling or predictive analysis

Functional and/or effective connectivity	*Report the measures of dependence used and the model details (e.g. Pearson correlation, partial correlation, mutual information).*
Graph analysis	*Report the dependent variable and connectivity measure, specifying weighted graph or binarized graph, subject- or group-level, and the global and/or node summaries used (e.g. clustering coefficient, efficiency, etc.).*

Multivariate modeling and predictive analysis

Specify independent variables, features extraction and dimension reduction, model, training and evaluation metrics.
